# Very early environmental enrichment protects against apoptosis and improves functional recovery from hypoxic–ischemic brain injury

**DOI:** 10.3389/fnmol.2022.1019173

**Published:** 2023-02-07

**Authors:** Hoo Young Lee, Suk-Young Song, Jihye Hwang, Ahreum Baek, Dawoon Baek, Sung Hoon Kim, Jung Hyun Park, Sungchul Choi, Soonil Pyo, Sung-Rae Cho

**Affiliations:** ^1^Department of Rehabilitation Medicine, Seoul National University College of Medicine, Seoul National University Hospital, Seoul, Republic of Korea; ^2^National Traffic Injury Rehabilitation Hospital, Gyeonggi-do, Republic of Korea; ^3^Yonsei University College of Medicine, Seoul, Republic of Korea; ^4^Graduate Program of Biomedical Engineering, Yonsei University College of Medicine, Seoul, Republic of Korea; ^5^Department and Research Institute of Rehabilitation Medicine, Yonsei University College of Medicine, Seoul, Republic of Korea; ^6^Department of Rehabilitation Medicine, Yonsei University Wonju College of Medicine, Wonju, Republic of Korea; ^7^Department of Rehabilitation Medicine, Rehabilitation Institute of Neuromuscular Disease, Gangnam Severance Hospital, Yonsei University College of Medicine, Seoul, Republic of Korea; ^8^Neuracle Science Co. Ltd., Seoul, Republic of Korea; ^9^Brain Korea 21 PLUS Project for Medical Science, Yonsei University, Seoul, Republic of Korea; ^10^Rehabilitation Institute of Neuromuscular Disease, Yonsei University College of Medicine, Seoul, Republic of Korea

**Keywords:** environmental enrichment, voluntary exercise, stroke, apoptosis, Fas, neuroprotection

## Abstract

Appropriate rehabilitation of stroke patients at a very early phase results in favorable outcomes. However, the optimal strategy for very early rehabilitation is at present unclear due to the limited knowledge on the effects of very early initiation of rehabilitation based on voluntary exercise (VE). Environmental enrichment (EE) is a therapeutic paradigm for laboratory animals that involves complex combinations of physical, cognitive, and social stimuli, as well as VE. Few studies delineated the effect of EE on apoptosis in very early stroke in an experimental model. Although a minimal benefit of early rehabilitation in stroke models has been claimed in previous studies, these were based on a forced exercise paradigm. The aim of this study is to determine whether very early exposure to EE can effectively regulate Fas/FasL-mediated apoptosis following hypoxic–ischemic (HI) brain injury and improve neurobehavioral function. C57Bl/6 mice were housed for 2 weeks in either cages with EE or standard cages (SC) 3 h or 72 h after HI brain injury. Very early exposure to EE was associated with greater improvement in motor function and cognitive ability, reduced volume of the infarcted area, decreased mitochondria-mediated apoptosis, and decreased oxidative stress. Very early exposure to EE significantly downregulated Fas/FasL-mediated apoptosis, decreased expression of Fas, Fas-associated death domain, cleaved caspase-8/caspase-8, cleaved caspase-3/caspase-3, as well as Bax and Bcl-2, in the cerebral cortex and the hippocampus. Delayed exposure to EE, on the other hand, failed to inhibit the extrinsic pathway of apoptosis. This study demonstrates that very early exposure to EE is a potentially useful therapeutic translation for stroke rehabilitation through effective inhibition of the extrinsic and intrinsic apoptotic pathways.

## 1. Introduction

Stroke is a leading cause of serious long-term disabilities, with more than 80% of cases of ischemic attack reported to have this outcome (Katan and Luft, 2018; Li et al., 2018; Bernardo-Castro et al., 2021). Ischemic stroke results in a sudden neurological deficit that includes impaired motor response, cognitive ability, and communication and mood; directly reducing the patients’ quality of life and adding an increasingly heavy burden on their family and community (Lee et al., 2017).

Acute brain ischemia triggers an “ischemic cascade” of pathophysiological events that includes impaired energy metabolism, excitotoxicity, oxidative stress, inflammation, and apoptosis; ultimately resulting in neuronal cell death. Ample evidence supports the critical role of apoptosis in the pathophysiology of acute brain ischemia, resulting in a significant loss of brain cells (Lee et al., 2017; Radak et al., 2017). Whereas the ischemic core of the brain experiences a sudden reduction of blood flow just minutes after the ischemic attack that results in irreversible injury and subsequent cell death, apoptosis within the ischemic penumbra may occur after several hours or days, and may be reversible (Radak et al., 2017; Tuo et al., 2022). Therefore, the inhibition of apoptosis may be a promising therapeutic strategy, and research on potential anti-apoptotic mechanisms could be important for the development of novel therapies.

Ischemic stroke triggers two main apoptotic pathways: The intrinsic pathway is initiated by the disruption of mitochondria and the release of cytochrome c, which is mediated by members of the B cell lymphoma/leukemia-2 (Bcl-2) family such as anti-apoptotic protein Bcl-2 and the pro-apoptotic protein Bcl-2-associated X protein (Bax) (Surgucheva et al., 2008; Tuo et al., 2022). On the other hand, the extrinsic pathway is triggered by signaling receptors in the plasma membrane, including tumor necrosis factor (TNF)-receptor 1, apoptosis antigen-1 (APO1/Fas/CD95), and TNF-related apoptosis-inducing ligand receptor (TRAIL-R) (Bhardwaj and Aggarwal, 2003; Tuo et al., 2022). In addition, the incidence of ischemic stroke is highly associated with the expression of Fas, Fas-associated death domain protein (FADD) and caspase-8 (Muhammad et al., 2018). Furthermore, the Fas signaling pathway has been suggested to be a critical inducer of apoptotic signals during acute cerebral ischemia (Rosenbaum et al., 2000; Mahovic et al., 2013; Chelluboina et al., 2014).

The optimal timing to initiate rehabilitation after stroke has yet to be established, although evidence increasingly suggests the benefit of organized and interprofessional rehabilitation within the first 2 weeks after the ischemic event (Powers et al., 2019). However, mounting evidence suggests that commencing high-dose, very early mobilization within 24 h of stroke onset may adversely affect patient outcomes (Sundseth et al., 2014; Yelnik et al., 2017; Tong et al., 2019). Most clinical and preclinical studies investigating exercise-induced effects after stroke used the forced exercise (FE) paradigm (Svensson et al., 2016). The results of the A Very Early Rehabilitation Trial after stroke (AVERT) trial demonstrated that the outcome of a high-dose and forced mobilization protocol within 24 h of stroke onset was less favorable than that obtained *via* the usual care protocol (Langhorne et al., 2017). In animal models of stroke, FE during the very early phase exacerbated brain damage, increased apoptotic cell death, and delayed functional recovery (Kozlowski et al., 1996; Schallert et al., 1997; Humm et al., 1998; Li et al., 2017a,b). However, the effects of very early exposure to environmental enrichment (EE), which includes voluntary exercise (VE), on neuroprotection and functional recovery have yet to be fully investigated.

EE focuses on voluntary physical activity and non-stressful conditioning through the provision of a larger space with various objects that serve as stimuli such as toys, tunnels, and running wheels, allowing greater levels of social interaction and stimulating rodent exploratory behavior (van Praag et al., 2000; Seo et al., 2018; Kim et al., 2021). While most of the studies investigating the potential mechanisms underlying the effects of EE focused on neurogenesis, synaptogenesis, or angiogenesis, the importance of neuronal survival has yet to be fully assessed (Leggio et al., 2005; Monteiro et al., 2014; Zhang et al., 2017). Few studies suggested the mediating effect of EE on extrinsic apoptosis in an experimental stroke model. Given the extremely early intervention and its voluntary nature, EE-mediated rehabilitation may become a useful strategy, not necessarily hindered by the adverse effects reported in other studies that focused on FE.

This study sought to determine the neuroprotective effects of very early exposure to EE following hypoxic–ischemic (HI) brain injury and to compare them with those resulting from delayed exposure to EE in adult mice. We describe the effects of altered expression of apoptosis-related genes on neuronal survival and functional recovery, and explore novel neuroprotective rehabilitation strategies after stroke with a focus on the self-motivated nature of EE.

## 2. Materials and methods

### 2.1. Induction of HI brain injury in experimental animals

Ischemic brain damage was induced in 6-week-old C57BL/6 mice *via* unilateral right carotid artery ligation under anesthesia with a mixture of Ketamine and Rompun. A mixture of Ketamine (100 mg/kg) and Rompun (10 mg/kg) was administered for each mouse based on their weight. Hypoxic brain injury (8% O_2_ for 30 min) following unilateral right carotid artery ligation was also generated as previously described (Yu et al., 2014; Parikh et al., 2016). To ensure that the HI brain injury had been successfully generated, H&E staining was conducted to confirm brain damage after sacrifice at the end of the study. Animals without damage in cerebral cortex, striatum, thalamus, and hippocampus by visual inspection of the microscopic images were excluded from this study.

### 2.2. Experimental procedures and cage conditions

**Figure 1:**
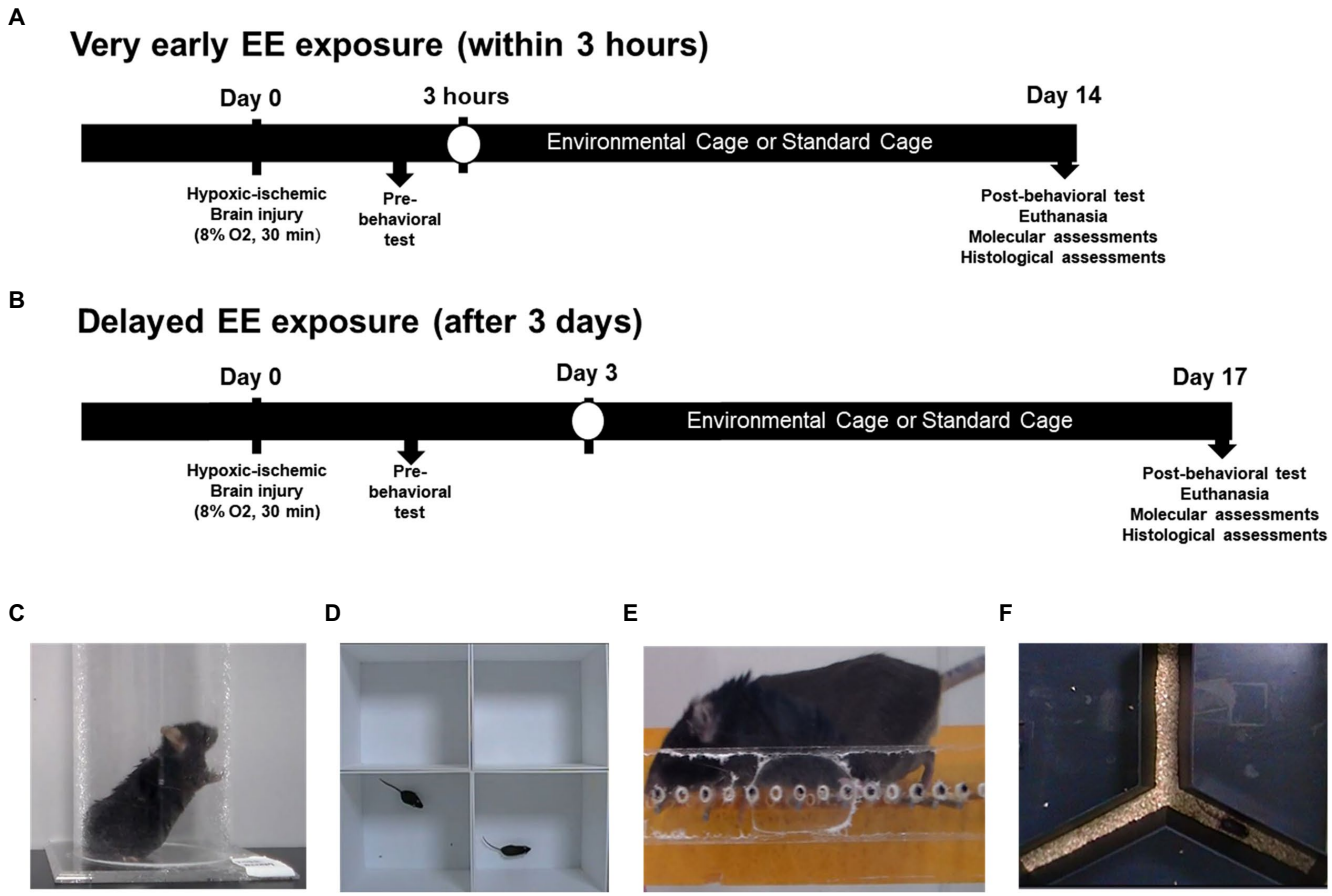
Schematic overview of the experimental design and behavioral assessments. **(A)** Very early exposure to EE. Within 3 h after HI brain injury, mice were exposed to either EE or SC for 14 days. **(B)** Delayed exposure to EE. Three days after HI brain injury, mice were exposed to either EE or SC for 14 days. **(C)** Cylinder test. **(D)** Open field test. **(E)** Ladder walking test. **(F)** Y-maze test. EE, environmental enrichment; HI, hypoxic–ischemic; SC, standard cage.

For the very early EE model, a total of 70 C57BL/6 mice were randomly housed in standard cages (SC) or in EE cages (*n* = 35 per group) within 3 h of exposure to HI brain injury for 2 weeks (Figure 1A). For the delayed EE model, a total of 30 C57BL/6 mice were randomly assigned to either SC or EE cages (*n* = 15 per group), 3 days after exposure to HI brain injury for 2 weeks (Figure 1B).

Mice assigned to EE were housed in a large cage (86 cm × 76 cm × 31 cm) containing novel objects such as tunnels, shelters, toys, and running wheels for VE and social interaction (12–15 mice/cage), whereas the control mice were housed in SCs (27 cm × 22.5 cm × 14 cm) without social interaction (4–5 mice/cage). All experiments were performed with C57BL/6 mice housed in a facility accredited by the Association for Assessment and Accreditation of Laboratory Animal Care and provided with food and water *ad libitum* under an alternating 12-h light/dark cycle, following standard regulations for animal husbandry. The experimental procedure was approved by the Institutional Animal Care and Use Committee of Yonsei University Health System (permit number: 2021-0182).

### 2.3. Behavioral assessment

Mice assigned to both EE and SC conditions underwent cylinder tests, open field test, ladder walking tests, and Y-maze tests immediately after HI brain injury induction and 14 days after the induction. For the very early EE model, fourteen mice per group were subjected to the cylinder test, thirteen to the open field test, eight to the ladder walking test, and twelve to the Y-maze test. A total of mice was used for the delayed exposure model as follows: For Cylinder test (*n* = 10 per group); for Open field test (*n* = 13 per group); for Ladder walking (*n* = 15 per group); for Y-maze test (*n* = 15 per group). All the subjects assigned to the delayed exposure model participated in the entire battery of behavioral tests.

#### 2.3.1. Cylinder test

The cylinder test is designed to assess poststroke limb use asymetry in the animal disease model (Figure 1C; Balkaya et al., 2013; Kim et al., 2021). Mice were placed in a transparent plexiglass cylinder measuring 8 cm in diameter and 18 cm in height (Jeung Do B&P, Seoul, Korea), where they stood spontaneously and used their forepaws for support. The number of forelimbs touching the wall of the cylinder was counted in the standing position over a period of 5 min.

#### 2.3.2. Open field test

The open field test is generally used to evaluate locomotor activity and anxiety in a novel environment (Figure 1D; Kraeuter et al., 2019). Activity was monitored in an area measuring 30 cm × 30.5 cm × 31 cm. Mice were placed individually in the periphery of the area and allowed to freely explore it for 25 min, while being monitored with a video camera. The resulting data were analyzed using the Smart Vision 2.5.21 (Panlab, Barcelona, Spain) video tracking system. The total distance traveled by each mouse was used as an indicator of hyperactivity (Nishigaki et al., 2018).

#### 2.3.3. Ladder walking test

The ladder rung walking task can be used to measure subtle disturbances in motor function based on qualitative and quantitative analyses of skilled walking (Figure 1E; Metz and Whishaw, 2009). During the test, the mice were required to walk three times on a one-meter horizontal ladder equipped with metal rungs (Jeung Do B&P) located at various distances. The number of slips from the transverse rungs with each forelimb were measured with a videotape. The experimental groups were compared by measuring the difference in the percentage of slips on the transverse rungs of the ladder relative to the total number of steps taken with each forelimb.

#### 2.3.4. Y-maze test

The Y-maze test is used to evaluate cognition and spatial working memory (Figure 1F; Wahl et al., 1992). This test was carried out in an enclosed Y-shaped maze (Jeung Do B&P). Mice normally visit the arms of the maze in a consecutive order, a behavioral pattern known as spontaneous alternation. The number of entries to each arm, spontaneous alternations, and percent alterations were recorded for 8 min. The percent alternation was calculated as follows:


$$\left[\begin{array}{l} \text{Number of spontaneous alternations}\\ /\left(\text{Number of total}\;\text{arm}\;\text{entries}-2\right) \end{array}\right]\times 100.$$

### 2.4. Transcriptome analysis

Brain regions were dissected based on the gross anatomy of the mouse brain atlas. Total RNA from the cerebral cortex and hippocampus was prepared using the TRIzol reagent (Invitrogen Life Technologies, Carlsbad, CA, United States) according to the manufacturer’s protocols. For quality control, RNA quality and quantity were evaluated *via* 1% agarose gel electrophoresis and the ratio of absorbance at 260 and 280 nm was determined with a Nanodrop spectrophotometer (Thermo Fisher Scientific, Waltham, MA, United States). The 260/280 nm ratio is used to determine the purity of RNA, with values from 1.8 to 2.0 considered to reflect pure quality.

RNA sequencing was performed by Macrogen Inc., (Seoul, Korea). The mRNA was transcribed into a library of templates. The successive cluster generation using the reagents was achieved using the Illumina^®^ TruSeq™ RNA Sample Preparation Kit (Lee et al., 2014). We performed the transcriptome analysis *via* RNA-seq and data handling procedures. The RNA-seq protocol was performed according to the manufacturer’s instructions. First, the TruSeq™ mRNA library construction was accomplished in eight steps: mRNA purification and fragmentation, synthesis of first-strand cDNA, synthesis of second-strand cDNA, end repair, single 3′ adenylation, ligation of adapters, enrichment of DNA fragments, and validation of enriched library.

Kyoto Encyclopedia of Genes and Genomes (KEGG) pathway mapping of the identified proteins was performed using the Database for Annotation, Visualization and Integrated Discovery (DAVID) software.^1^

### 2.5. Western blot

Brain lysates were isolated from the cerebral cortex and the hippocampus. Total protein was extracted from the pooled lysates, dissolved in Invitrogen™ NuPAGE™ LDS Sample Buffer and separated by 10% sodium dodecyl sulfate polyacrylamide gel electrophoresis (SDS-PAGE; Bio-Rad Laboratories, Richmond, CA, United States). The separated proteins were further transferred onto a 0.45 μm polyvinylidene difluoride (PVDF) membrane (Amersham Pharmacia Biotech, Little Chalfont, United Kingdom). The membrane was blocked in 5% bovine serum albumin with Tris-buffered saline containing 0.1% Tween 20 for 1 h at room temperature, followed by incubation with primary antibodies overnight at 4°C. Anti-Fas (Abcam, Cambridge, UK), anti-FADD (Santa Cruz, Dallas, TX, United States), anti-Caspase-8 (Cell Signaling Technology (CST), Danvers, MA, United States), anti-Cleaved Caspase-8 (CST), anti-Caspase-3 (CST), anti-Cleaved Caspase-3 (CST), anti-Drp1 (Abcam), anti-Bax (Santa Cruz), anti-Bcl2 (Santa Cruz), anti-iNOS (CST), anti-COX2 (CST), and anti-β-actin (Santa Cruz) were the primary antibodies (1:1000). After three washes with Tris-buffered saline containing 0.1% Tween 20, all membranes were incubated with horseradish peroxidase-conjugated secondary antibodies (1:5000; Santa Cruz) for 1 h at room temperature. Finally, the membrane signal was visualized using an Amersham ImageQuant 800 equipment (Amersham Pharmacia Biotech).

### 2.6. Measurement of infarct volume

The animals were sacrificed and perfused with 4% paraformaldehyde. The harvested brain tissue was cryo-sectioned at a 16 μm thickness along the coronal plane and stained with hematoxylin–eosin (H&E). H&E staining was performed in four sections, from the frontal pole to the midbrain, to measure the infarct volume. The sections were photographed using a digital camera and analyzed using ImageJ program. The infarct volumes of the lesion were expressed as a percentage of the volume of the structures from the contralateral hemisphere using the formula:


[(VC−VL)/VC]×100

Where VC is the volume of control hemisphere and VL denotes the volume of non-infarcted tissue in the lesioned hemisphere. The total volume of infarcted tissue in each brain was calculated as the sum of the volumes of infarcted tissue estimated for each of the four brain sections.

### 2.7. Immunohistochemistry

Sections of 16 μm thickness were cut along the coronal and sagittal plane of the collected brains, and immunohistochemical staining of four sections was performed over a range of >128 μm. Three images per area were selected from each mouse for analysis. Fluorometric terminal deoxynucleotidyl transferase dUTP nick-end labeling (TUNEL) assay (Promega, Madison, WI, United States) was conducted to analyze DNA fragmentation in cells from the cerebral cortex and hippocampus according to the manufacturer’s protocol. Images were acquired *via* fluorescent microscopy (LSM700) and positive cell death (μm^2^) with respect to DAPI area (/μm^2^) was measured using ZEN Imaging Software version 2.1 (Blue edition; Zeiss). To validate Fas/FasL pathway-related apoptosis, endogenous expression of MAP-2 and FADD in the cerebral cortex and hippocampus was measured using an anti-MAP-2 antibody (1:400, Abcam) and an anti-FADD antibody (1:400, Santa Cruz). The sections were incubated with Alexa Fluor® 488 goat anti-rabbit (1:400, Invitrogen) and Alexa Fluor^®^ 594 goat anti-mouse (1:400, Invitrogen) secondary antibodies, and covered with Vectashield^®^ mounting medium with 4C, 6-diamidino-2-phenylindole (DAPI; Vector, Burlingame, CA, United States). For the quantification of the immunohistochemistry images, the average value obtained from three images from each animal was used for analysis. Images of apoptotic and FADD-positive (FADD^+^) cells were obtained using a fluorescent microscope (LSM700), and positive apoptotic cells (μm^2^) with respect to DAPI area (/μm^2^), and FADD^+^ cells (μm^2^) with respect to MAP2-positive (MAP2^+^) area (/μm^2^) were quantified using ZEN Imaging Software version 2.1 (Blue edition; Zeiss). Furthermore, 3D images of the apoptotic cells were acquired using the same software.

### 2.8. Statistical analysis

All data were expressed as means ± SEM. The differences between the two groups were analyzed using Student’s t-test with SPSS statistical software (IBM, Armonk, NY; version 25.0). A *p* value <0.05 was considered statistically significant. All graphic artwork was produced using GraphPad Prism version 9 (GraphPad Software lnc., San Diego, CA, United States).

## 3. Results

### 3.1. Both very early and delayed exposure to EE decrease hyperactivity, exert anxiolytic effects, and improve fine motor and cognitive function after HI brain injury

**Figure 2:**
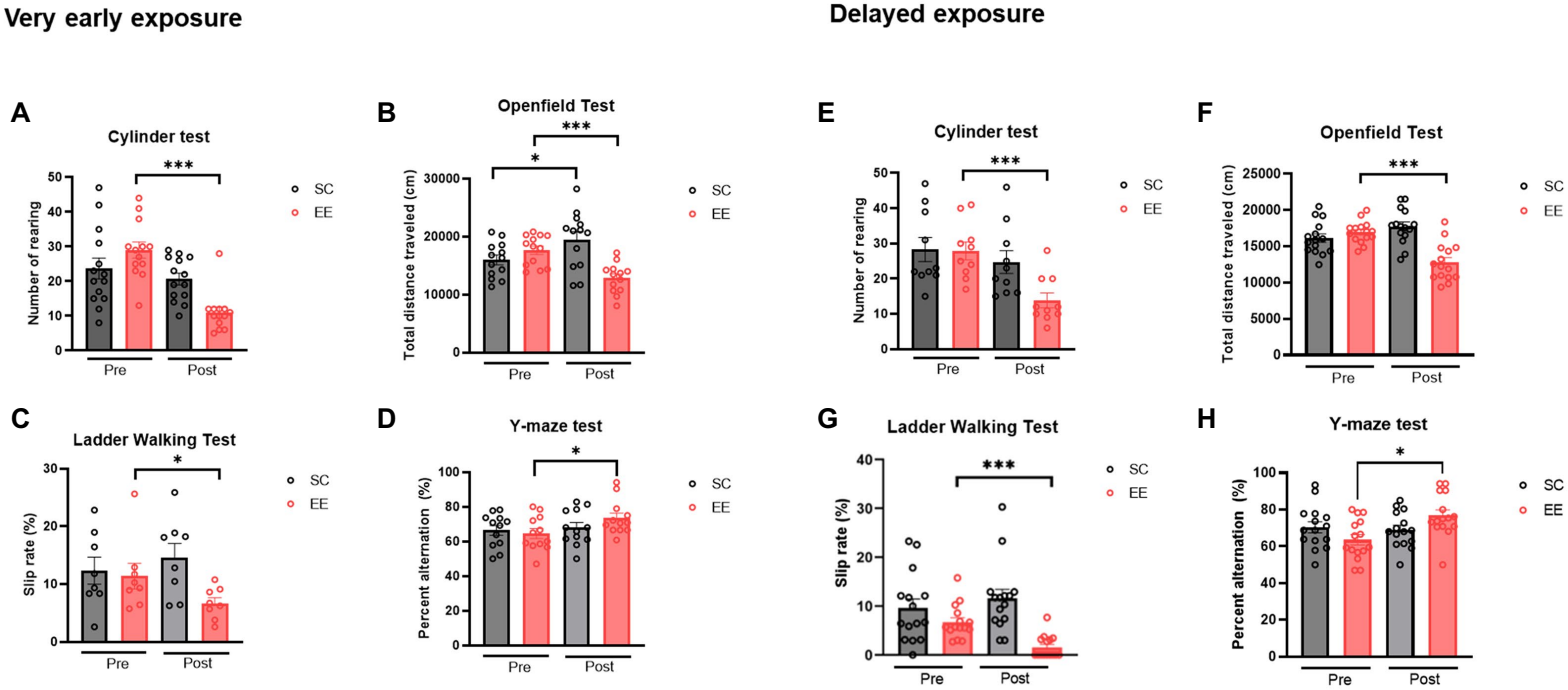
Quantitative analysis of the effect of very early and delayed exposure to EE on the neurobehavioral functions in mice after HI brain injury **(A–H)**. **(A)** Number of rearings in the cylinder test (*n* = 14 per group). **(B)** Total distance traveled in the open field test (*n* = 13 per group). **(C)** Percentage of total slips in the ladder walking test (*n* = 8 per group). **(D)** Percentage of spontaneous alternations in the Y-maze test (*n* = 12 per group). **(E–H)** Histograms depicting outcomes in the delayed exposure group and its respective control group. **(E)** Number of rearing in the cylinder test (*n* = 15 per group). **(F)** Total distance traveled in the open field test (*n* = 15 per group). **(G)** Percentage of total slips in the ladder walking test (*n* = 15 per group). **(H)** Percentage of spontaneous alternations in the Y-maze test (*n* = 15 per group). All statistical comparisons were performed *via* Student’s t-tests. Data represented are means ± SEM. *p < 0.05, **p < 0.01, and ***p < 0.001. EE, environmental enrichment; HI, hypoxic–ischemic.

In the cylinder test, the number of rearings was significantly reduced in the very early EE group (Figure 2A, *p* < 0.001), indicating that the hyperactivity derived from HI brain injury was significantly alleviated by very early exposure to EE. In the open field test, the total distance traveled was decreased in the very early EE group (Figure 2B, *p* < 0.001), indicating that the injury-induced hyperactivity and anxiety were significantly alleviated by very early exposure to EE. Moreover, the percentage of total slips was significantly reduced in the very early EE group (Figure 2C, *p* < 0.05), indicating that fine motor impairment was significantly alleviated by very early exposure to EE. In the Y-maze test, the percentage of alterations was significantly increased in the very early EE group (Figure 2D, *p* < 0.05) after the condition, indicating that cognitive function was significantly improved by very early exposure to EE.

The delayed EE group showed a significant reduction in the number of rearings displayed during the cylinder test (Figure 2E, *p* < 0.001), indicating that the hyperactivity derived from HI brain injury was significantly alleviated by delayed exposure to EE. In the open field test, the total distance traveled was significantly decreased in this group as well (Figure 2F, *p* < 0.001) after the condition, indicating that injury-induced hyperactivity and anxiety were significantly alleviated by delayed exposure to EE. Moreover, the percentage of total slips was significantly reduced in the delayed EE group (Figure 2G, *p* < 0.001), indicating that fine motor impairment was significantly alleviated by delayed exposure to EE. In the Y-maze test, the percentage of alterations was significantly increased in the delayed EE group (Figure 2H, *p* < 0.05) after the condition, indicating that cognitive function was significantly improved by delayed exposure to EE.

### 3.2. Very early and delayed exposures to EE decrease infarct size and apoptotic cells

**Figure 3:**
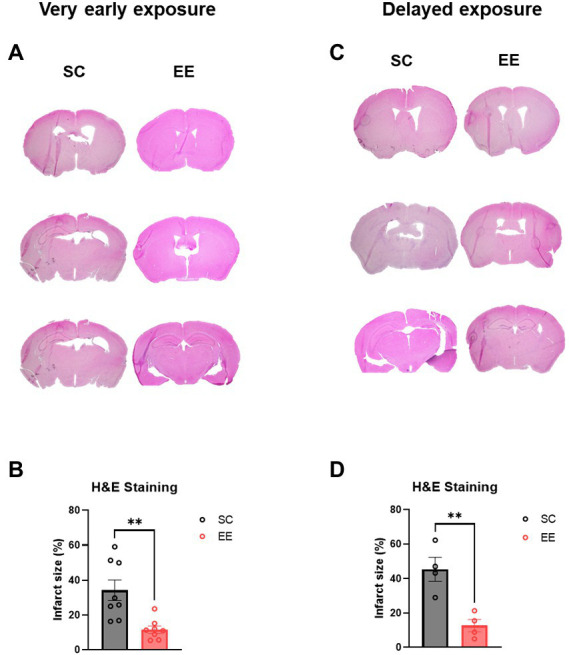
Histological assessments of the very early and delayed EE exposure groups. **(A,B)** Representative H&E images **(A)** and quantification of infarcted volume **(B)** after very early exposure to EE (*n* = 8 per group). **(C,D)** Representative H&E images **(C)** and quantification of infarcted volume **(D)** after delayed exposure to EE (*n* = 4 per group). H and E, hematoxylin–eosin.

The volume of the infarcted area was significantly decreased in the EE group (*p* < 0.01) compared to that from the control group, indicating that very early exposure to EE might have induced neuroprotection (Figure 3). The same result was observed for the delayed EE group (*p* < 0.01).

### 3.3. Very early exposure to EE downregulates genes associated with The extrinsic apoptotic pathway

From all the genes identified in the transcriptome analysis, we selected those showing at least a two-fold change compared to baseline levels (Differentially Expressed Genes, DEGs). A total of 1,691 DEGs (220 upregulated and 1,471 downregulated) in the cortex and 2,942 DEGs (866 upregulated and 2,076 downregulated) in the hippocampus in enriched mice were identified in mice with very early exposure to EE (Supplementary Tables S1, S2, respectively). In the delayed exposure to EE group, 489 DEGs (130 upregulated and 359 downregulated) in the cortex and 637 DEGs (139 upregulated and 498 downregulated) in the hippocampus were identified (Supplementary Tables S3, S4 respectively). However, there has been no significant result in delayed exposure to EE.

To further characterize the significance of the gene expression profiling, DEGs identified in the cortical and hippocampal regions and associated to a value of p below 10^−6^ were selected for further analysis. In the very early exposure to EE group, among the 1,471 downregulated DEGs in cortex and 2,076 downregulated DEGs in the hippocampus, 613 DEGs were selected and classified by KEGG pathway analysis using the DAVID Gene Functional Classification Tool (Supplementary Table S5). Significantly enriched KEGG pathways are indicated in Table 1 and Supplementary Table S6. Among several pathways, the mmu04210 (Apoptosis) pathway was statistically enriched in the very early exposure to EE group. In the delayed exposure to EE group, among 359 downregulated DEGs in the cortex and 498 downregulated DEGs in the hippocampus, only 17 DEGs were selected (Supplementary Table S7) but no significantly enriched pathway was identified in the analysis. Therefore, we focused on the genes involved in the apoptosis pathway identified in the very early exposure to EE group for further analyses.

**Table 1:** Enriched kyoto encyclopedia of genes and pathways in the very early exposure to EE group.

Term	*p* value	Benjamini-Hochberg adjusted *p* value	Term	*p* value	Benjamini-Hochberg adjusted *p* value
mmu04145:Phagosome	3.88E-21	9.96E-19	mmu05417:Lipid and atherosclerosis	1.97E-07	2.81E-06
mmu04610:Complement and coagulation cascades	9.73E-14	1.25E-11	mmu05330:Allograft rejection	2.60E-07	3.52E-06
mmu05152:Tuberculosis	1.15E-12	9.83E-11	mmu05169:Epstein–Barr virus infection	7.12E-07	8.89E-06
mmu05133:Pertussis	1.54E-12	9.92E-11	mmu05171:Coronavirus disease - COVID-19	7.27E-07	8.89E-06
mmu05140:Leishmaniasis	2.39E-12	1.07E-10	mmu04621:NOD-like receptor signaling pathway	2.07E-06	2.42E-05
mmu05150:Staphylococcus aureus infection	2.50E-12	1.07E-10	mmu05320:Autoimmune thyroid disease	3.91E-06	4.37E-05
mmu04640:Hematopoietic cell lineage	8.13E-11	2.99E-09	mmu05322:Systemic lupus erythematosus	5.23E-06	5.60E-05
mmu05323:Rheumatoid arthritis	1.51E-10	4.86E-09	mmu04512:ECM-receptor interaction	1.33E-05	1.37E-04
mmu05164:Influenza A	2.14E-09	6.10E-08	mmu04662:B cell receptor signaling pathway	2.78E-05	2.72E-04
mmu04060:Cytokine-cytokine receptor interaction	1.35E-08	3.46E-07	mmu04974:Protein digestion and absorption	2.86E-05	2.72E-04
mmu05332:Graft-versus-host disease	3.29E-08	7.46E-07	mmu05132:Salmonella infection	3.62E-05	3.32E-04
mmu04061:Viral protein interaction with cytokine and cytokine receptor	3.48E-08	7.46E-07	mmu05134:Legionellosis	5.35E-05	4.75E-04
mmu05416:Viral myocarditis	7.03E-08	1.39E-06	mmu04062:Chemokine signaling pathway	5.57E-05	4.78E-04
mmu04620:Toll-like receptor signaling pathway	7.68E-08	1.41E-06	mmu05321:Inflammatory bowel disease	6.19E-05	5.13E-04
mmu04612:Antigen processing and presentation	9.77E-08	1.67E-06	**mmu04210:Apoptosis**	**1.01E-04**	**8.08E-04**
mmu04380:Osteoclast differentiation	1.25E-07	2.00E-06	mmu04672:Intestinal immune network for IgA production	1.20E-04	9.36E-04
mmu04940:Type I diabetes mellitus	1.35E-07	2.05E-06			

The apoptosis pathway is shown in bold. These pathways are statistically significant with a Benjamini-Hochberg *p* < 0.001 according to database for annotation, visualization and integrated discovery (DAVID) analysis.

Table 2 shows a list of apoptosis-related genes that were significantly downregulated both in the cerebral cortex and the hippocampus of mice after HI brain injury and EE treatment. The presence of FAS and CASP8 on the list suggests that very early exposure to EE can alter the expression of genes associated with extrinsic apoptotic pathways in both the cortex and the hippocampus of mice exposed to EE.

**Table 2:** List of genes downregulated in the cerebral cortex and the hippocampus in response to very early exposure to EE after hypoxic–ischemic brain injury.

Gene symbol	Description	Fold change in cerebral cortex	Fold change in hippocampus
*Ctsl*	cathepsin L	−2.14	−3.72
*Casp8*	caspase 8	**−2.45**	**−6.24**
*Ctsd*	cathepsin D	−2.45	−8.19
*Il3ra*	interleukin 3 receptor, alpha chain	−2.81	−4.10
*Parp3*	poly (ADP-ribose) polymerase family, member 3	−2.81	−3.69
*Ctsc*	cathepsin C	−3.02	−5.72
*Tnfsf10*	tumor necrosis factor (ligand) superfamily, member 10	−3.06	−3.46
*Ctsz*	cathepsin Z	−3.20	−7.69
*Traf1*	TNF receptor-associated factor 1	−3.35	−5.59
*Ctsh*	cathepsin H	−3.60	−9.75
*Fas*	Fas (TNF receptor superfamily member 6)	**−3.79**	**−4.06**
*Ctss*	cathepsin S	−4.22	−8.25
*Csf2rb*	colony stimulating factor 2 receptor, beta, low-affinity (granulocyte-macrophage)	−6.94	−20.10
*Casp12*	caspase 12	−12.65	−10.24
*Csf2rb2*	colony stimulating factor 2 receptor, beta 2, low-affinity (granulocyte-macrophage)	−12.81	−12.73
*Bcl2a1b*	B cell leukemia/lymphoma 2 related protein A1b	−15.37	−42.18

Aapoptosis-related genes were shown in bold.

### 3.4. Very early and delayed exposure to EE regulate the expression of proteins related to the intrinsic apoptosis pathway in the cerebral cortex and the hippocampus

**Figure 4:**
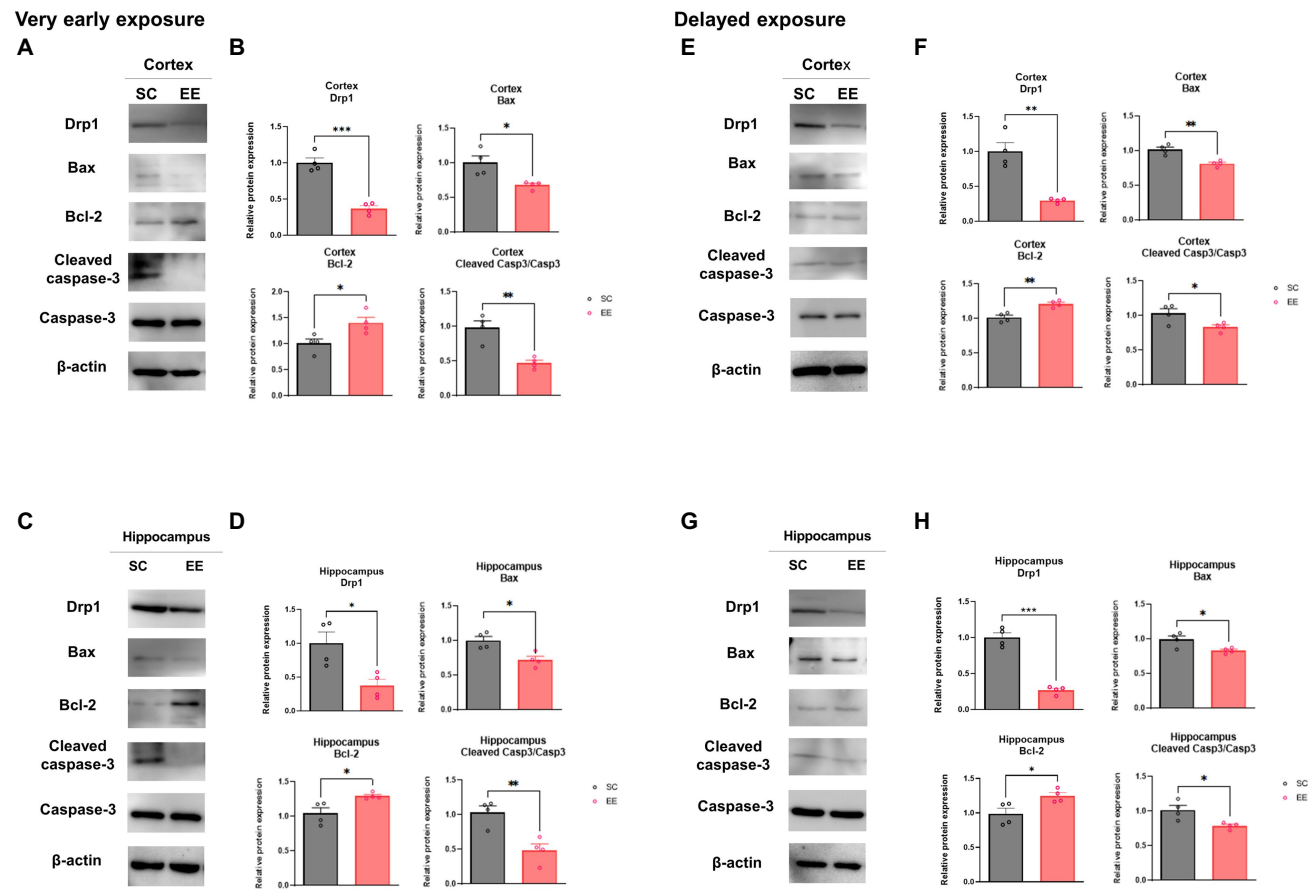
Effects of very early and delayed exposure to EE on the expression of mediators of mitochondrial dynamics and intrinsic apoptosis-related proteins in the cerebral cortex and the hippocampus. Representative WB images of the cerebral cortex **(A)** and the hippocampus **(C)** after very early exposure to EE. **(B,D)** Quantification of Drp1, Bax, Bacl-2, and cleaved Caspase-3 levels in the cerebral cortex **(B)** and the hippocampus **(D)** (*n* = 4 per group). **(E,G)** Representative WB images of the cerebral cortex **(E)** and the hippocampus **(G)** after delayed exposure to EE **(F,H)** Quantification of Drp1, Bax, Bacl-2, and cleaved Caspase-3/Caspase-3 levels in the cerebral cortex **(B)** and the hippocampus **(D)** (*n* = 4 per group). All statistical comparisons were performed *via* Student’s t-tests. Data represented are means ± SEM. *p < 0.05, **p < 0.01, and ***p < 0.001. EE, environmental enrichment; WB, western blot.

The levels of dynamin related protein 1 (Drp1), a key mediator of mitochondrial dynamics, were significantly decreased in the very early EE group (*p* < 0.001 and *p* < 0.05) compared to the control group in the cerebral cortex (Figure 4A,B) and the hippocampus (Figure 4C,D), respectively. Moreover, the levels of proteins related to the intrinsic apoptotic pathway such as Bax (*p* < 0.05 and *p* < 0.05), Bcl-2 (*p* < 0.05 and *p* < 0.05), and Cleaved Caspase 3 (*p* < 0.01 and *p* < 0.01) were significantly altered in the very early EE group compared to the control group in both regions (Figures 4A–D). Drp1 was also downregulated in the delayed EE group compared to the control group (*p* < 0.01) in the cerebral cortex (Figure 4E,F) and the hippocampus (*p* < 0.001) (Figure 4G,H); and the levels of Bax (*p* < 0.01 and *p* < 0.05), Bcl-2 (*p* < 0.01 and *p* < 0.05), and Cleaved Caspase 3 (*p* < 0.05 and *p* < 0.05) showed similar changes as those observed in the very early EE group compared to the control group, again in both brain regions (Figures 4E–H). These results indicate that both very early and delayed exposure to EE can regulate mitochondria dynamics and intrinsic apoptotic pathways.

### 3.5. Very early and delayed exposure to EE regulate the expression of oxidative stress in the cerebral cortex and the hippocampus

**Figure 5:**
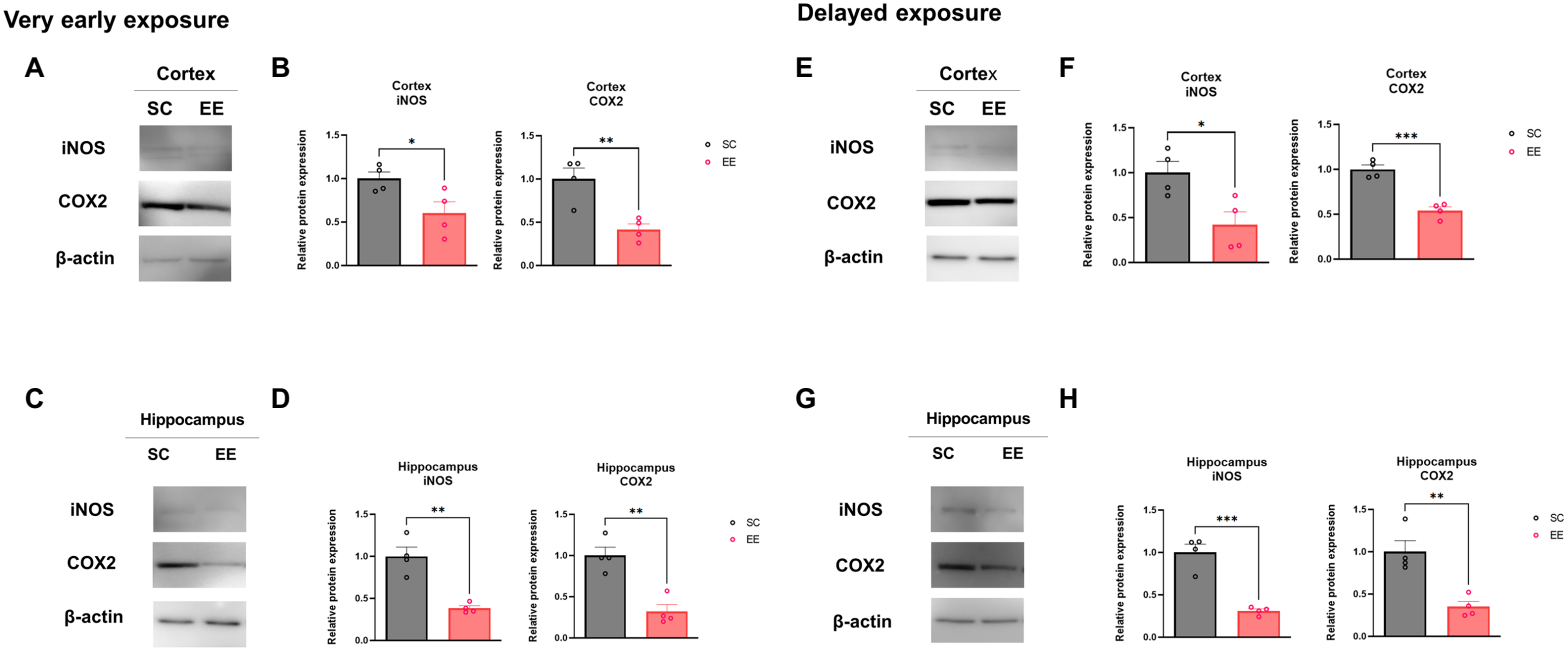
Effect of very early and delayed exposure to EE on iNOS and COX2 expression in the cerebral cortex and the hippocampus. Representative WB images from cerebral cortex and hippocampus obtained after very early EE exposure are shown in **(A,C)**, respectively. The quantification of the WB results is shown in **(B,D)**, respectively (*n* = 4 per group). Representative WB images from cerebral cortex and hippocampus obtained after delayed EE exposure are shown in **(E,G)**, respectively. The quantification of the WB results is shown in **(F,H)**, respectively (*n* = 4 per group). All statistical comparisons were performed *via* Student’s *t*-tests. Data represented are means ± SEM. *p < 0.05, **p < 0.01, and ***p < 0.001. EE, environmental enrichment; WB, western blot.

It is well known that HI brain injury increased mitochondrial reactive oxygen species (ROS) production (Qin et al., 2019). To investigate whether EE regulated the mitochondria-specific production oxidative stress, we examined the expression level of ROS generation related genes such as by WB. The expression level of genes associated with the production of ROS in the mitochondria, including cytochrome c oxidase subunit 2 (COX2) and inducible nitric oxide synthase (iNOS), were downregulated in both the very early and the delayed exposure to EE groups compared to their respective control groups in the cerebral cortex and the hippocampus (*p* < 0.05, *p* < 0.01 and *p* < 0.001; Figures 5A–H). These results suggest that both very early and delayed exposure to EE significantly attenuated the expression levels of genes associated with oxidative stress induced by HI brain injury.

### 3.6. Very early and delayed exposures to EE decrease apoptosis in the cerebral cortex and the hippocampus

**Figure 6:**
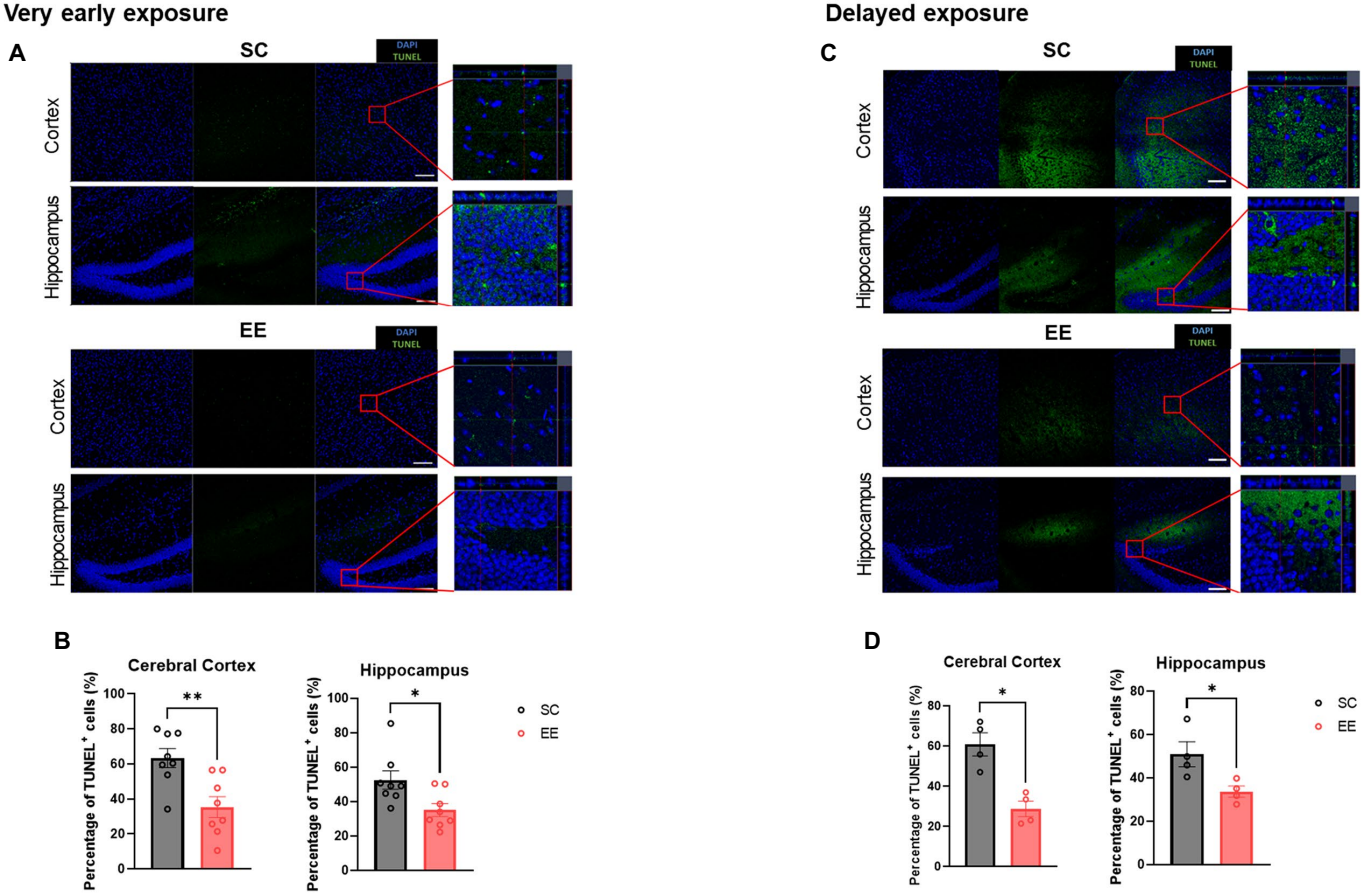
Very early and delayed exposure to EE decreases TUNEL immunopositivity in the cerebral cortex and the hippocampus. Representative TUNEL images from the very early exposure EE group and its control group are shown in **(A)**, and the corresponding quantification is shown in **(B)** (*n* = 8 per group). Representative TUNEL images from the delayed exposure group and its control group are shown in **(C)**, and the corresponding quantification is shown in **(D)** (*n* = 4 per group). All statistical comparisons were performed *via* Student’s t-tests. Data represented are means ± SEM. *p < 0.05, **p < 0.01, and ***p < 0.001. EE, environmental enrichment; TUNEL, terminal deoxynucleotidyl transferase dUTP nick-end labeling.

Representative TUNEL images from the very early exposure to EE group are shown in Figure 6A, and the quantification thereof is shown in Figure 6B. The percentage of TUNEL^+^ cells in the very early EE group was significantly decreased in the cerebral cortex (*p* < 0.01) and the hippocampus (*p* < 0.05, Figures 6A,B). The same result was observed in the case of the delayed exposure to EE group, again in both brain regions (*p* < 0.05 and *p* < 0.05, Figures 6C,D). These results indicate that, regardless of whether exposure to EE takes place very early or is delayed, apoptosis resulting from HI brain injury is ameliorated.

### 3.7. Very early exposure to EE, but not delayed EE, downregulates the expression of proteins associated with the extrinsic apoptosis pathway in the cerebral cortex and the hippocampus

**Figure 7:**
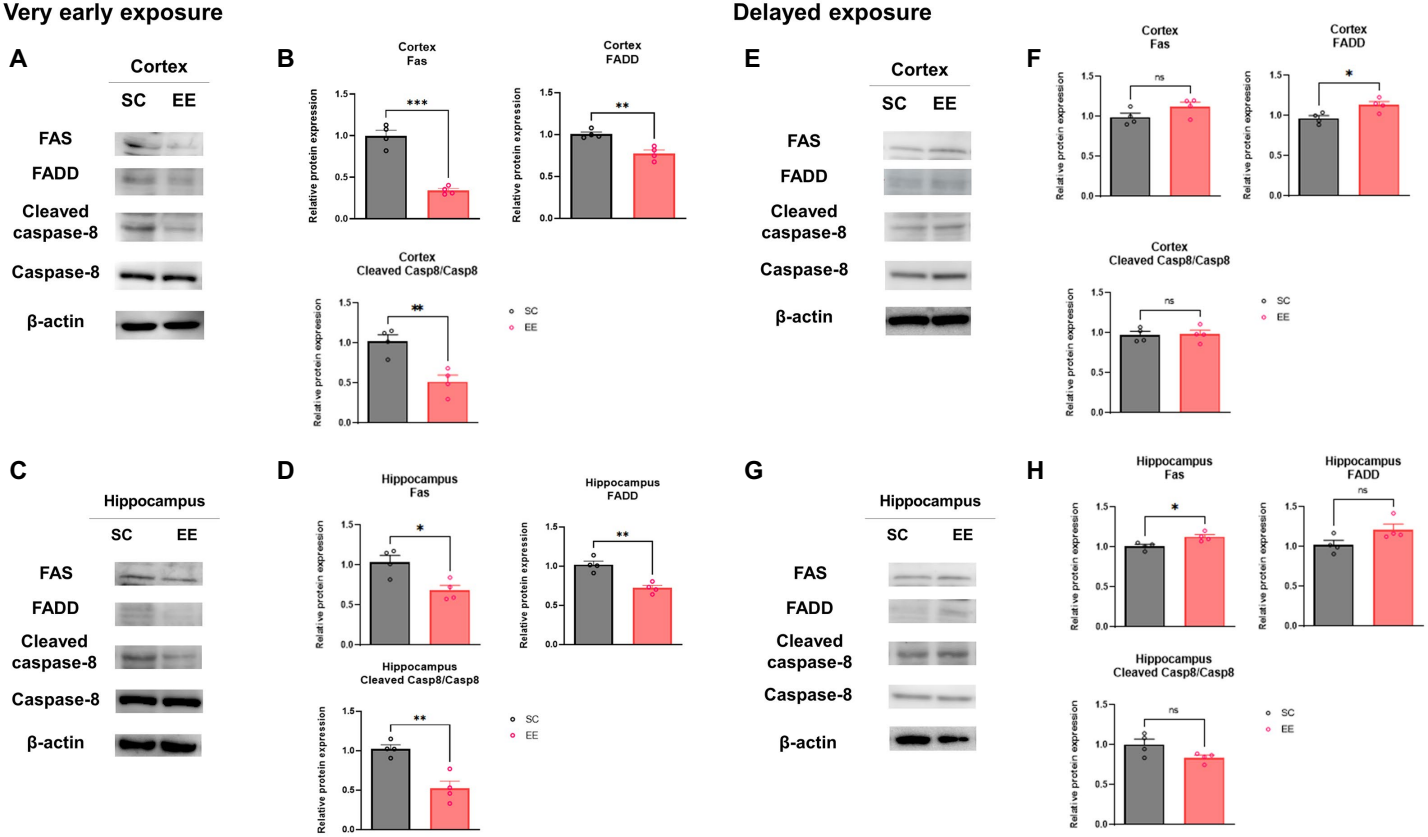
Effects of very early and delayed exposure to EE on protein expression in the extrinsic apoptosis pathway in the cerebral cortex and the hippocampus. **(A,C)** Representative WB images from cerebral cortex **(A)** and hippocampus **(C)** obtained after very early EE exposure. **(B,D)** Quantification of Fas, FADD, and cleaved Caspase-8 levels in the cerebral cortex **(B)** and the hippocampus **(D)** (*n* = 4 per group). **(E,G)** Representative WB images from cortical **(E)** and hippocampus **(G)** obtained after delayed EE exposure. **(F,H)** Quantification of Fas, FADD, and cleaved Caspase-8 in the cerebral cortex **(F)** and hippocampus **(H)** (*n* = 4 per group). All statistical comparisons were performed *via* Student’s *t*-tests. Data represented are means ± SEM. *p < 0.05, **p < 0.01, and ***p < 0.001. EE, environmental enrichment; WB, western blot.

The levels of proteins associated with the extrinsic apoptotic pathway, quantified by WB, were significantly decreased compared to the control group in the very early exposure to EE group in both the cerebral cortex and the hippocampus (Figures 7A–D). These included FAS (*p* < 0.001 and *p* < 0.05), FADD (*p* < 0.01 and *p* < 0.01), and Cleaved caspase-8 (*p* < 0.01 and *p* < 0.01). On the other hand, neither FAS nor FADD were significantly decreased in the delayed exposure to EE group compared to the control group, but Cleaved caspase-8 exhibited a significant decrease on its levels in both the cerebral cortex and the hippocampus (Figures 7E–H).

### 3.8. Very early but not delayed exposure to EE decreases MAP-2 and FADD colocalization

**Figure 8:**
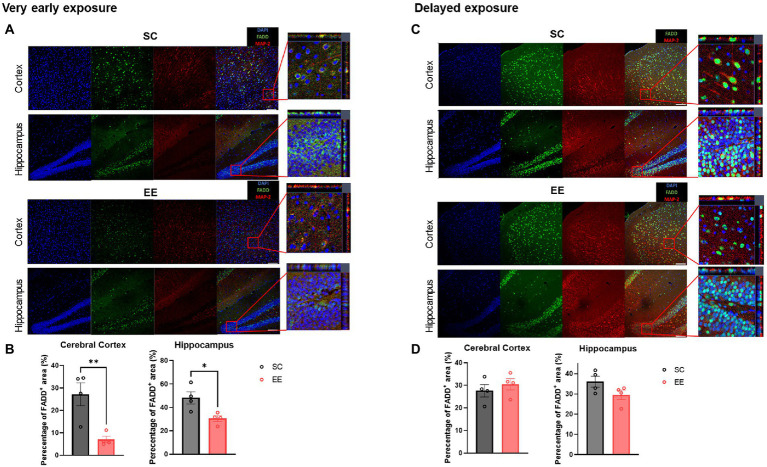
Very early, but not delayed, exposure to EE decreases colocalization of MAP-2 and FADD in the cerebral cortex and the hippocampus. **(A)** Representative images from the very early exposure to EE group and its control group depicting MAP-2 and FADD immunoreactivity. **(B)** Quantification of the percentage of colocalization (*n* = 4 per group). **(C)** Representative MAP-2 + FADD+ images from the delayed exposure to EE group and its control group depicting MAP-2 and FADD immunoreactivity. **(D)** Quantification of the percentage of colocalization (*n* = 4 per group). All statistical comparisons were performed *via* Student’s t-tests. Data represented are means ± SEM. *p < 0.05, **p < 0.01, and ***p < 0.001. EE, environmental enrichment.

The colocalization of MAP-2 and FADD was significantly decreased in the cerebral cortex (*p* < 0.01) and the hippocampus (*p* < 0.05) of experimental subjects from the very early exposure to EE group (Figures 8A,B). However, the colocalization of these two proteins in the cerebral cortex nor hippocampus showed no significant decrease in the delayed exposure to EE group (Figures 8C,D). These results indicate that very early exposure to EE, but not delayed exposure to EE, can alleviate apoptosis through the downregulation of extrinsic apoptotic pathways.

## 4. Discussion

Early post-stroke mobilization has been investigated extensively and is recommended in international clinical practice guidelines because early initiation of rehabilitation exercise (24 h to 72 h post-stroke) promotes functional recovery, reduces post-stroke complications, and allows for faster reintegration into society (Lee et al., 2009). However, previous studies have suggested that exposure to high doses of exercise at the early phase and forced non-VE within 24 h post-stroke adversely affect stroke outcomes due to incomplete cell death at the point of EE intervention (Garcia et al., 1993; Livingston-Thomas et al., 2016). However the irreversible nature of apoptosis should be taken into consideration, and an intensive and extremely early environmental intervention would be needed to halt this cascade and prevent further progress to the advanced apoptotic stage. This study emphasizes the critical need to prevent further apoptotic cell death in both the intrinsic and extrinsic pathways. The effects of very early commencement of VE and EE on functional recovery, and the underlying mechanisms after stroke, have yet to be fully elucidated.

**Figure 9:**
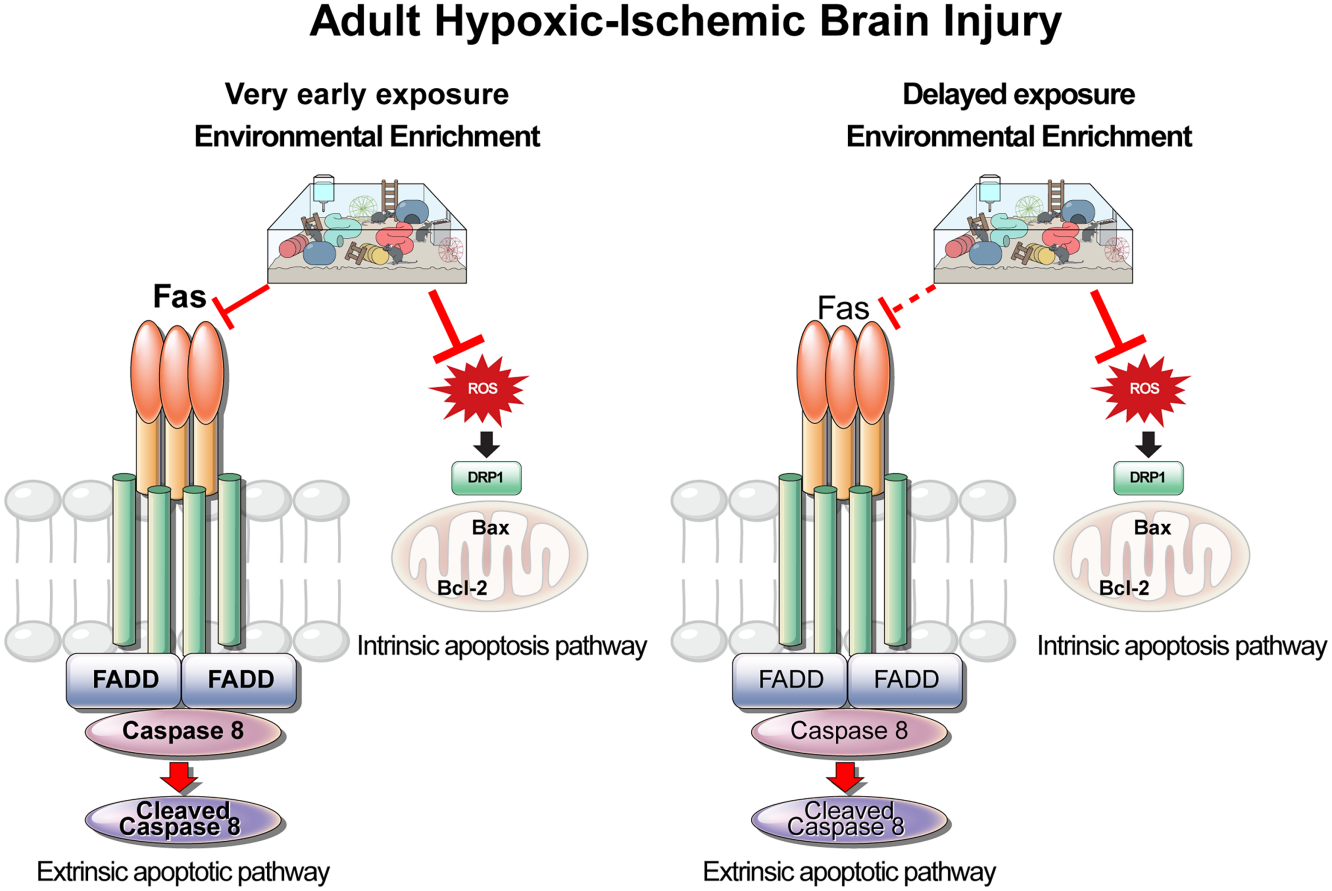
Schematic diagram of the extrinsic and intrinsic apoptosis pathways for very early and for delayed exposure to EE in the HI brain injury model. Solid lines indicate as activation and dashed arrows indicate as no activation. EE, environmental enrichment; HI, hypoxic–ischemic.

In contrast to previous studies, the current study focuses on the effects of exposure to EE on the inhibition of apoptosis during the very early phase of ischemic stroke. Very early exposure to EE significantly improved functional recovery and promoted neuronal survival. These neuroprotective effects were mediated *via* downregulation of both the extrinsic and intrinsic signaling pathways of apoptosis. A schematic diagram for the mechanism underlying the neuroprotection elicited by very early exposure to EE is shown in Figure 9. A significant downregulation of Fas/FasL-mediated apoptosis in the cerebral cortex and the hippocampus was detected in mice exposed to very early EE.

Previous studies have shown that VE may improve motor and cognitive ability, and have anti-apoptotic as well as neuroprotective effects (Mokhtari-Zaer et al., 2014; Xing et al., 2018). Compared with FE, VE is not associated with systemic stress and does not decrease the neuroprotective effect (Yanagita et al., 2007; Ke et al., 2011; Mokhtari-Zaer et al., 2014). VE *via* exploratory movements as provided by EE may have greater benefits (Biernaskie and Corbett, 2001). Early studies demonstrated the beneficial effects of VE on hippocampal function, which involved the suppression of cleaved Caspase-3 expression, decrease of Bax expression and increase of Bcl-2 expression in the hippocampus (Van Praag, 2009; Ko and Ko, 2020). Following stroke, VE may improve motor rehabilitation, enhance cognitive ability, and increase hippocampal BDNF expression compared with FE (Ke et al., 2011). The effects of post-stroke voluntary movements on neuronal regeneration and repair have been shown to be related to the upregulation of growth-associated protein 43 and neurotrophin 3 (Xing et al., 2018). A recent study reported that early commencement of VE post-stroke improved cerebral blood flow, vascular quality, and brain functions such as connectivity and motor abilities (Lohkamp et al., 2021).

Post-stroke FE training initiated at various time points is known to have a beneficial role (Lee et al., 2009). However, it was reported in a previous study that an early start of FE aggravated brain damage, enhanced apoptotic cell death, and triggered energy deficits associated with the generation of ROS (Li et al., 2017a). Many previous studies have indicated that ischemic stroke can elevate the expression of COX-2 (Iadecola and Gorelick, 2005; Wu et al., 2011). Moreover, COX-2 selective inhibitor has been used as a potential drug for ischemic stroke. However, it is reported that this type of drug has the limitations, inducing an increased risk of (recurrent) ischemic stroke (Andersohn et al., 2006). In this point of view, very early exposure to EE can be a potential therapy that can alleviate the elevated expression of COX-2 and apoptotic process in a safer way. The present study, on the other hand, highlights the benefits of EE, perhaps due to its voluntary nature, for the prevention of post-stroke apoptosis in the affected areas. In this sense, our results address the concerns raised by other researchers who conducted studies involving Fe using resources such as treadmill under the ambiguously defined EE.

Apoptosis may contribute significantly to neuronal death following brain ischemia; however, the therapeutic window for effective stroke rehabilitation through VE and the underlying mechanisms behind it are not fully understood. To our knowledge, the current study was the first of its kind to investigate the effect of very early exposure to EE on both extrinsic and intrinsic pathways of apoptosis in an adult mouse model of HI brain injury. Our data reveal that very early (within 3 h) exposure to EE post-stroke suppresses both extrinsic and intrinsic pathways of apoptosis in the cerebral cortex and the hippocampus. This may be due to the higher sensitivity of these brain areas to therapies that suppress neuronal apoptosis (Seo et al., 2014; Chen et al., 2017).

In a rodent model of ischemic stroke, acute treatment with edaravone was neuroprotective in transient focal ischemia, and the mechanism involved suppression of the Fas/FasL signaling pathway (Xiao et al., 2007). Recently, very early treatment with zonisamide was shown to decrease morbidity by suppressing the expression of caspase-3, caspase-8, and calpain-1, inhibiting the apoptosis of neuronal cells after cerebral ischemia (He et al., 2021). Further, intranasal administration of a Fas-blocking peptide 12 h after ischemic stroke attenuated Fas-mediated apoptosis, decreased the volume of the infarcted area, and reduced neurological deficits (Ullah et al., 2018). Importantly, a significant reduction in the volume of the infarcted area occurred in hybrid mice expressing nonfunctional Fas ligand and in TNF knockout mice 24 h after stroke (Martin-Villalba et al., 1999; Rosenbaum et al., 2000). However, the mechanism of suppression of extrinsic apoptosis mediated by EE treatment in the very early phase of ischemic stroke has yet to be described. In the present study, we found that very early exposure to EE significantly suppressed extrinsic apoptosis *via* downregulation of Fas/FasL-mediated signaling in both the cerebral cortex and the hippocampus, but delayed exposure to EE failed to have the same effect.

The Fas/FasL system plays an important role in apoptosis during the acute phase in other neurological disorders, and our findings are in agreement with previous studies (Furuichi et al., 2012; Tiao et al., 2014; Perez et al., 2017; Zhao et al., 2021). In a mouse model of traumatic brain injury, a peak in the levels of expression of Fas was noticed in the cortex and hippocampus 24 h after the injury (Grosjean et al., 2007). Furthermore, the levels of both Fas and FasL in cortical neurons and astrocytes were sustained for up to 72 h after injury (Beer et al., 2000). Fas-mediated apoptosis of neurons occurred in mouse models of acute and subacute spinal cord injury (SCI), and reduced apoptosis and neurological dysfunction were detected in Fas-deficient mice compared with control mice after SCI (Yu and Fehlings, 2011).

Drp1 is known as a pivotal factor of mitochondrial dynamics (Mao et al., 2021). Drp1 is a member of the dynamin superfamily of GTPases that regulates mitochondrial fission and apoptosis, and has been associated with several diseases. Drp1 binds to Bax to release pro-apoptotic factors (Shao et al., 2019). In the present study, we focused on the expression levels of Drp1 to investigate the details of mitochondrial physiology and its changes in response to EE. The results showed that both very early and delayed exposure to EE decreased the expression levels of Drp1, leading to reduction in pro-apoptotic markers in the cerebral cortex and the hippocampus.

The mitochondrial DNA (mtDNA) and oxidative stress have been implicated in the pathogenesis of neurodegenerative diseases, including Alzheimer’s disease, Parkinson’s disease, amyotrophic lateral sclerosis and Huntington’s disease (Lin and Beal, 2006). Moreover, the production of pathological ROS due to mitochondrial dysfunction has been demonstrated in an animal model for HI brain injury (Silachev et al., 2018). Thus, we investigated whether very early and delayed exposure to EE regulated mitochondria-associated oxidative stress. The results showed that the mitochondria-specific production of markers for oxidative stress, such as COX2 and iNOS, were markedly decreased in both very early and delayed exposure to EE in the cerebral cortex and the hippocampus.

This experiment was designed to use clinically relevant animal model for ischemic stroke (Adhami et al., 2006; Sun and Kuan, 2015; Feng et al., 2020). We exposed our experimental subjects to hypoxic condition following unilateral right carotid artery ligation (Yu et al., 2014). Through this mass sample cultivation procedure, the model was maintained of its result consistency in its HI brain injured samples. This thus reduced the extent of limitation arising from the model being a possible underrepresentation of general stroke pathology. While transient middle cerebral artery occlusion (MCAO) model is the most commonly used animal stroke model, it only represents approximately 10% of all large vessel occlusion stroke patients (McBride and Zhang, 2017; Feng et al., 2020). Unlike MCAO model, HI brain injury induces clinically relevant spontaneous thrombosis of blood vessels and neuronal death in the damaged regions of the brain (Sun and Kuan, 2015). The HI brain damage in the cerebral cortex, striatum, thalamus, and hippocampus was confirmed at 3 days post-HI brain injury using magnetic resonance imaging scans (Supplementary Figure S1).

The HI brain injury model used in this study may also represent HI brain injury after cardiac arrest (Sawyer et al., 2020). Unlike stroke, little attention has been paid to the timing of rehabilitation interventions in patients with HI brain injury after cardiac arrest. Currently, the strongest prevention practice remains early mobility (Boyce and Goossens, 2017). Early initiation of mobilization has been shown to decrease delirium and improve functional outcomes (Boncyk et al., 2019; Perkins et al., 2021). Further studies are needed to specify the appropriate starting point for rehabilitation treatment for HI brain injury following cardiac arrest.

Another limitation arises from the small sample associated with the molecular and histological findings (*n* = 4–8 per group), which restricts their generalization. Moreover, the diverse potential mechanisms of neuronal cell death contributing to long-term neuroprotection and functional recovery after stroke were not investigated, given their inherent complexity. We have not used aged animals in our study. Since stroke primarily affects elderly patients, it would be highly desirable to investigate the effects of very early exposure to EE in an aged animal model with multiple comorbidities to demonstrate the clinical relevance of the present findings for stroke rehabilitation. Therefore, it is highly desirable to investigate the effects of very early exposure to EE in an aged animal model with multiple comorbidities to demonstrate the clinical relevance of the present findings for stroke rehabilitation.

Current limitations of EE in the clinical setting include that the environment of acute stroke unit is generally unfavorable and the majority of stroke patients require frequent assistance from staff to undertake activities. (Rosbergen et al., 2017) implemented EE in an acute stroke unit by providing 1 h daily group sessions involving patient education, emotional support, communication and mobilization activities. And access to diverse equipment such as tablets, books, games, music was available at any time of day. Under this protocol, the EE group was significantly more engaged in “any” activities in physical, social, and cognitive domains and experienced significantly fewer adverse events such as falls, with no differences found in serious adverse events such as death.

To date, there are some limitations to better align preclinical and clinical EE. In human stroke rehabilitation it is much more challenging to standardize EE conditions because patient conditions including stroke lesion, age, sex, comorbidities, and length of stay vary, some patients require caregiver support owing to the medical and neurological condition, environment of acute stroke unit care is unfavourable, and due to cost restrictions (McDonald et al., 2018). Nonetheless, further efforts towards these challenges may advance implementation of EE into the clinical setting on a large scale and optimize delivery of EE for promoting recovery from stroke.

In summary, we have demonstrated that both very early exposure to EE and delayed exposure to EE after HI brain injury can induce improvement in behavioral outcomes, reduce the volume of the infarcted area, and mitochondria-mediated oxidative stress and apoptosis as intrinsic apoptotic pathway. Especially, very early exposure to EE reduces Fas/Fal mediated apoptosis as extrinsic apoptotic pathway. This study demonstrates the ability of very early EE intervention to prevent further progress of the apoptotic cascade. Overall, the results of this study demonstrate that, by effectively inhibiting the extrinsic as well as the intrinsic apoptotic pathway, very early exposure to EE is a promising candidate for therapeutic intervention after stroke.

## Data availability statement

The RNA-Seq data used in this study is deposited in the NCBI database. This data can be found here: https://www.ncbi.nlm.nih.gov/bioproject/PRJNA929036.

## Ethics statement

The animal study was reviewed and approved by Institutional Animal Care and Use Committee (IACUC) of Yonsei University Health System (permit number: 2021-0182).

## Author contributions

HL: study design, data interpretation, manuscript drafting, and revision. S-YS and SP: study design, data acquisition, and analysis. JH, AB, DB, SK, and JP: manuscript drafting and revision, data acquisition, data analysis, and intellectual discussion. SC: data collection and analysis. S-RC: funding acquisition, study design, data interpretation, manuscript editing and final revision. All authors contributed to the article and approved the submitted version.

## Funding

This research was supported by the National Research Foundation (NRF-5-2017-A0154-00395) grant funded by the Ministry of Science and Technology, Republic of Korea, the Ministry of Land, Infrastructure and Transport (MOLIT) Research Fund (NTRH RF-2021005), Republic of Korea, the Korean Fund for Regenerative Medicine (KFRM) grant funded by the Korea government (the Ministry of Science and ICT, the Ministry of Health and Welfare) (21A0202L1 and 21C0715L1) and the Korean Health Technology R&D Project through the Korea Health Industry Development Institute (KHIDI), funded by the Ministry of Health & Welfare, Republic of Korea (HI21C1314 and HI22C1588).

## Conflict of interest

SP was employed by Neuracle Science Co. Ltd.

The remaining authors declare that the research was conducted in the absence of any commercial or financial relationships that could be construed as a potential conflict of interest.

## Publisher’s note

All claims expressed in this article are solely those of the authors and do not necessarily represent those of their affiliated organizations, or those of the publisher, the editors and the reviewers. Any product that may be evaluated in this article, or claim that may be made by its manufacturer, is not guaranteed or endorsed by the publisher.

## Footnotes

^1^ http://david.abcc.ncifcrf.gov/
