# Supplementary material for: Very early environmental enrichment protects against apoptosis and improves functional recovery from hypoxic–ischemic brain injury
Source: Front Mol Neurosci. 2023 Feb 7;15:1019173. doi: 10.3389/fnmol.2022.1019173 (PMC9942523; doi:10.3389/fnmol.2022.1019173)
Supplement: Supplementary file 6 [file Table_6.DOCX]

**Supplementary Table 6. The enriched Kyoto Encyclopedia of Genes and Genomes pathways in the very early EE group.**

| Term | Count | Genes |
| --- | --- | --- |
| mmu04145:Phagosome | 41 | COLEC12, NCF1, NCF2, H2-M3, ITGB2, H2-Q7, NCF4, H2-K1, H2-Q4, TCIRG1, THBS2, THBS1, THBS4, CTSS, C3, MRC2, H2-DMB2, CLEC7A, CTSL, H2-DMB1, MRC1, H2-OB, CD36, CD14, C1RA, H2-EB1, CYBB, TAP1, CYBA, H2-AA, FCGR1, FCGR3, FCGR4, TLR6, ITGA5, FCGR2B, RAB7B, TLR4, H2-D1, TLR2, H2-AB1 |
| mmu04610:Complement and coagulation cascades | 24 | C1QB, C1QA, C1RA, CFH, PROS1,SERPINF2, C5AR1, ITGB2, F13A1, TFPI, C2, C3, C4B, THBD, C4A, PROCR, C1S1, PLAU, C3AR1, ITGAX, SERPING1, A2M, CFB, C1QC |
| mmu05152:Tuberculosis | 31 | ITGB2, TCIRG1, CTSS, C3, MRC2, H2-DMB2, CASP8, CLEC7A, H2-DMB1, MRC1, H2-OB, ITGAX, LBP, CD14, CTSD, CD74, H2-EB1, FCER1G, IL10RB, IL10RA, H2-AA, TLR1, FCGR1, IL1A, FCGR3, FCGR4, TLR6, FCGR2B, TLR4, TLR2, H2-AB1 |
| mmu05133:Pertussis | 21 | C1QB, C1QA, C1RA, TICAM2, ITGB2, CXCL5, C2, PYCARD, C3, C4B, IL1A, C4A, C1S1, CASP1, SERPING1, IRF8, NLRP3, ITGA5, CD14, TLR4, C1QC |
| mmu05140:Leishmaniasis | 20 | H2-EB1, NCF1, NCF2, NCF4, ITGB2, CYBB, CYBA, H2-AA, C3, FCGR1, IL1A, H2-DMB2, FCGR3, FCGR4, H2-DMB1, H2-OB, PTPN6, TLR4, H2-AB1, TLR2 |
| mmu05150:Staphylococcus aureus infection | 26 | C1QB, C1QA, CFH, PTAFR, ITGB2, C5AR1, ICAM1, C2, C3, C4B, H2-DMB2, C4A, C1S1, H2-DMB1, H2-OB, C3AR1, C1RA, H2-EB1, H2-AA, FCGR1, FCGR3, FCGR4, FCGR2B, CFB, C1QC, H2-AB1 |
| mmu04640:Hematopoietic cell lineage | 21 | H2-EB1, CSF3R, CSF1, H2-AA, FCGR1, CD24A, IL1A, H2-DMB2, ANPEP, H2-DMB1, IL3RA, H2-OB, CD9, CD37, CD36, ITGA5, CD14, CD22, CD33, CD44, H2-AB1 |
| mmu05323:Rheumatoid arthritis | 20 | CD86, CCL12, H2-EB1, CSF1, CD80, ITGB2, TCIRG1, H2-AA, CXCL5, ICAM1, IL1A, H2-DMB2, CTSL, H2-DMB1, CCL3, H2-OB, ACP5, TLR4, H2-AB1, TLR2 |
| mmu05164:Influenza A | 26 | CCL12, OAS1A, ICAM1, PYCARD, IFIH1, H2-DMB2, SOCS3, CASP8, H2-DMB1, CASP1, TNFSF10, H2-OB, NLRP3, IKBKE, H2-EB1, RSAD2, MX1, H2-AA, CXCL10, IL1A, OAS2, IRF7, FAS, TLR7, TLR4, H2-AB1 |
| mmu04060:Cytokine-cytokine receptor interaction | 33 | CCL12, CSF3R, CSF1, CXCR4, CSF2RB, IL2RG, CXCL5, CXCL16, CCL9, CCL6, CCL3, LEPR, TNFSF10, IL21R, CCL19, CCR5, CCR2, IL13RA1, CCL22, IL10RB, IL10RA, OSMR, TNFRSF1B, BMP5, TGFBR2, CSF2RB2, BMP4, CXCL10, IL1A, CLCF1, IL3RA, FAS, PF4 |
| mmu05332:Graft-versus-host disease | 15 | CD86, H2-EB1, CD80, H2-Q7, H2-M3, H2-K1, H2-Q4, H2-AA, IL1A, H2-DMB2, H2-DMB1, H2-OB, FAS, H2-D1, H2-AB1 |
| mmu04061:Viral protein interaction with cytokine and cytokine receptor | 18 | CCL12, CCL22, CSF1, IL10RB, IL10RA, CXCR4, TNFRSF1B, IL2RG, CXCL5, CXCL10, CCL9, CCL6, CCL3, TNFSF10, CCL19, CCR5, CCR2, PF4 |
| mmu05416:Viral myocarditis | 17 | CD86, H2-EB1, CD80, H2-Q7, H2-M3, H2-K1, ITGB2, H2-Q4, H2-AA, ICAM1, H2-DMB2, CASP8, H2-DMB1, H2-OB, RAC2, H2-D1, H2-AB1 |
| mmu04620:Toll-like receptor signaling pathway | 18 | CD86, TICAM2, CD80, TLR1, CXCL10, CASP8, IRF7, SPP1, CCL3, MAP3K8, IRF5, TLR7, LBP, TLR6, CD14, IKBKE, TLR4, TLR2 |
| mmu04612:Antigen processing and presentation | 17 | CD74, H2-EB1, H2-Q7, H2-M3, H2-K1, H2-Q4, TAP1, IFI30, H2-AA, CTSS, H2-DMB2, CTSL, H2-DMB1, H2-OB, B2M, H2-D1, H2-AB1 |
| mmu04380:Osteoclast differentiation | 20 | SPI1, NCF1, CSF1, NCF2, NCF4, CYBA, TREM2, LILRB4A, PIRB, TGFBR2, FCGR1, IL1A, SOCS3, FCGR3, TYROBP, FCGR4, BTK, ACP5, LCP2, FCGR2B |
| mmu04940:Type I diabetes mellitus | 15 | CD86, H2-EB1, CD80, H2-Q7, H2-M3, H2-K1, H2-Q4, H2-AA, IL1A, H2-DMB2, H2-DMB1, H2-OB, FAS, H2-D1, H2-AB1 |
| mmu05417:Lipid and atherosclerosis | 26 | CCL12, NCF1, NCF2, NCF4, ICAM1, PYCARD, CASP8, CASP1, CCL3, TNFSF10, NLRP3, CD36, LBP, CD14, IKBKE, ABCA1, TICAM2, CYBB, CYBA, VAV1, IRF7, FAS, TLR6, TLR4, TLR2, NFE2L2 |
| mmu05330:Allograft rejection | 14 | CD86, H2-EB1, CD80, H2-Q7, H2-M3, H2-K1, H2-Q4, H2-AA, H2-DMB2, H2-DMB1, H2-OB, FAS, H2-D1, H2-AB1 |
| mmu05169:Epstein-Barr virus infection | 26 | H2-M3, H2-Q7, H2-K1, H2-Q4, OAS1A, ICAM1, H2-DMB2, CASP8, H2-DMB1, H2-OB, IKBKE, B2M, H2-EB1, TAP1, ISG15, H2-AA, CXCL10, OAS2, BTK, IRF7, FAS, VIM, CD44, H2-D1, TLR2, H2-AB1 |
| mmu05171:Coronavirus disease - COVID-19 | 27 | C1QB, C1QA, CCL12, C5AR1, OAS1A, F13A1, C2, IFIH1, C3, C4B, C4A, C1S1, CASP1, C3AR1, NLRP3, IKBKE, C1RA, MX1, CYBB, ISG15, CXCL10, OAS2, TLR7, TLR4, CFB, TLR2, C1QC |
| mmu04621:NOD-like receptor signaling pathway | 24 | GSDMD, GBP5, CCL12, IFI204, CYBB, OAS1A, CYBA, PYCARD, NAIP2, NAIP5, AIM2, CASP12, CASP8, NAIP6, OAS2, CASP4, CASP1, IRF7, TXNIP, NLRP3, GBP2, IKBKE, TLR4, GBP3 |
| mmu05320:Autoimmune thyroid disease | 14 | CD86, H2-EB1, CD80, H2-Q7, H2-M3, H2-K1, H2-Q4, H2-AA, H2-DMB2, H2-DMB1, H2-OB, FAS, H2-D1, H2-AB1 |
| mmu05322:Systemic lupus erythematosus | 19 | C1QB, CD86, C1QA, H2-EB1, C1RA, CD80, H2-AA, C2, C3, C4B, FCGR1, C4A, H2-DMB2, C1S1, FCGR4, H2-DMB1, H2-OB, C1QC, H2-AB1 |
| mmu04512:ECM-receptor interaction | 14 | FN1, THBS2, THBS1, THBS4, COL1A1, COL1A2, COL6A2, COL6A1, SPP1, COL4A5, COL6A5, CD36, ITGA5, CD44 |
| mmu04662:B cell receptor signaling pathway | 13 | IFITM1, CD72, DAPP1, LILRB4A, PIRB, VAV1, BTK, RAC2, PTPN6, PIK3AP1, FCGR2B, CD22, CARD11 |
| mmu04974:Protein digestion and absorption | 15 | KCNK5, COL18A1, COL14A1, COL12A1, COL1A1, COL3A1, COL1A2, SLC7A7, COL5A1, COL6A2, COL5A2, COL6A1, COL8A2, COL4A5, COL6A5 |
| mmu05132:Salmonella infection | 24 | GSDMD, ANXA2, AHNAK, ARPC1B, FHOD1, PIK3CG, PYCARD, NAIP2, NAIP5, PTPRC, CASP8, CYTH4, NAIP6, CASP4, CASP1, TNFSF10, NLRP3, NCKAP1L, TLR6, FLNC, CD14, RAB7B, TLR4, TLR2 |
| mmu05134:Legionellosis | 11 | PYCARD, C3, NAIP2, NAIP5, CASP8, NAIP6, ITGB2, CASP1, CD14, TLR4, TLR2 |
| mmu04062:Chemokine signaling pathway | 20 | CCL12, CCL22, NCF1, CXCR4, PIK3CG, VAV1, CXCL5, CXCL16, CXCL10, HCK, CCL9, GNGT2, CCL6, CCL3, RAC2, CCL19, DOCK2, CCR5, CCR2, PF4 |
| mmu05321:Inflammatory bowel disease | 11 | H2-DMB2, IL1A, H2-EB1, H2-DMB1, IL21R, H2-OB, IL2RG, H2-AA, TLR4, H2-AB1, TLR2 |
| **mmu04210:Apoptosis** | **16** | **PARP3, CTSZ, CSF2RB, TRAF1, CTSS, CSF2RB2, CASP12, CASP8, CTSL, IL3RA, TNFSF10, CTSH, FAS, CTSD, BCL2A1B, CTSC** |
| mmu04672:Intestinal immune network for IgA production | 9 | CD86, H2-DMB2, H2-EB1, CD80, H2-DMB1, H2-OB, CXCR4, H2-AA, H2-AB1 |
