# Supplementary material for: Very early environmental enrichment protects against apoptosis and improves functional recovery from hypoxic–ischemic brain injury
Source: Front Mol Neurosci. 2023 Feb 7;15:1019173. doi: 10.3389/fnmol.2022.1019173 (PMC9942523; doi:10.3389/fnmol.2022.1019173)

Drp1 (Figure 4)

Very early exposure

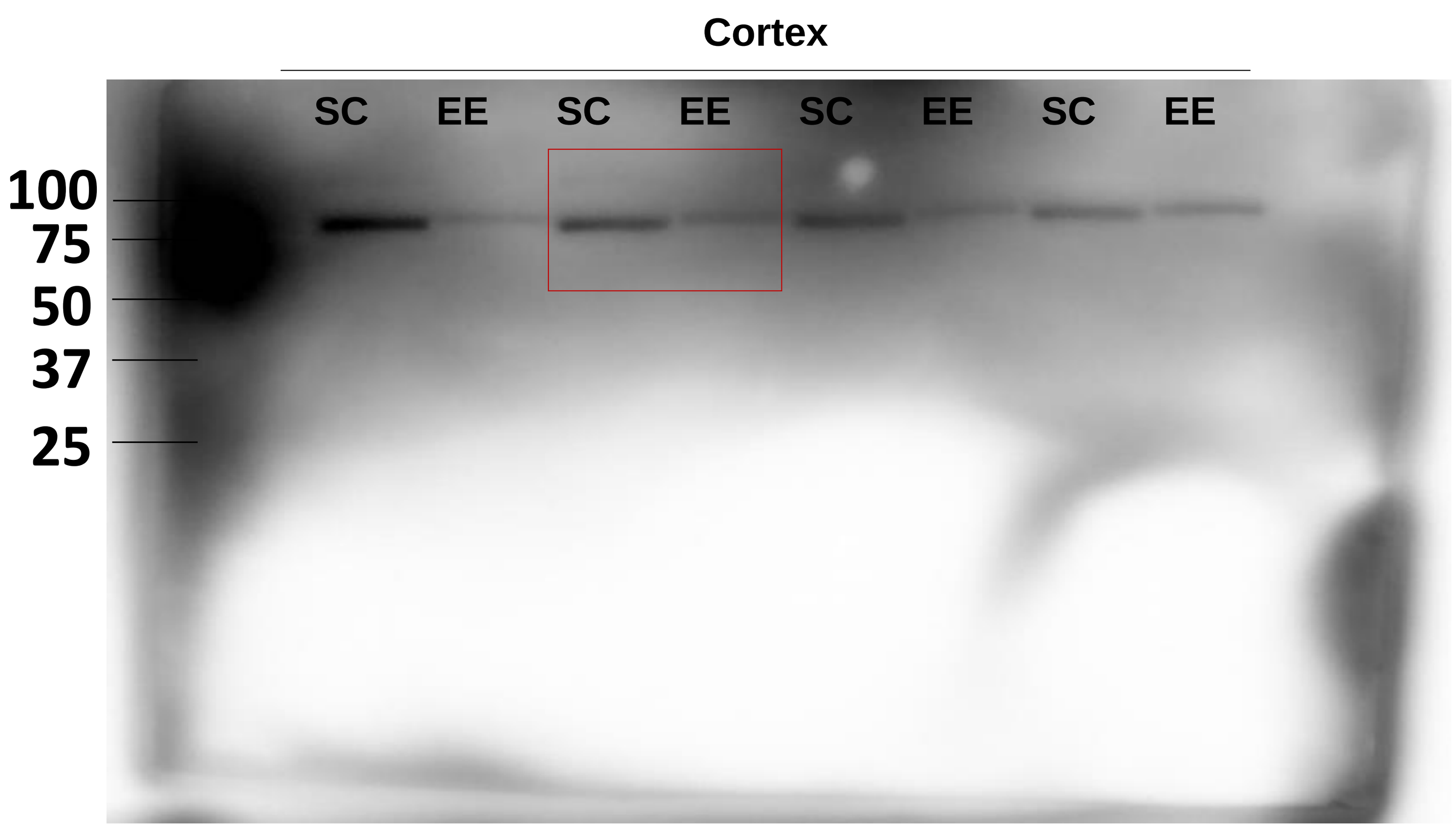

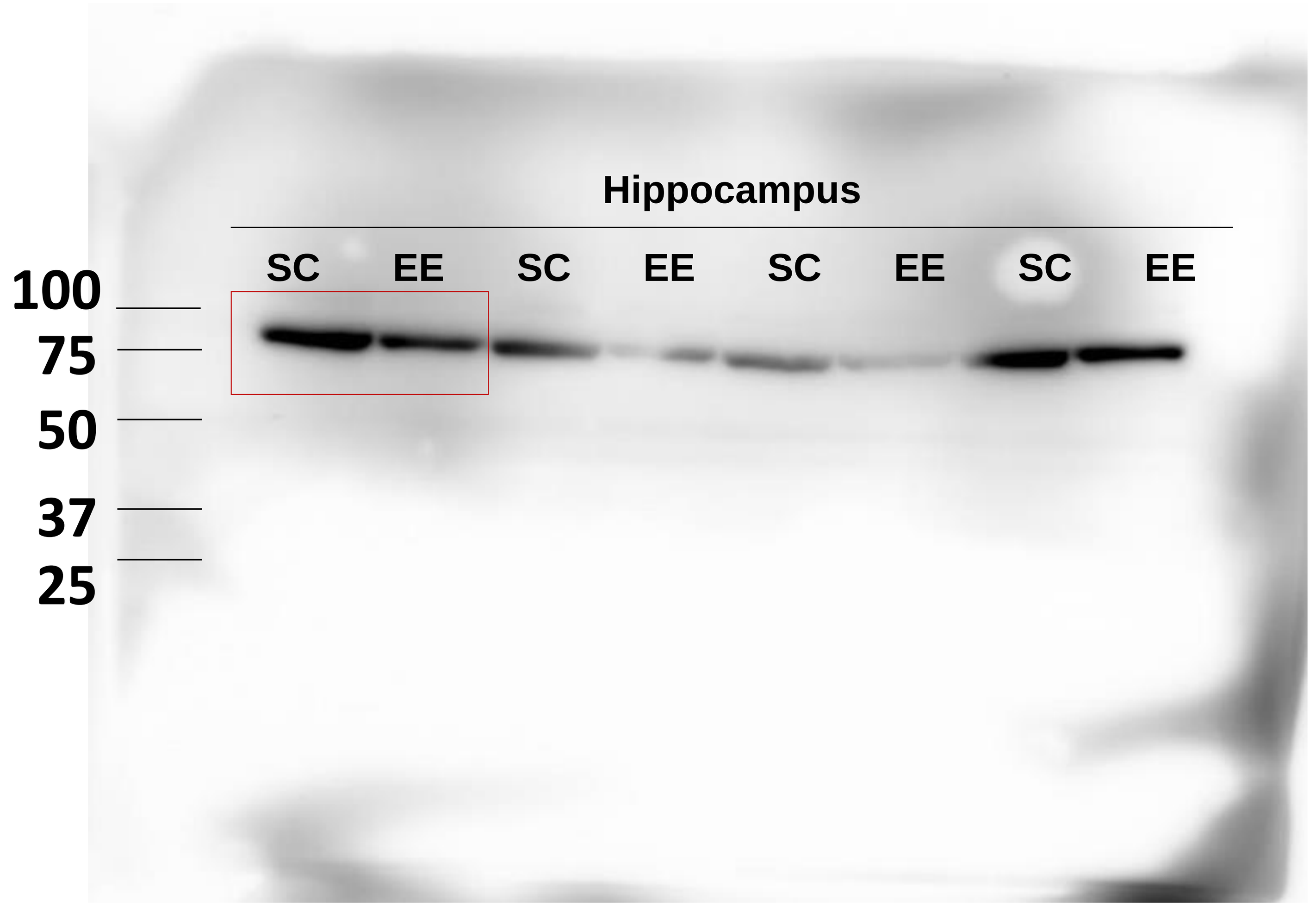

Delayed exposure

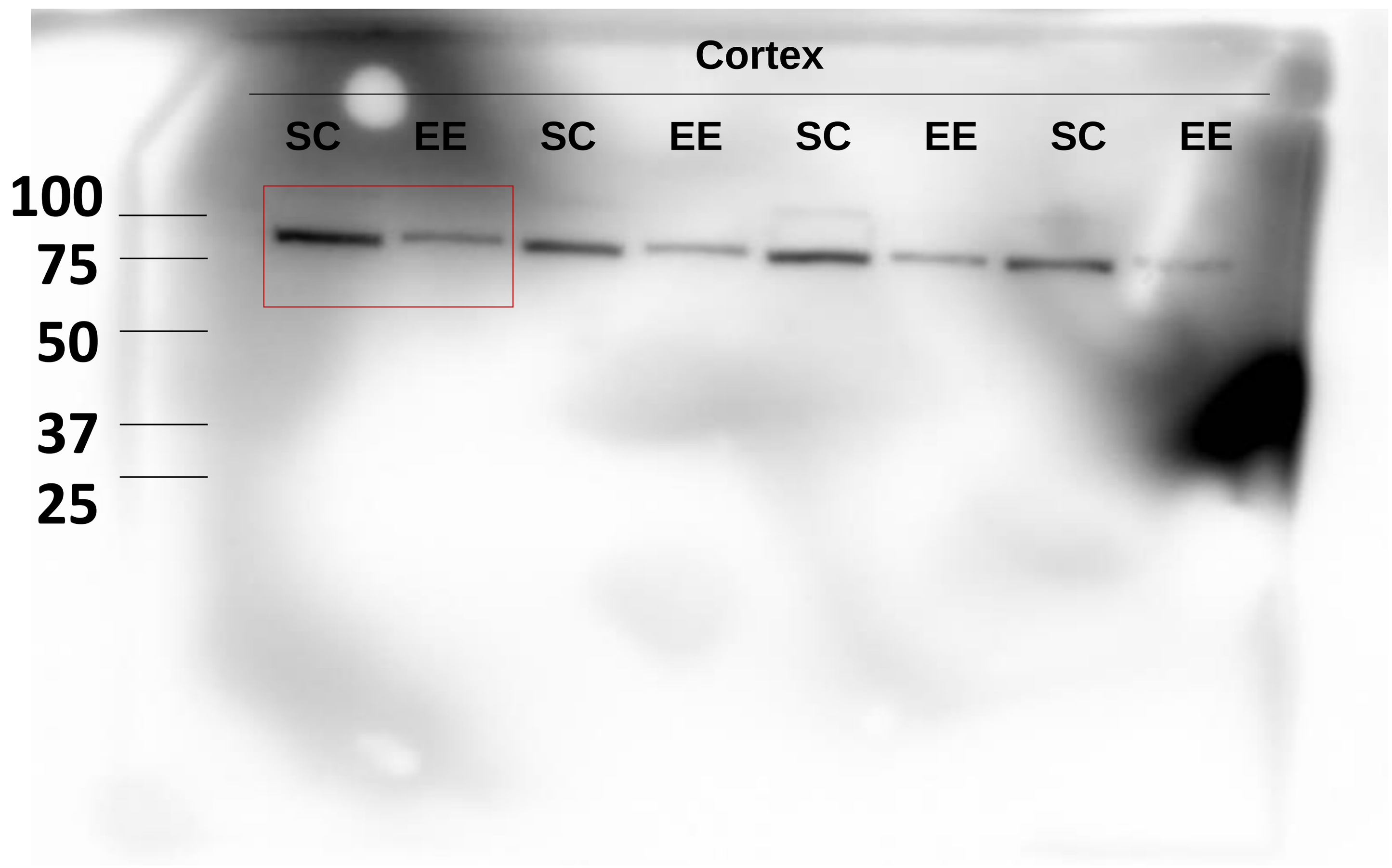

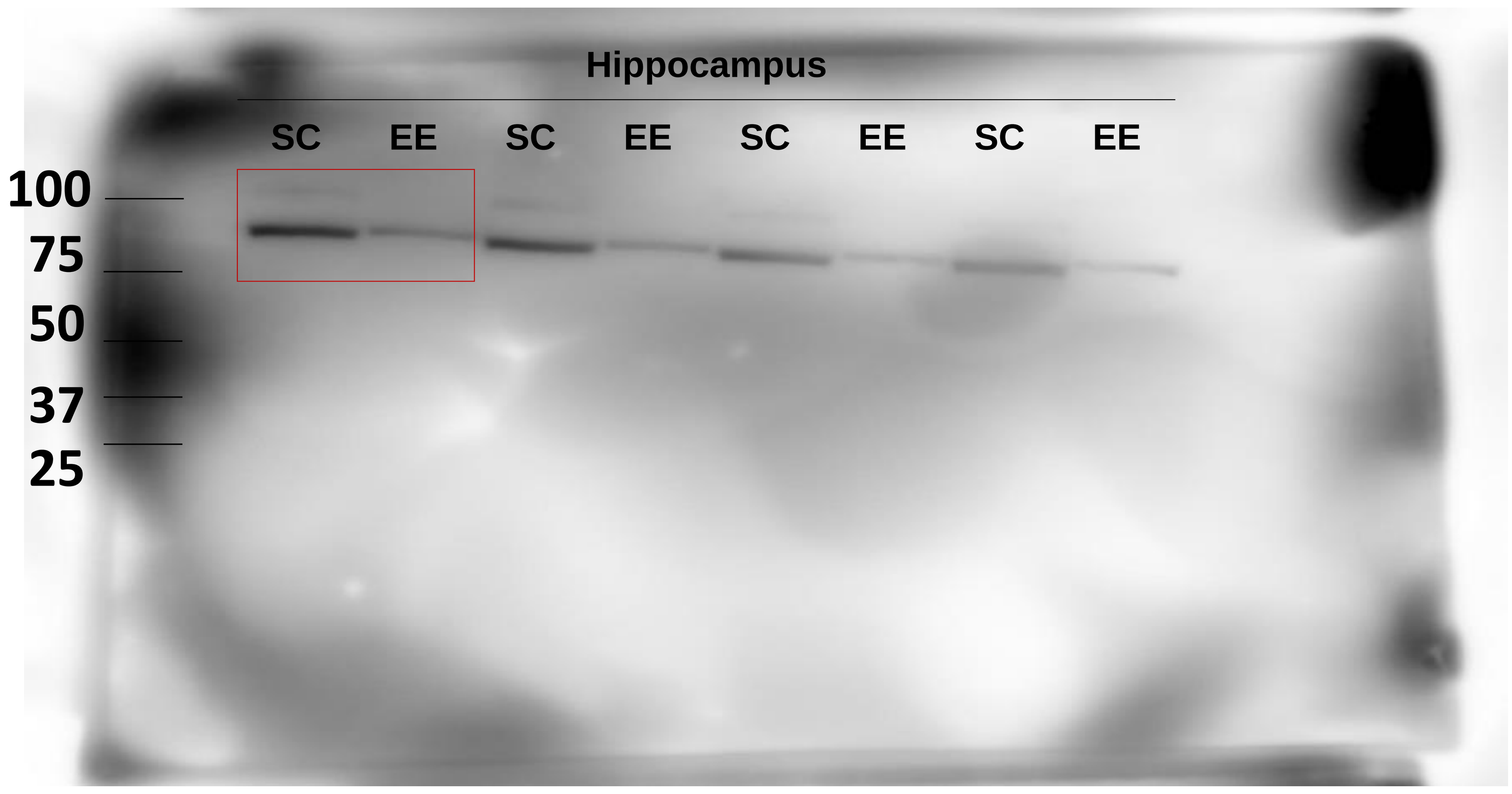

Bax (Figure 4)

Very early exposure

Delayed exposure

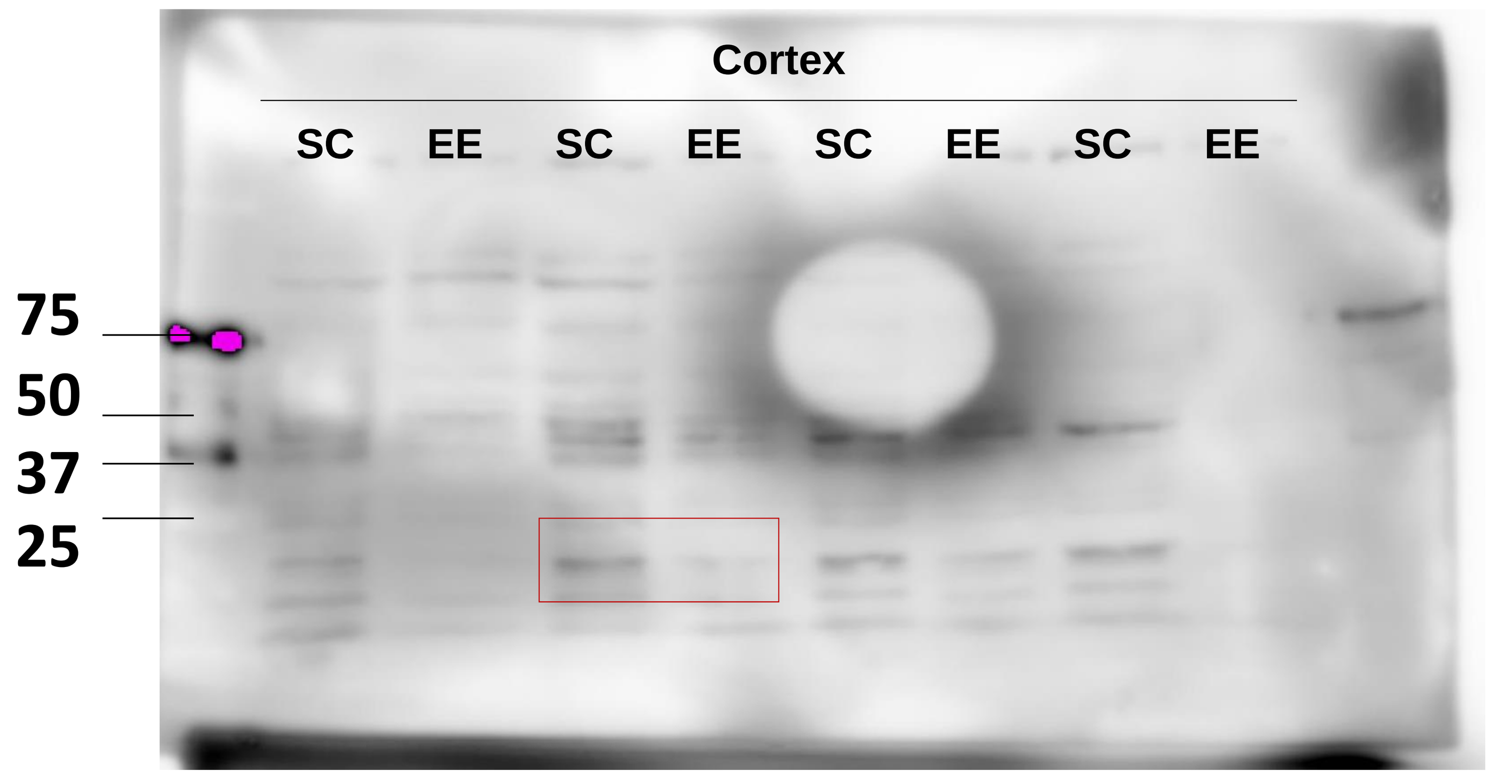

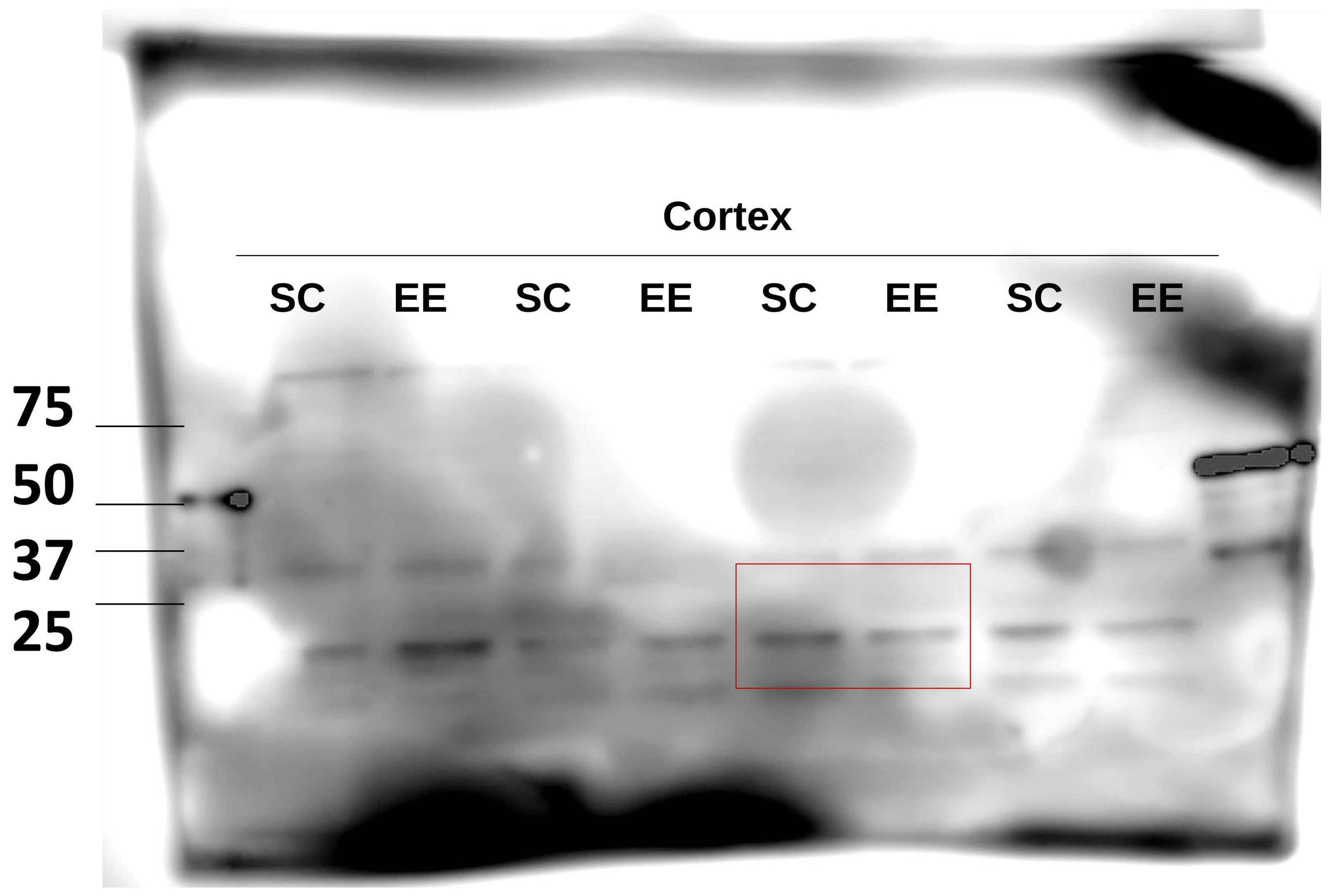

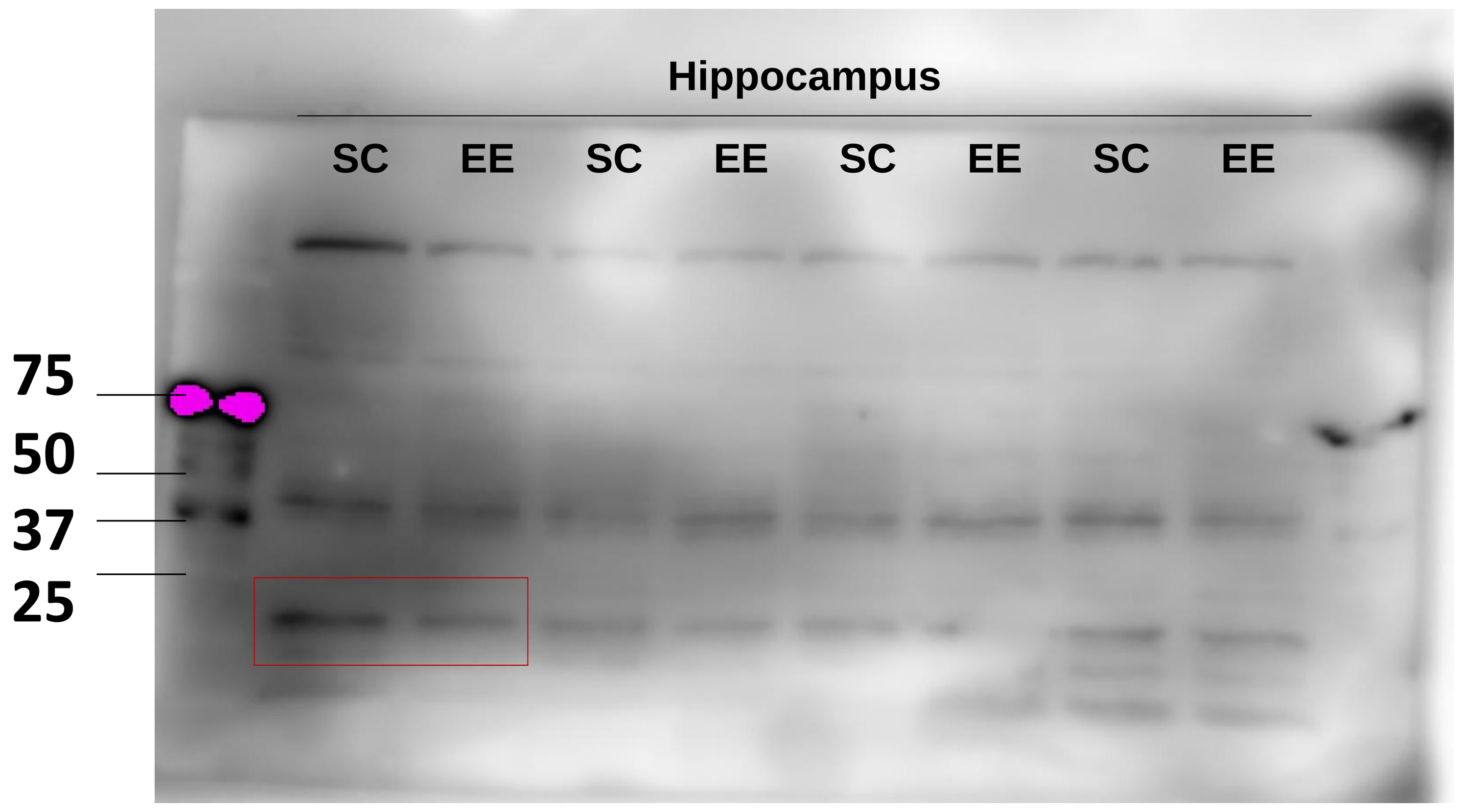

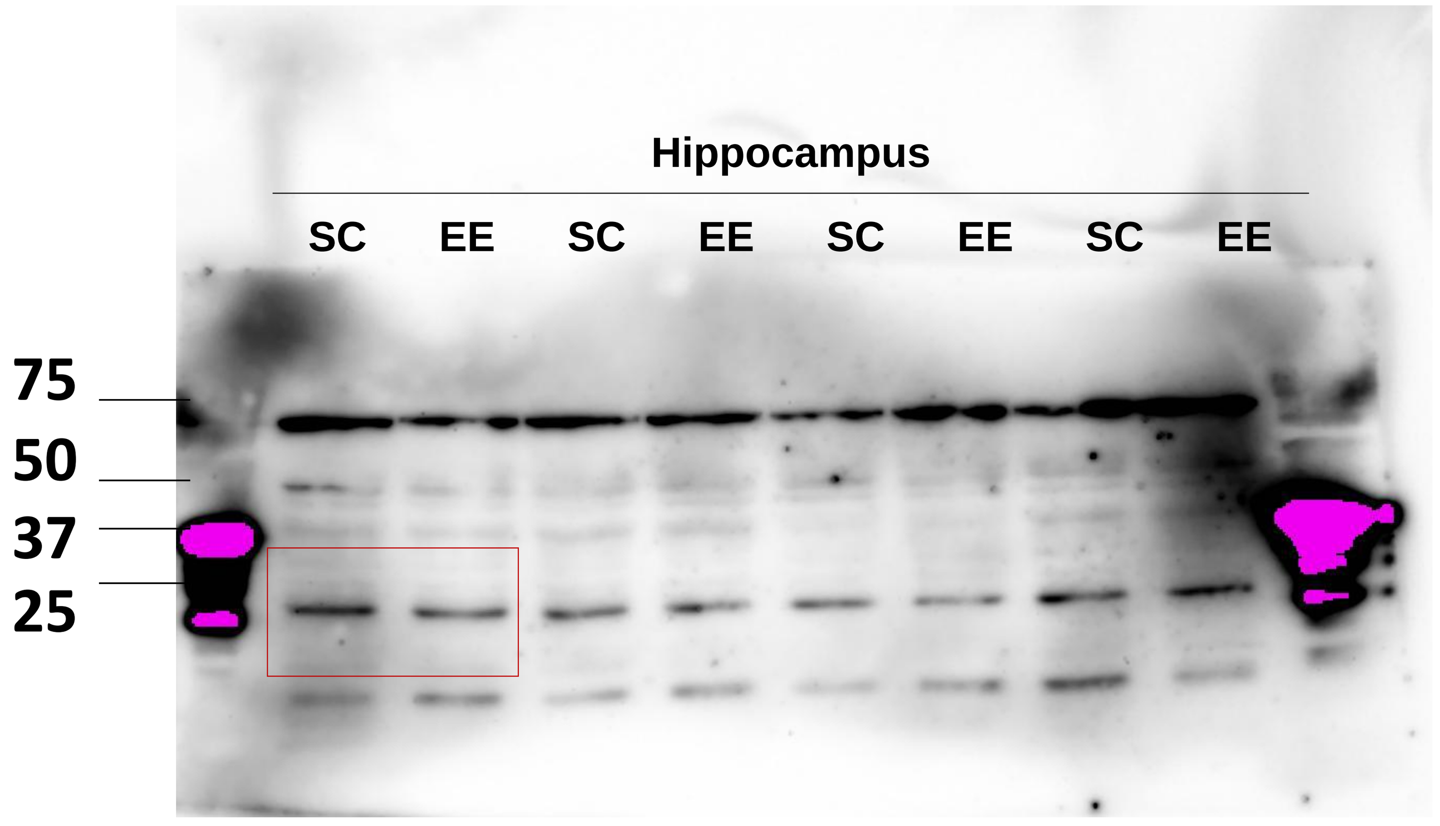

Bcl-2 (Figure 4)

Very early exposure

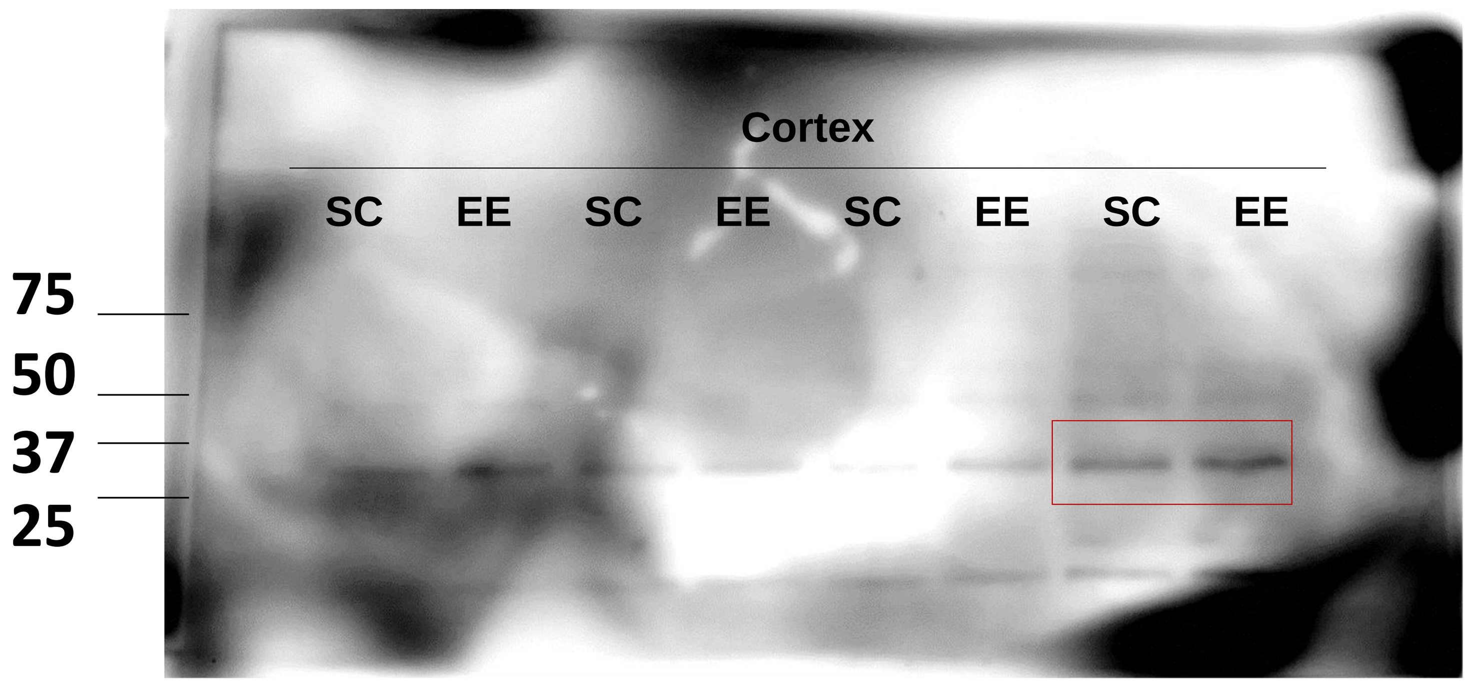

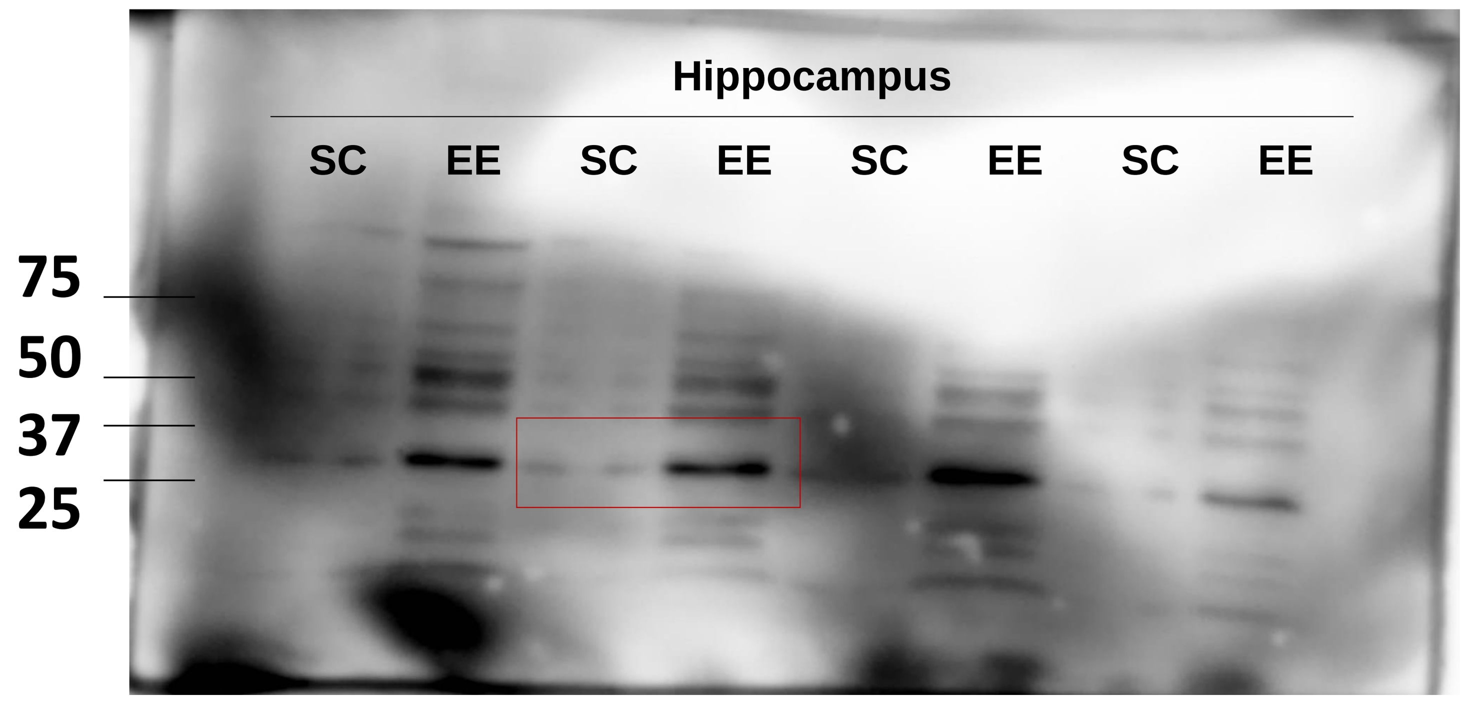

Delayed exposure

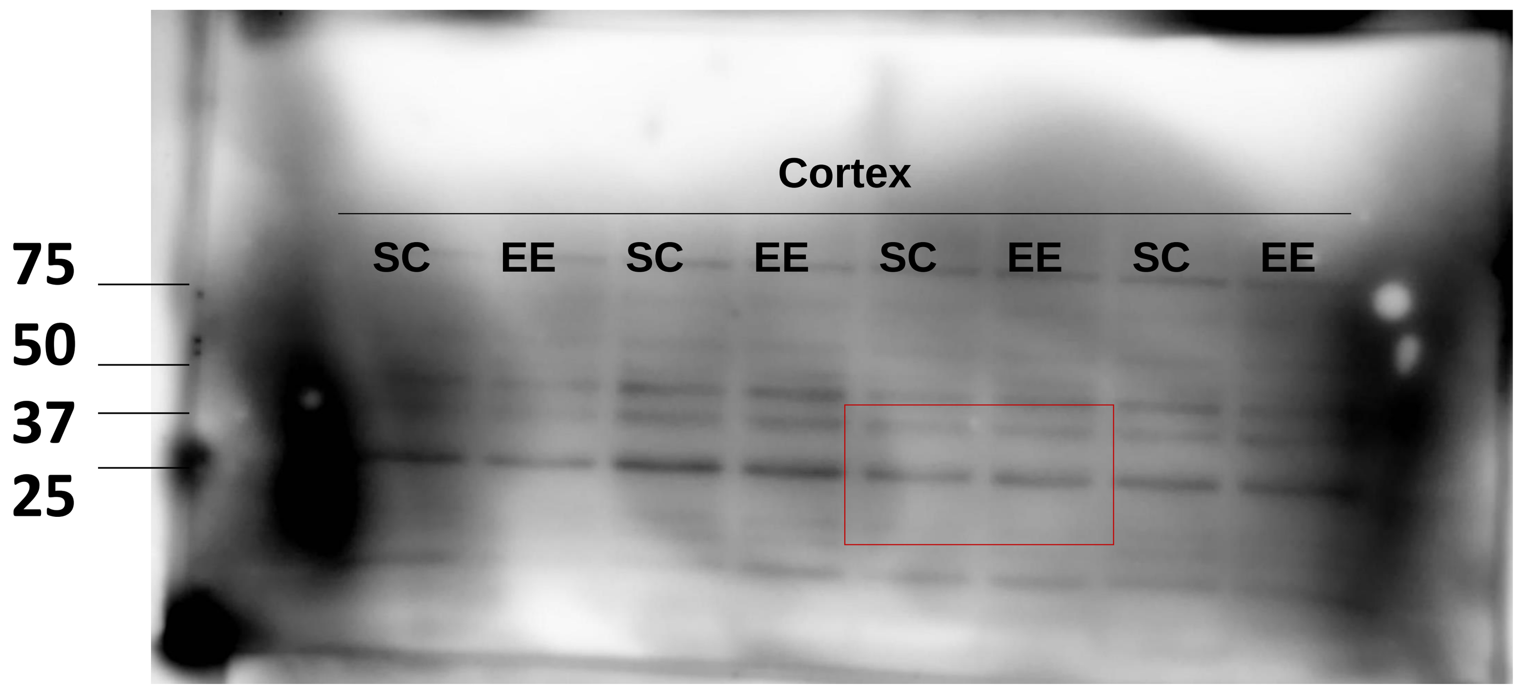

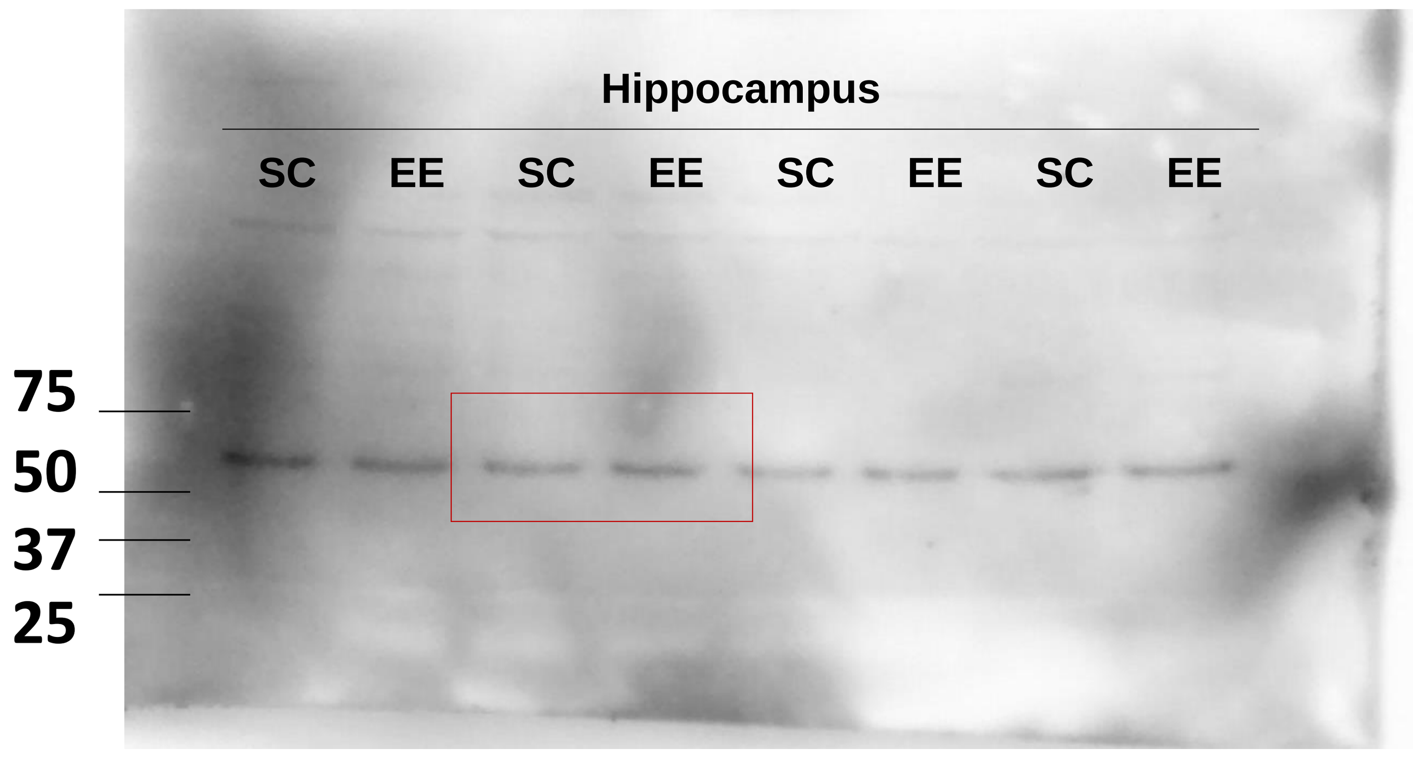

Cleaved Caspase-3 (Figure 4)

Very early exposure

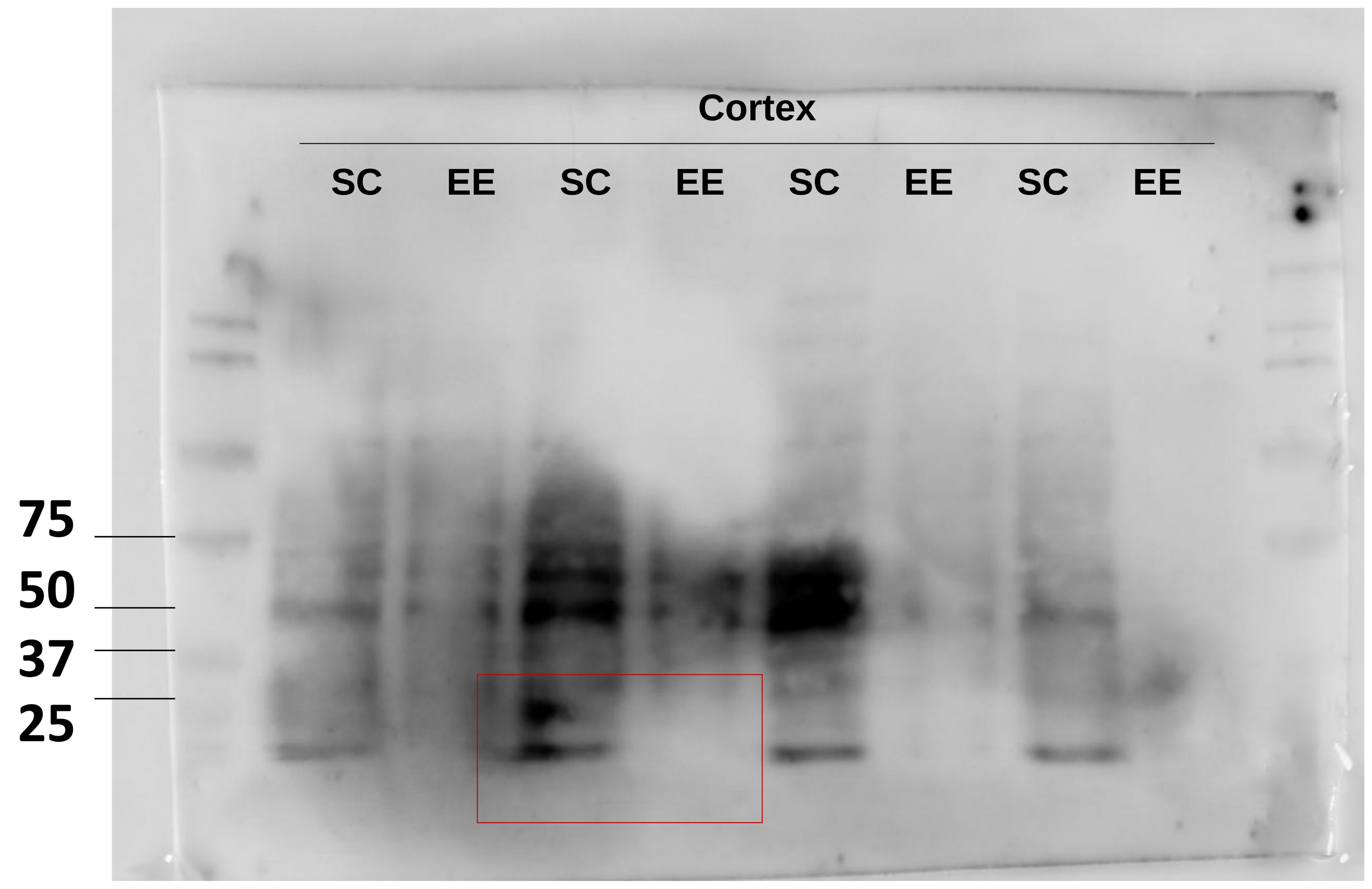

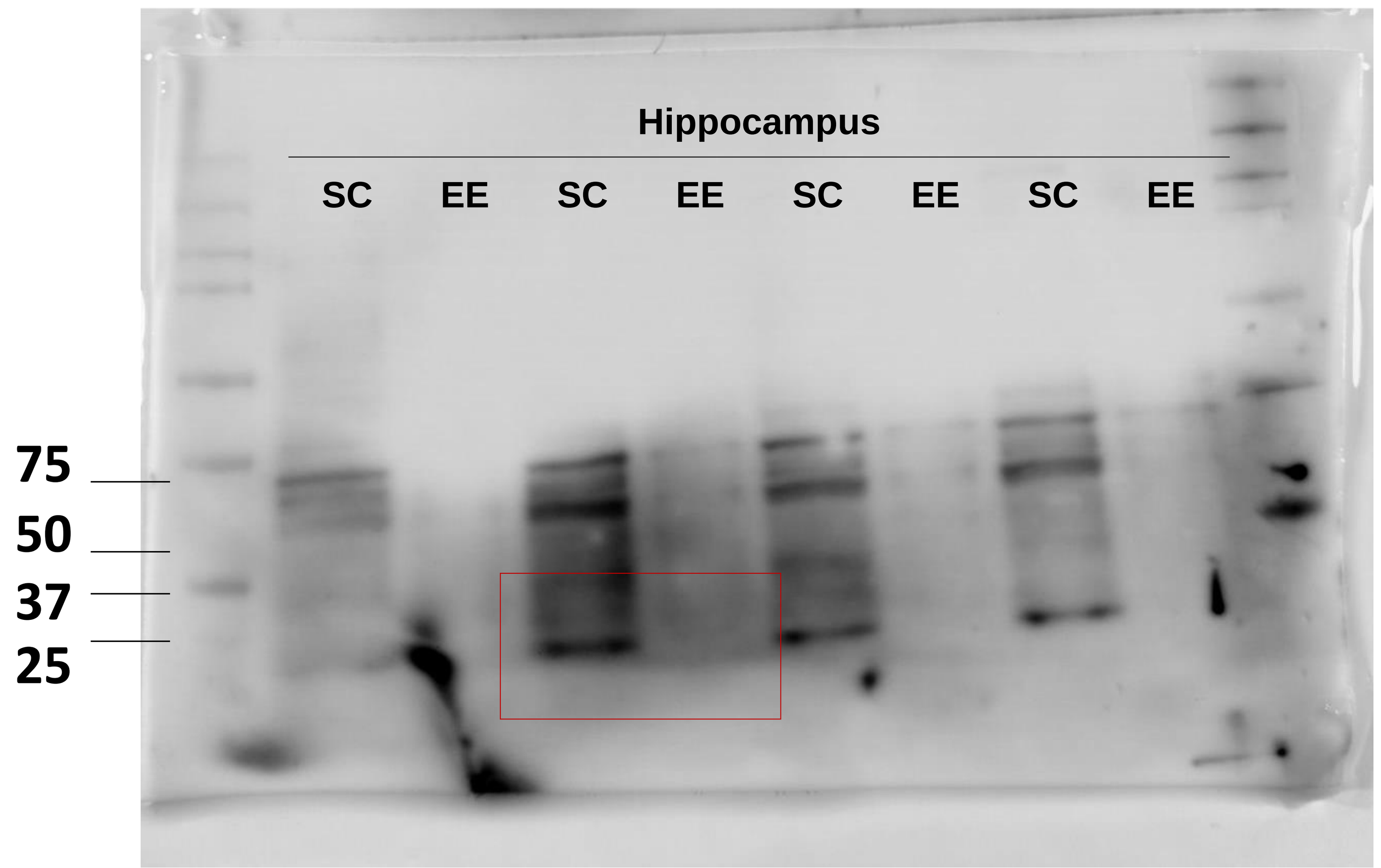

Delayed exposure

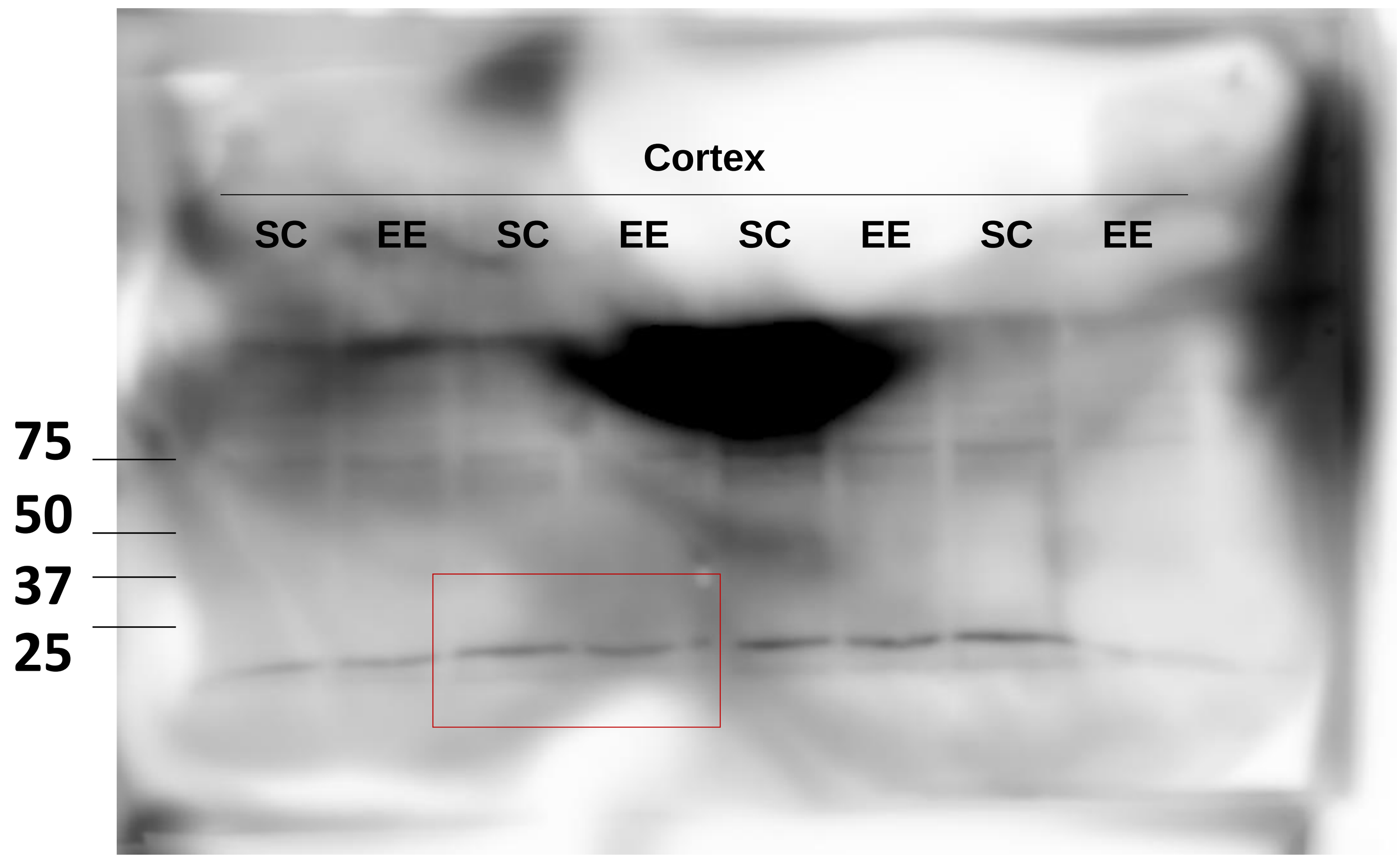

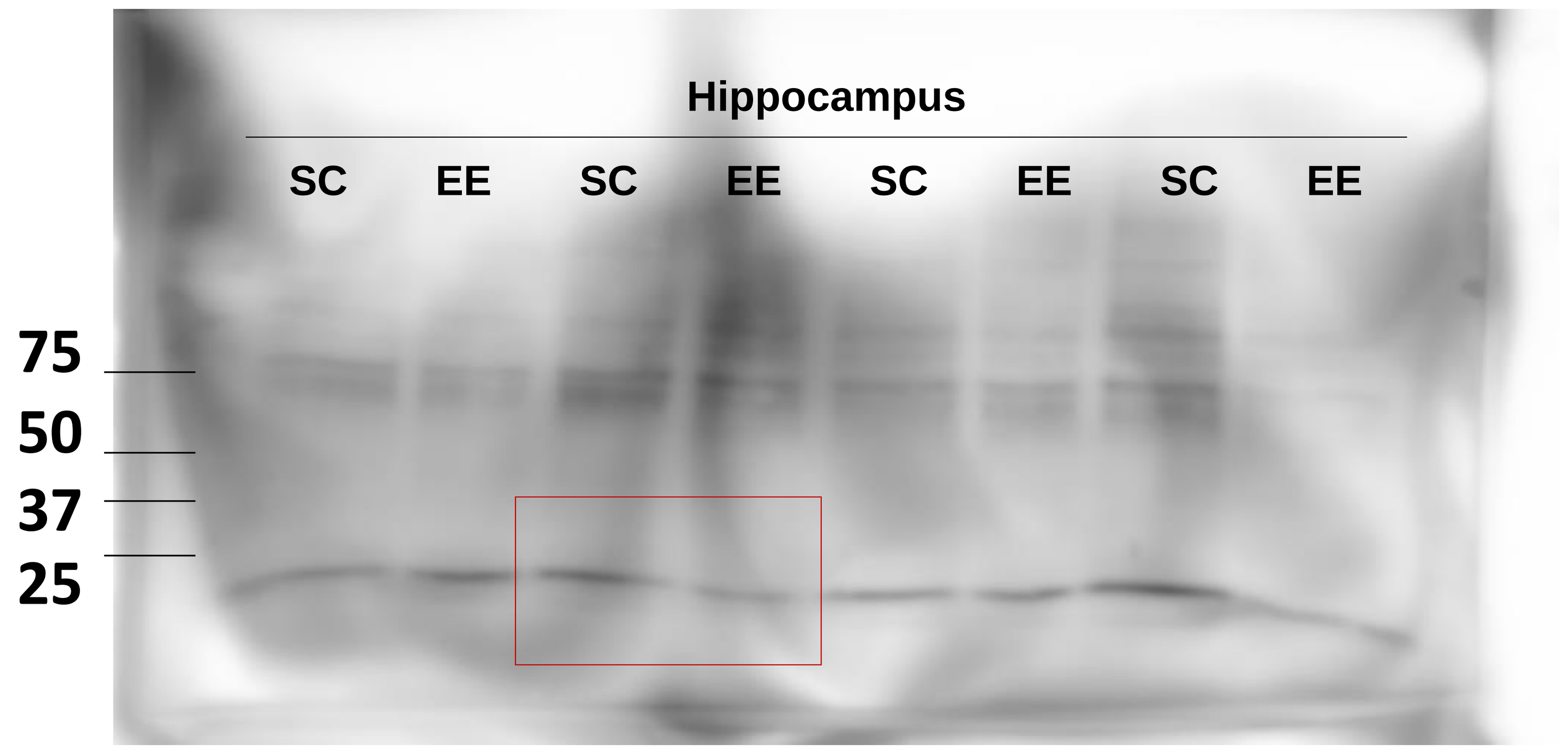

Caspase-3 (Figure 4)

Very early exposure

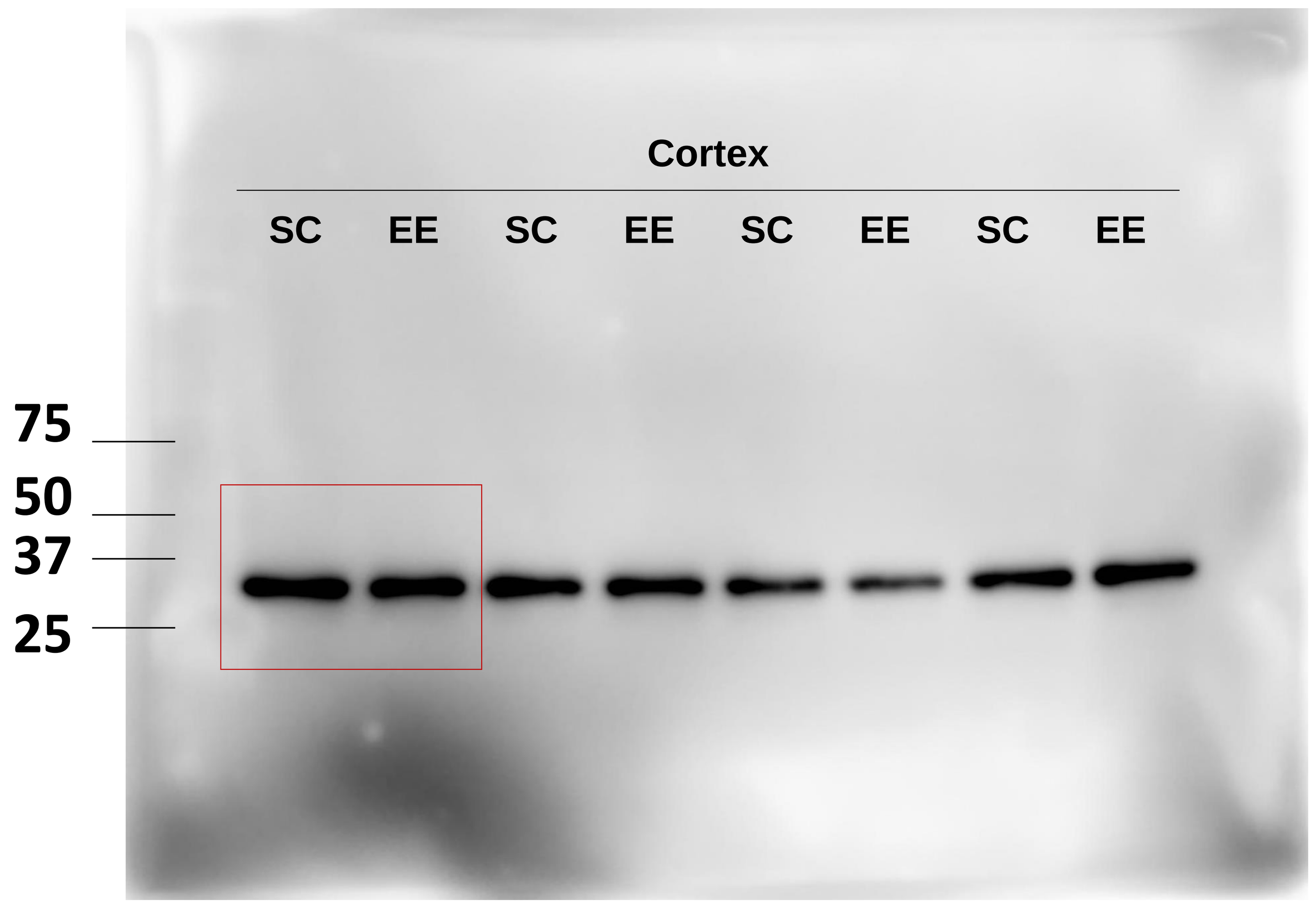

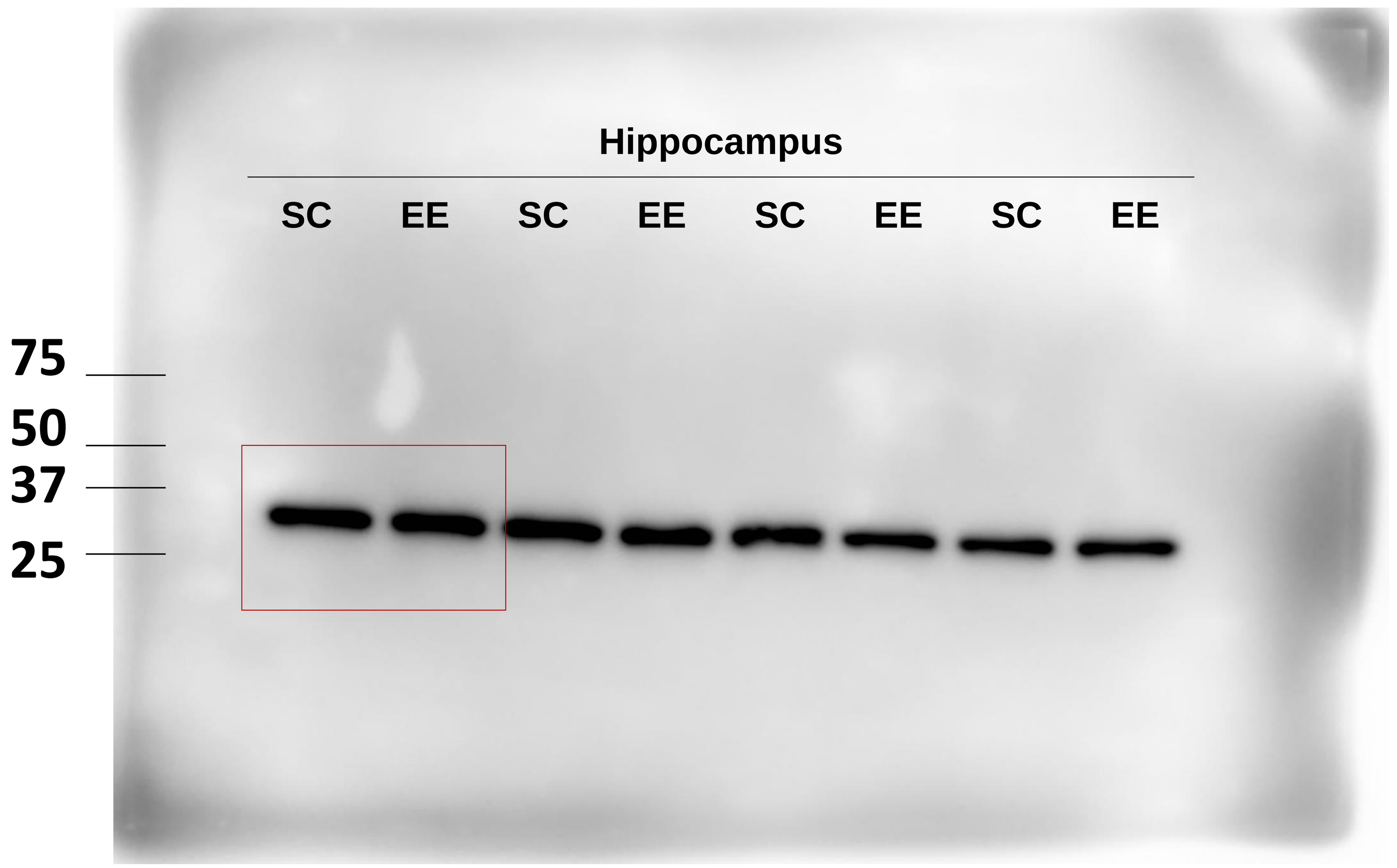

Delayed exposure

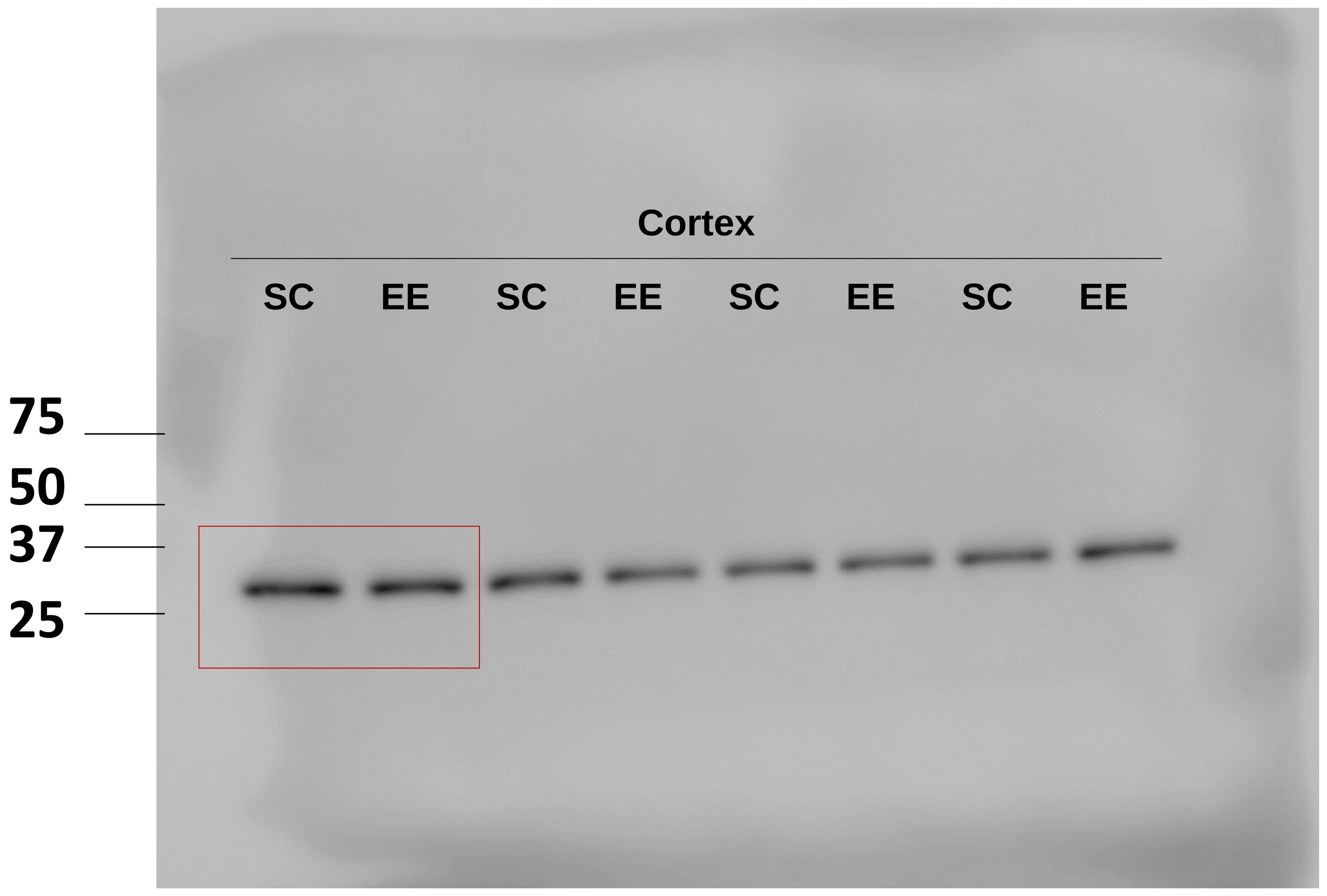

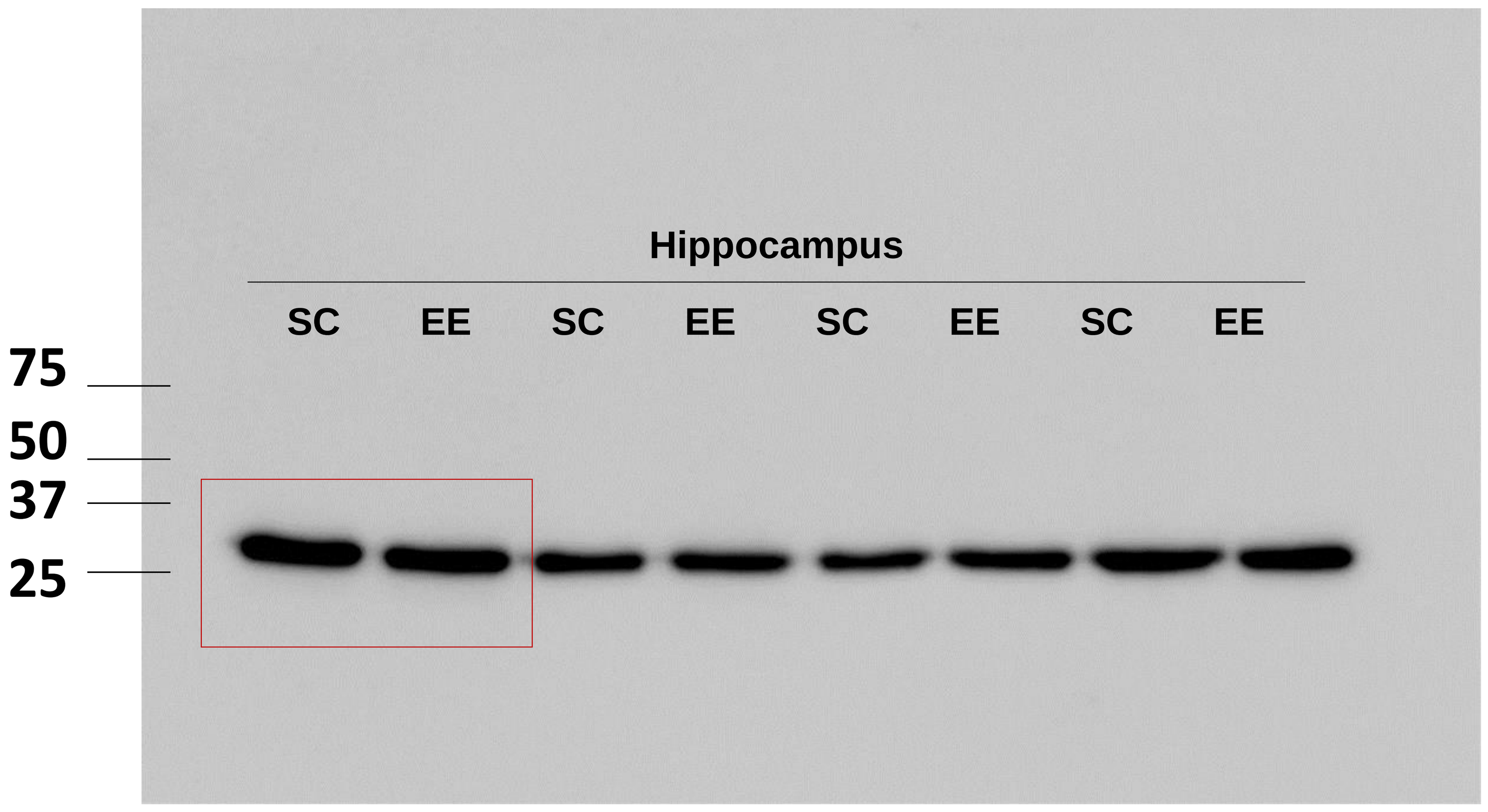

Actin (Figure 4)

Very early exposure

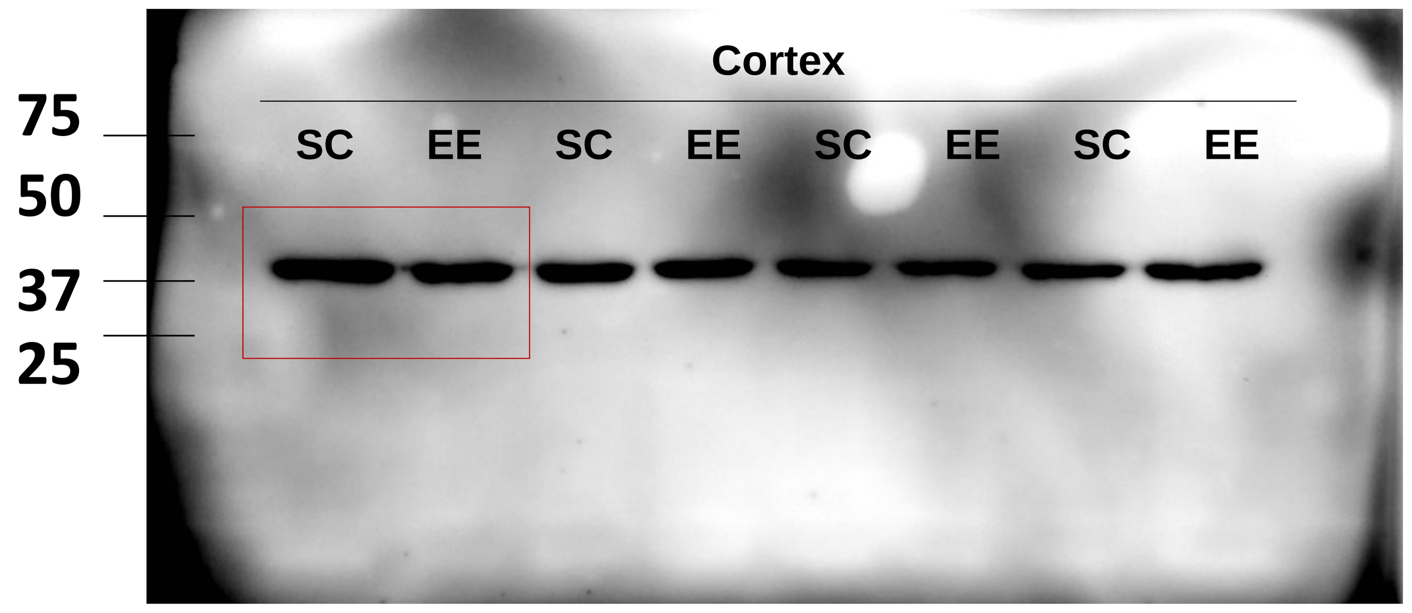

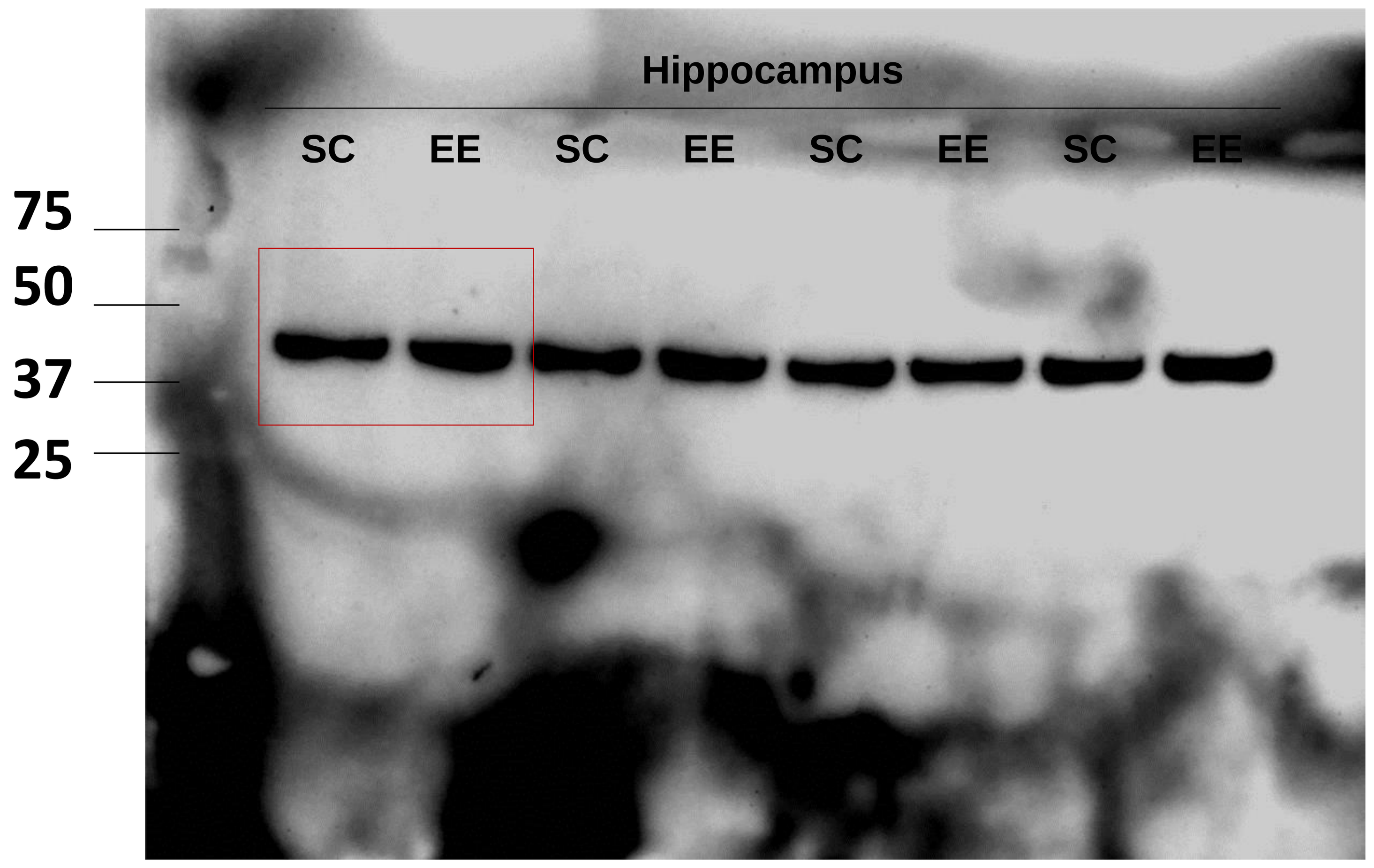

Delayed exposure

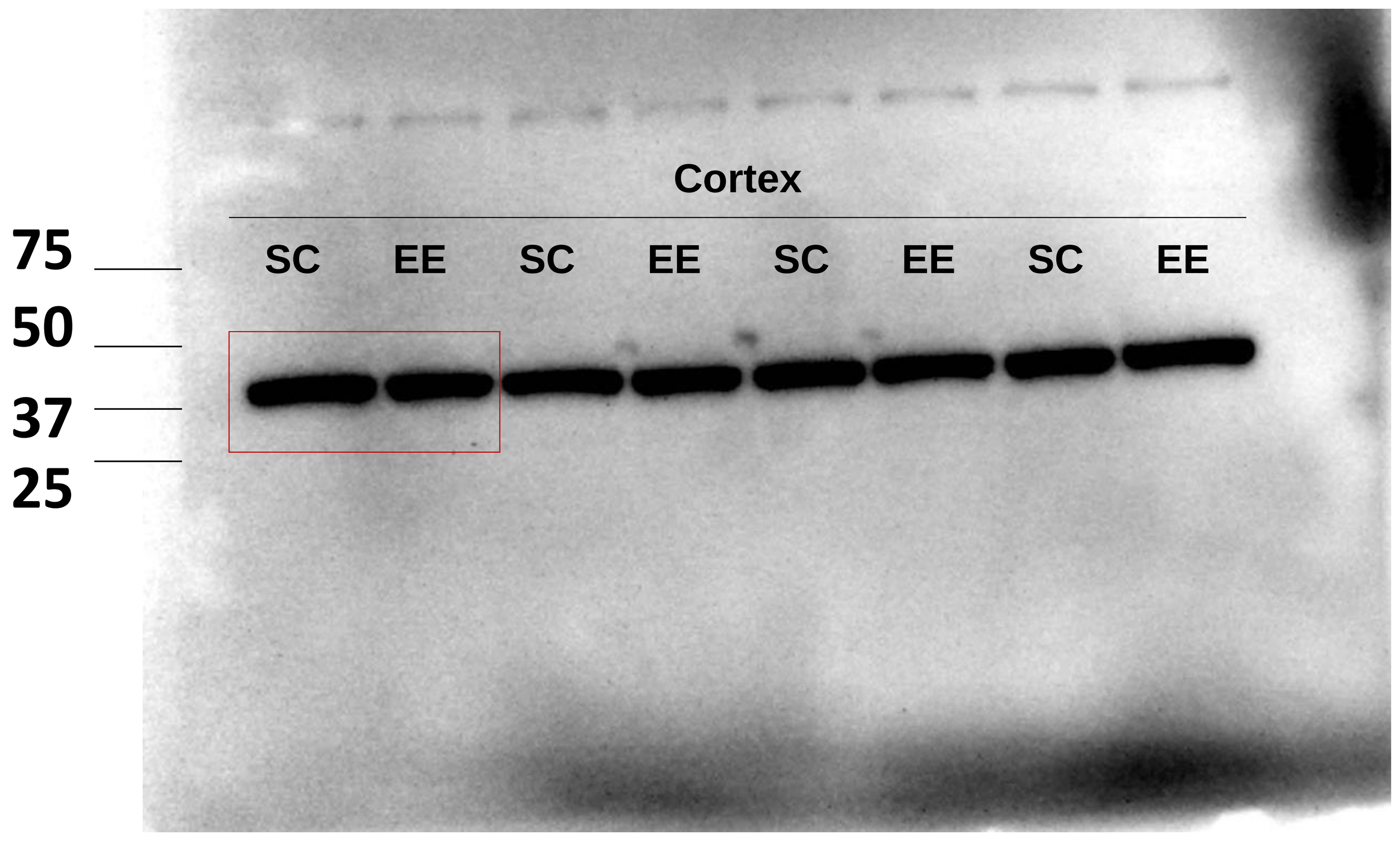

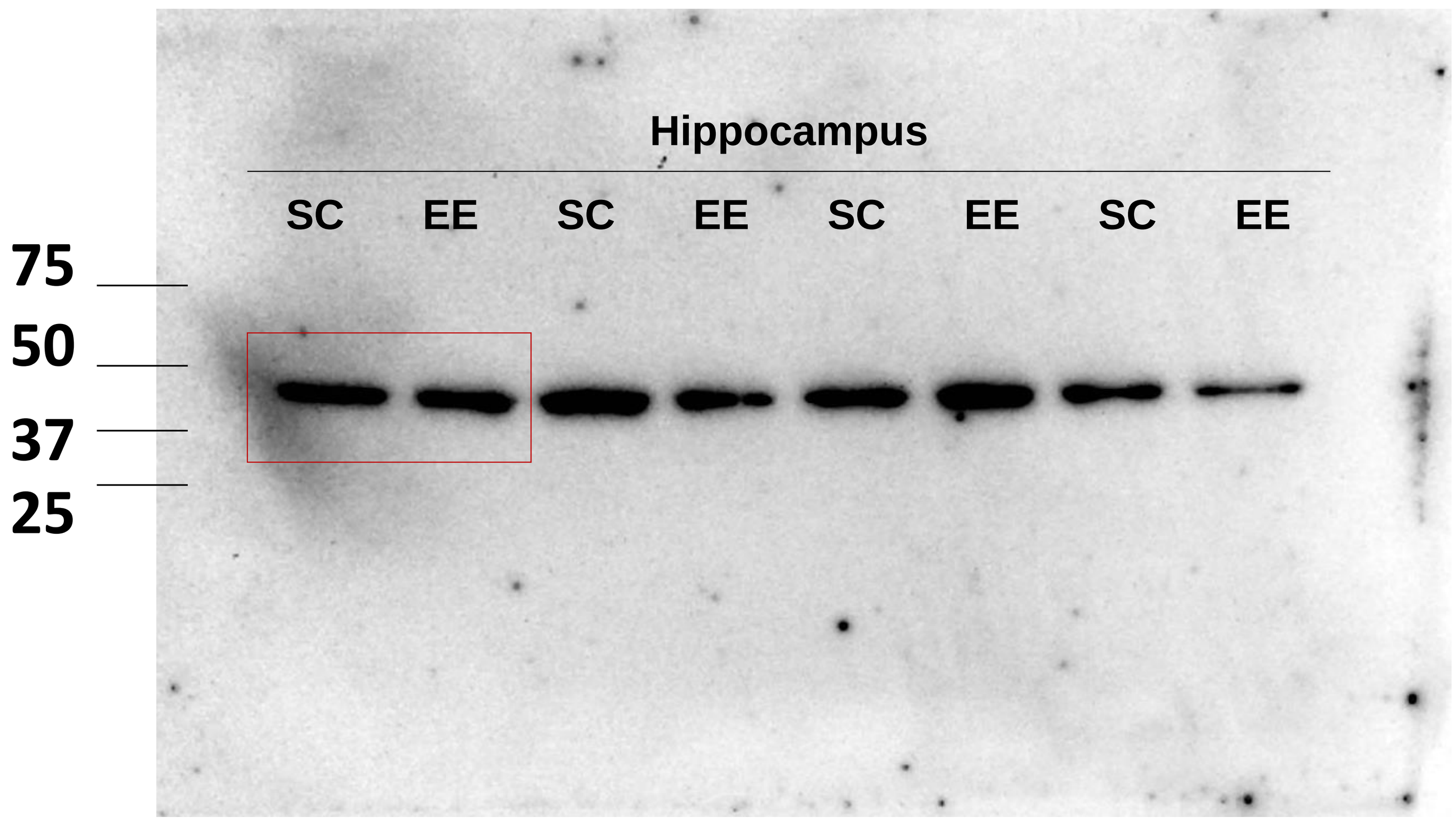

iNOS (Figure 5)

Very early exposure

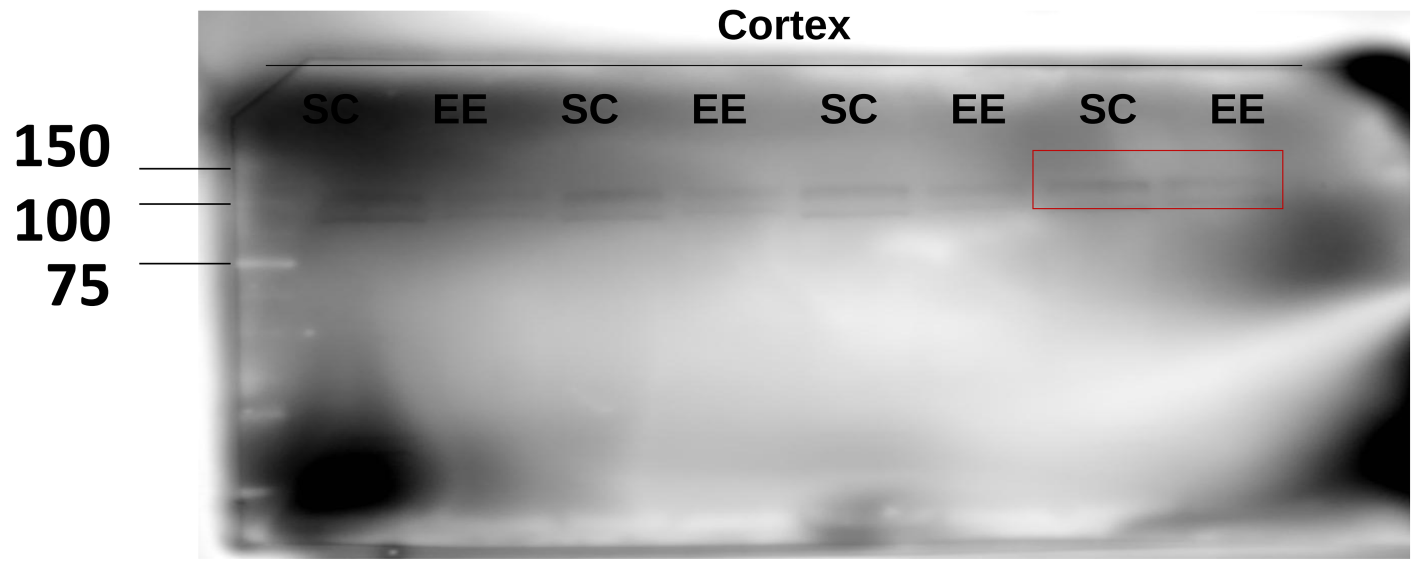

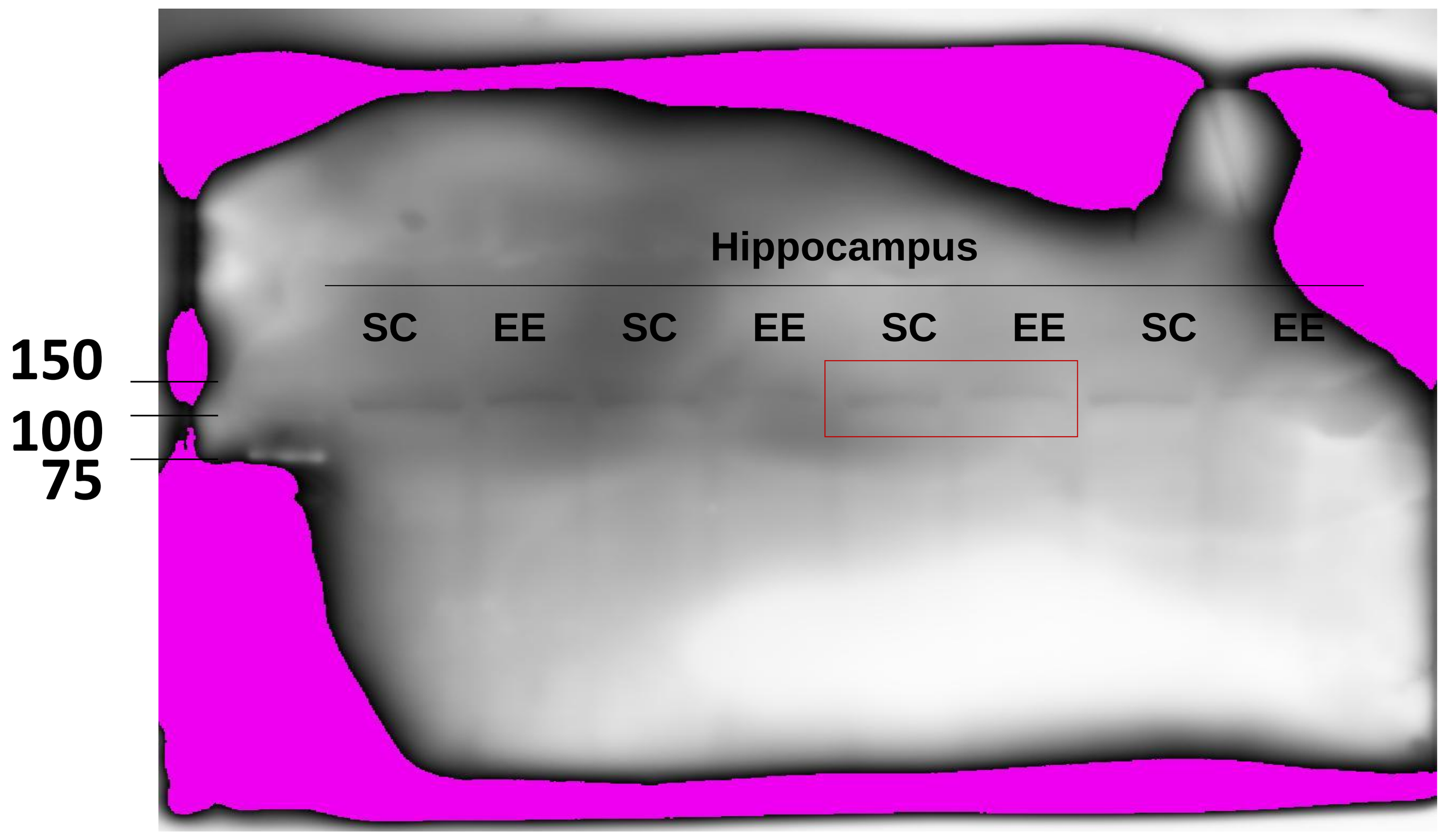

Delayed exposure

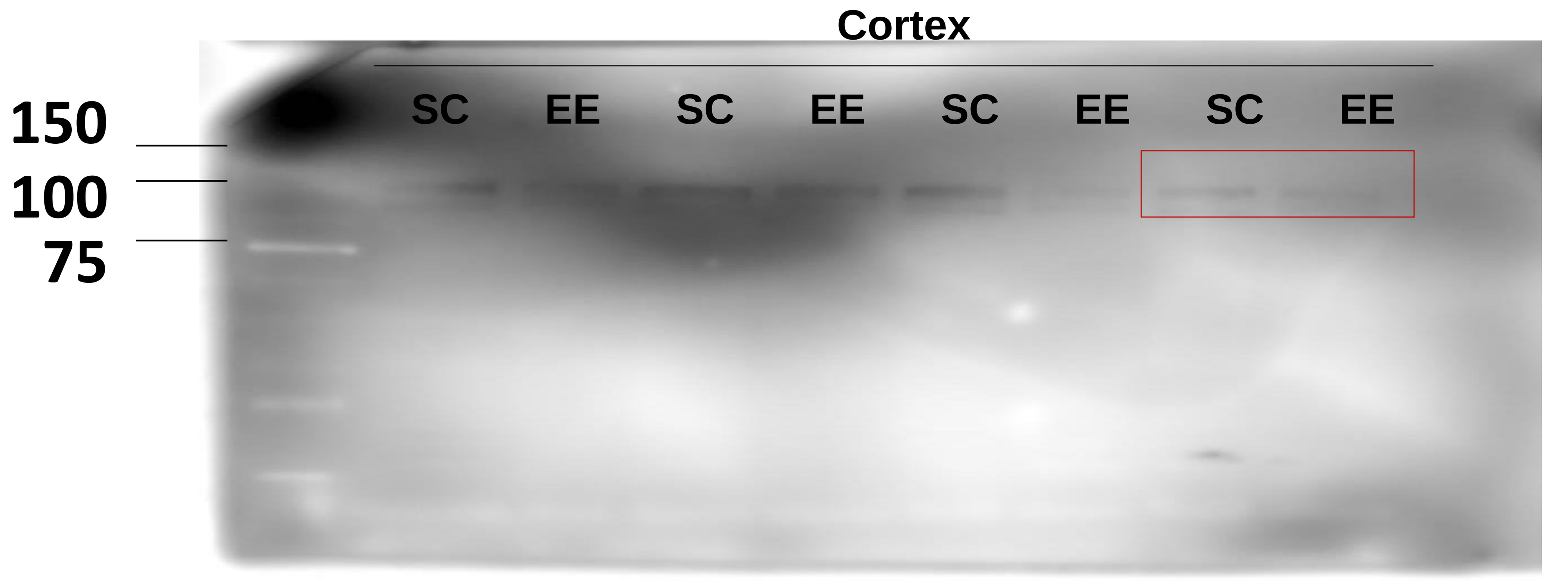

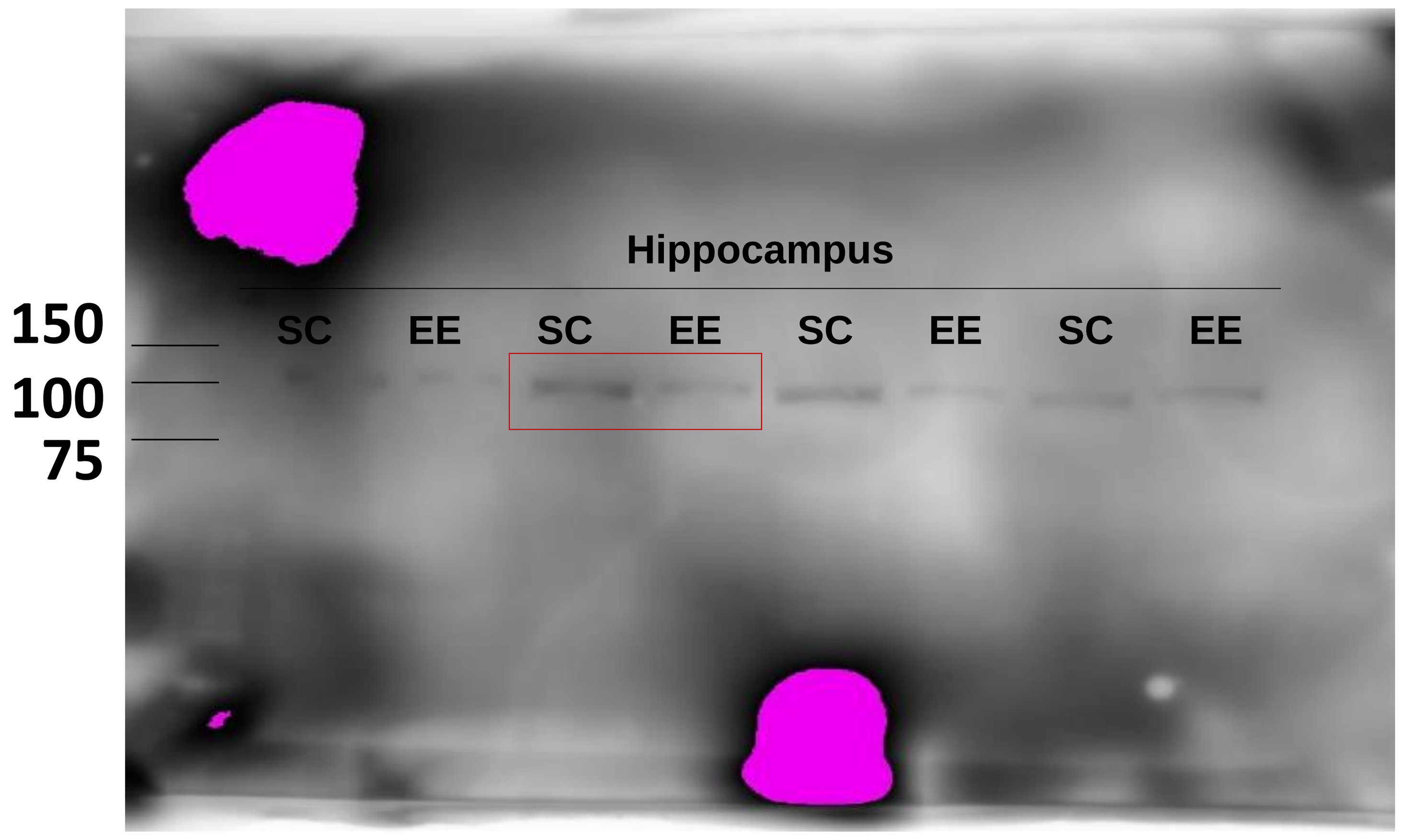

Cox2 (Figure 5)

Very early exposure

Delayed exposure

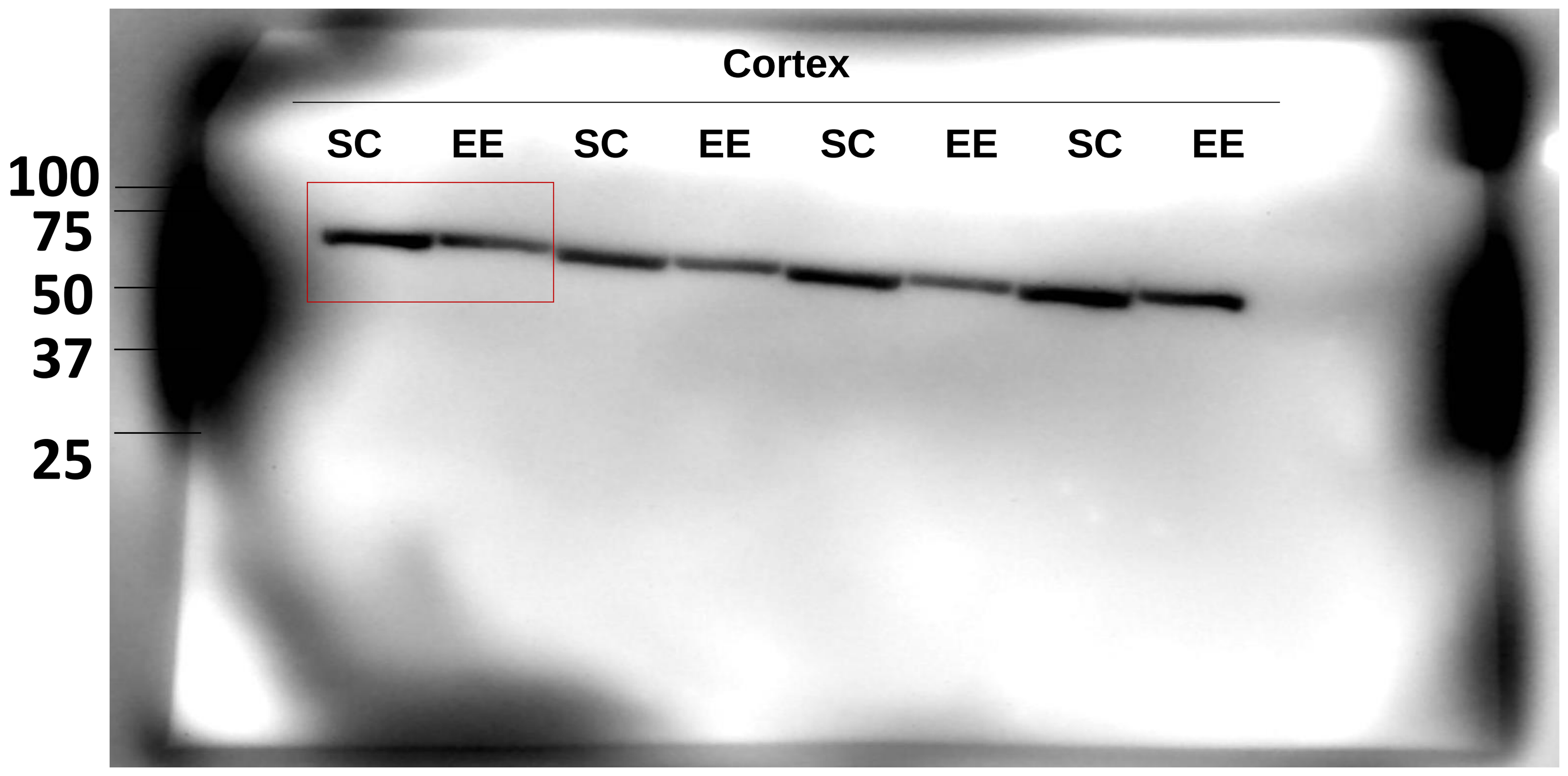

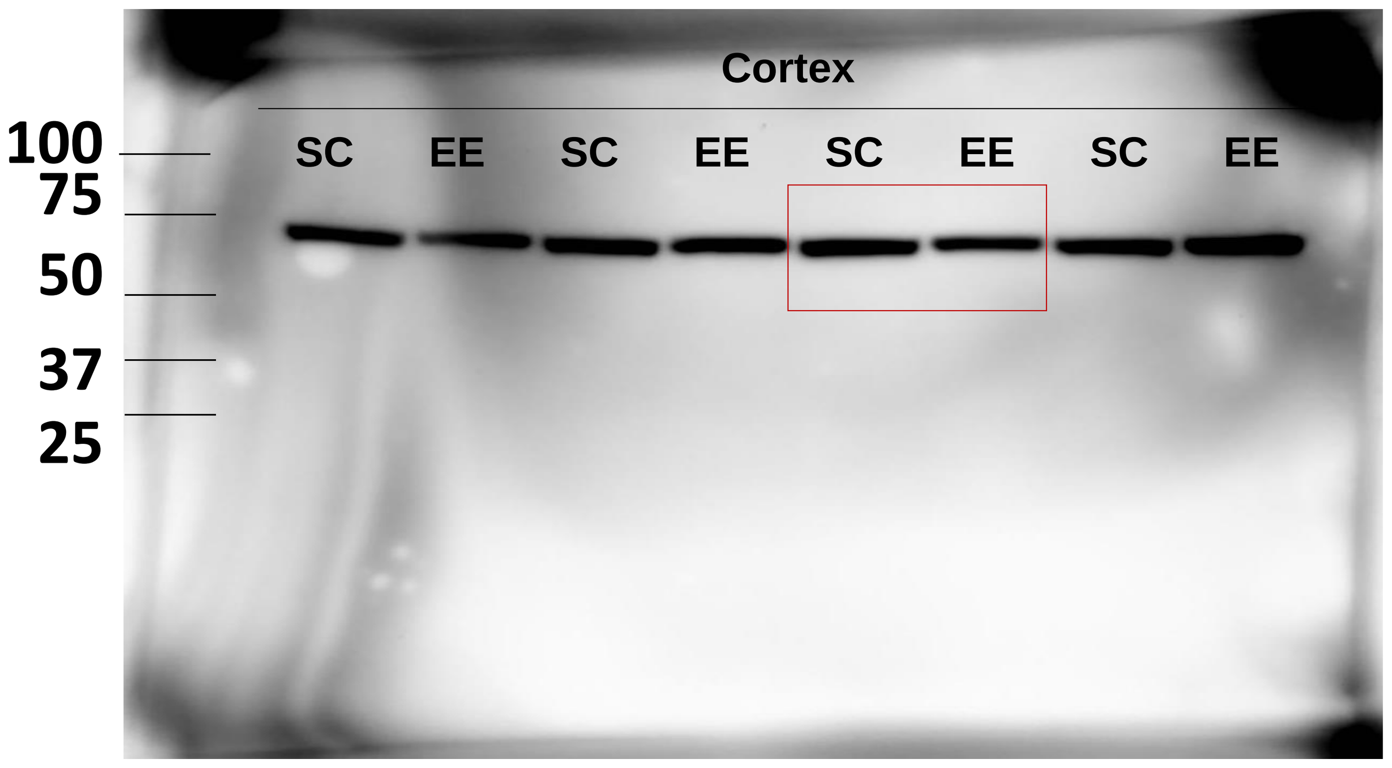

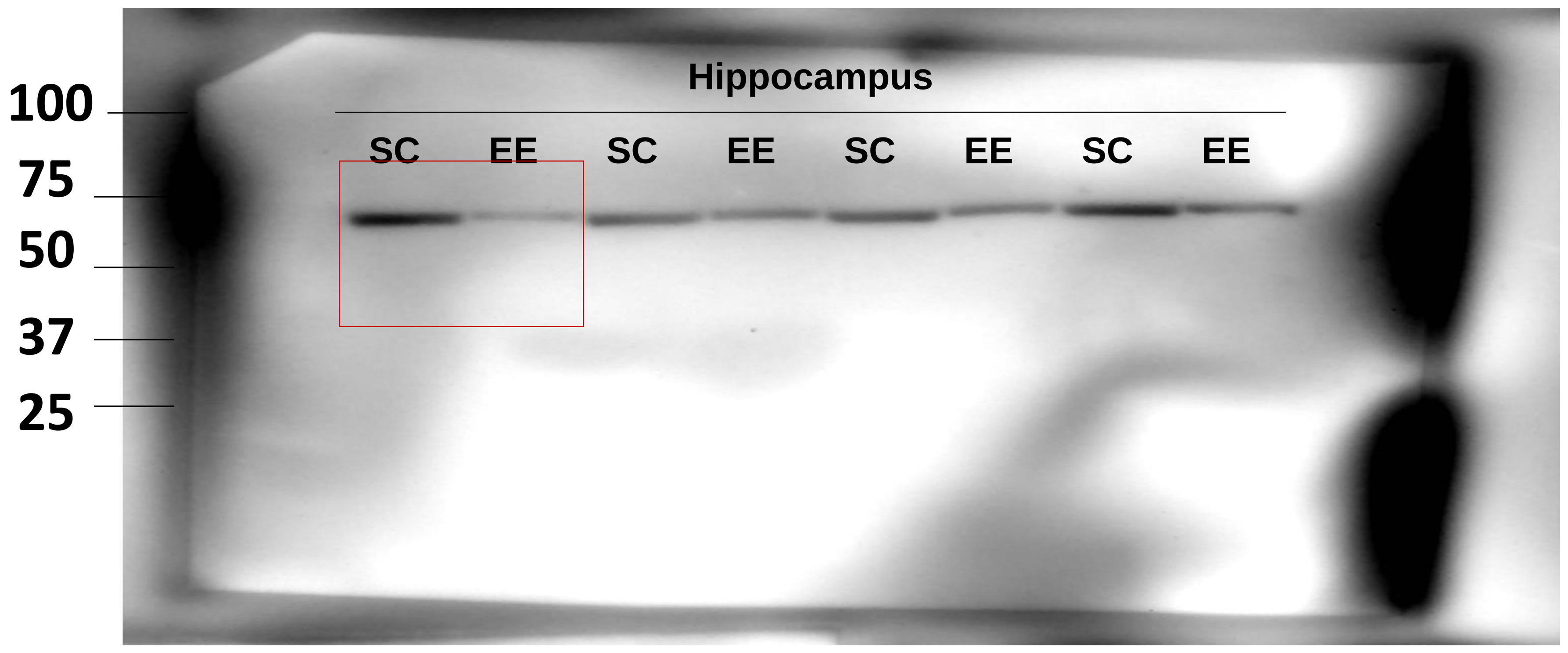

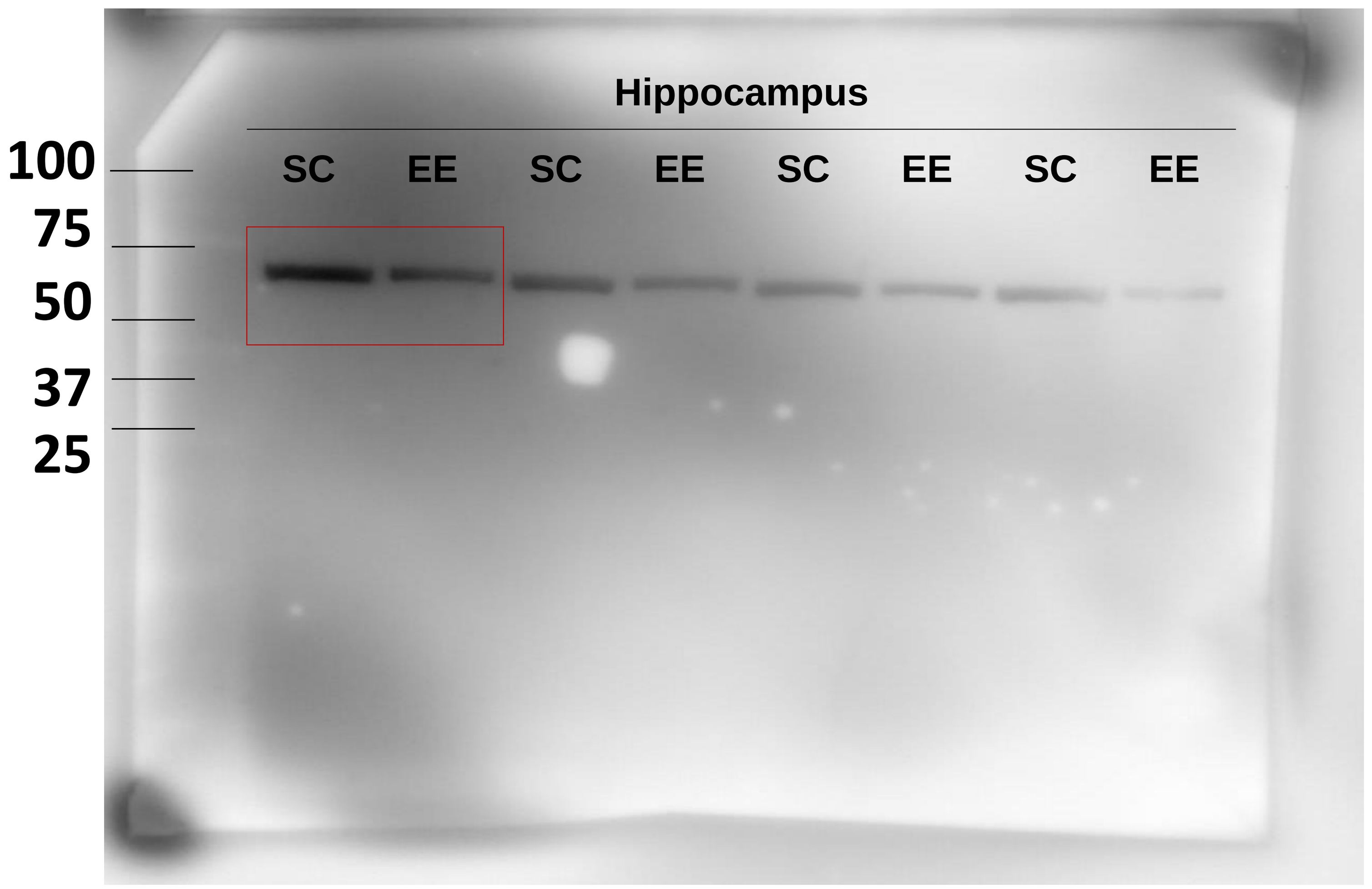

Actin (Figure 5)

Very early exposure

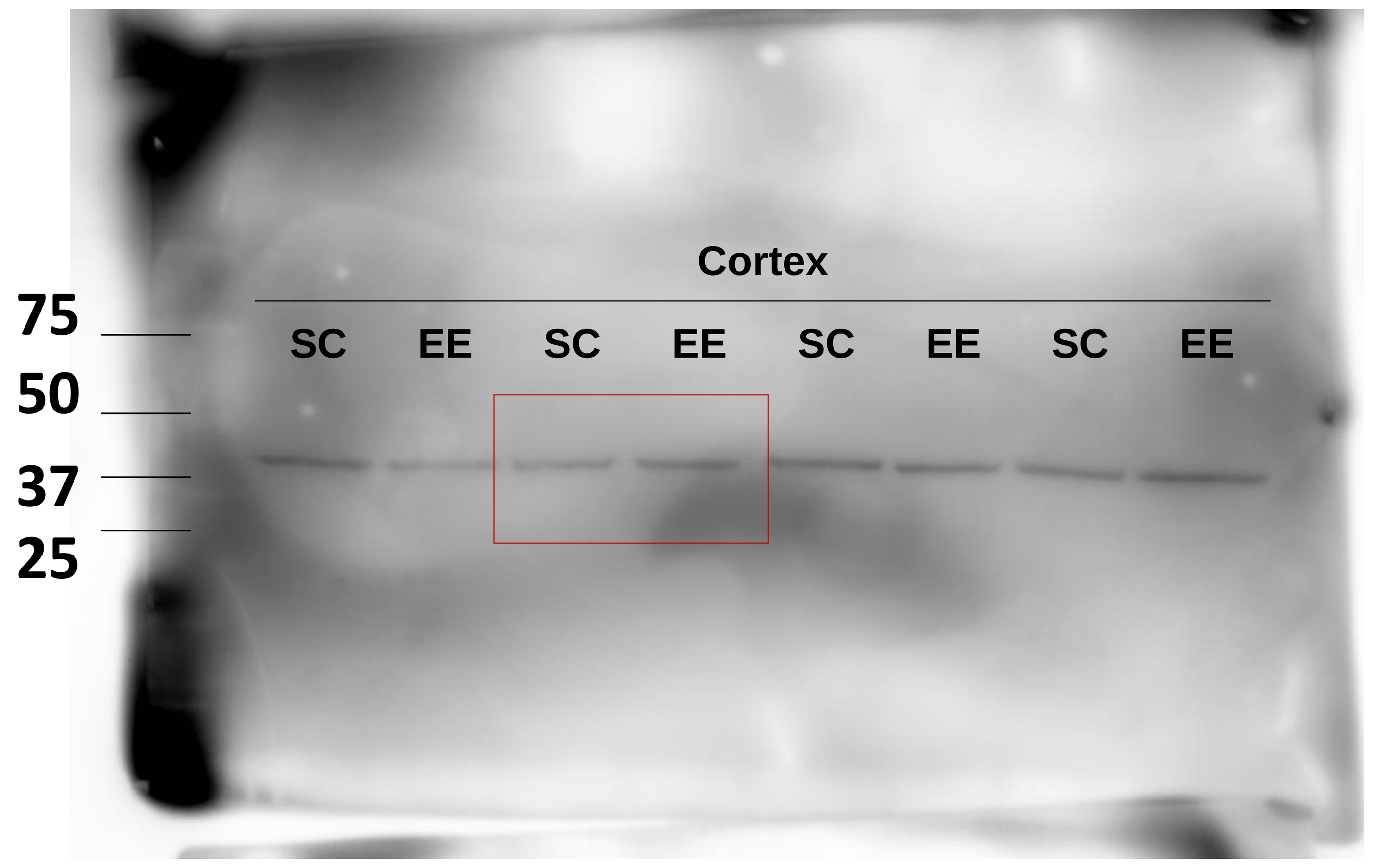

Delayed exposure

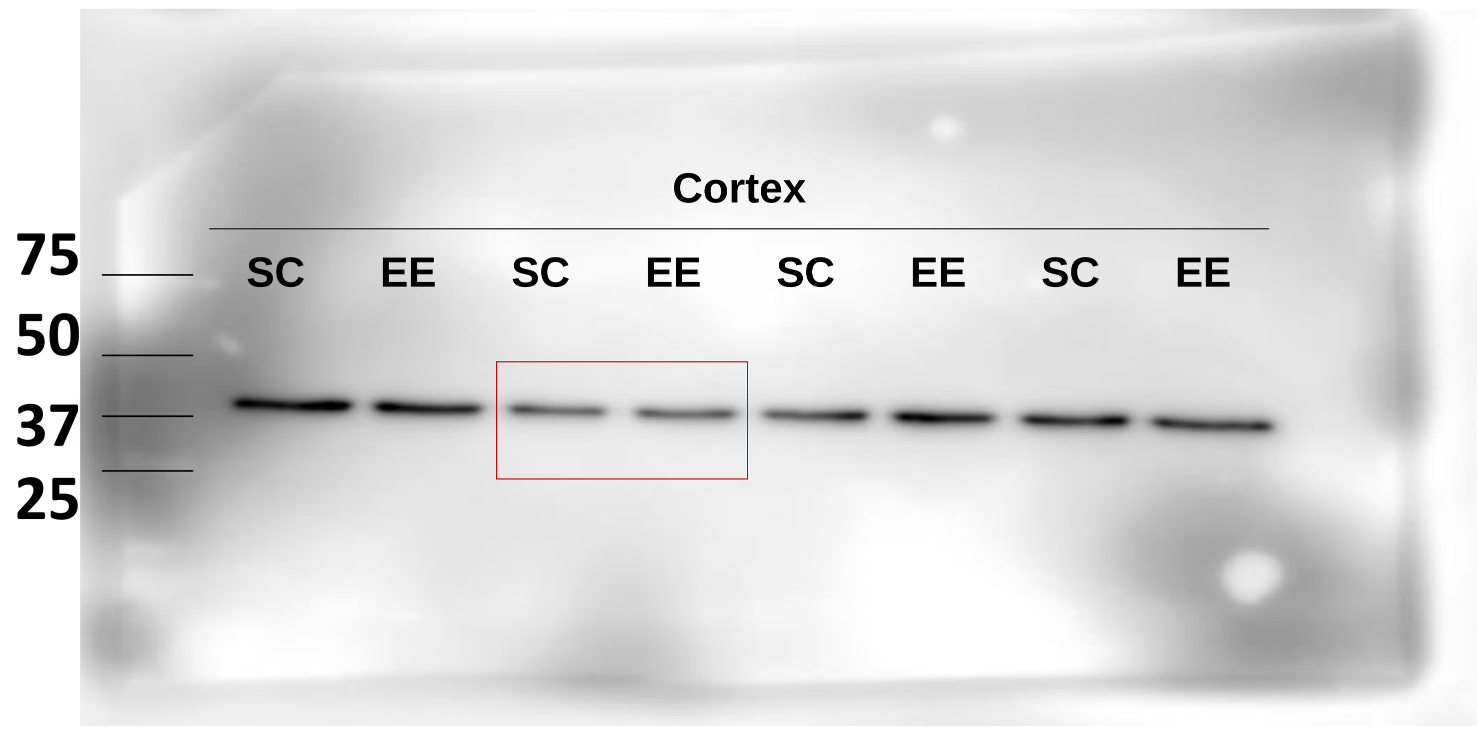

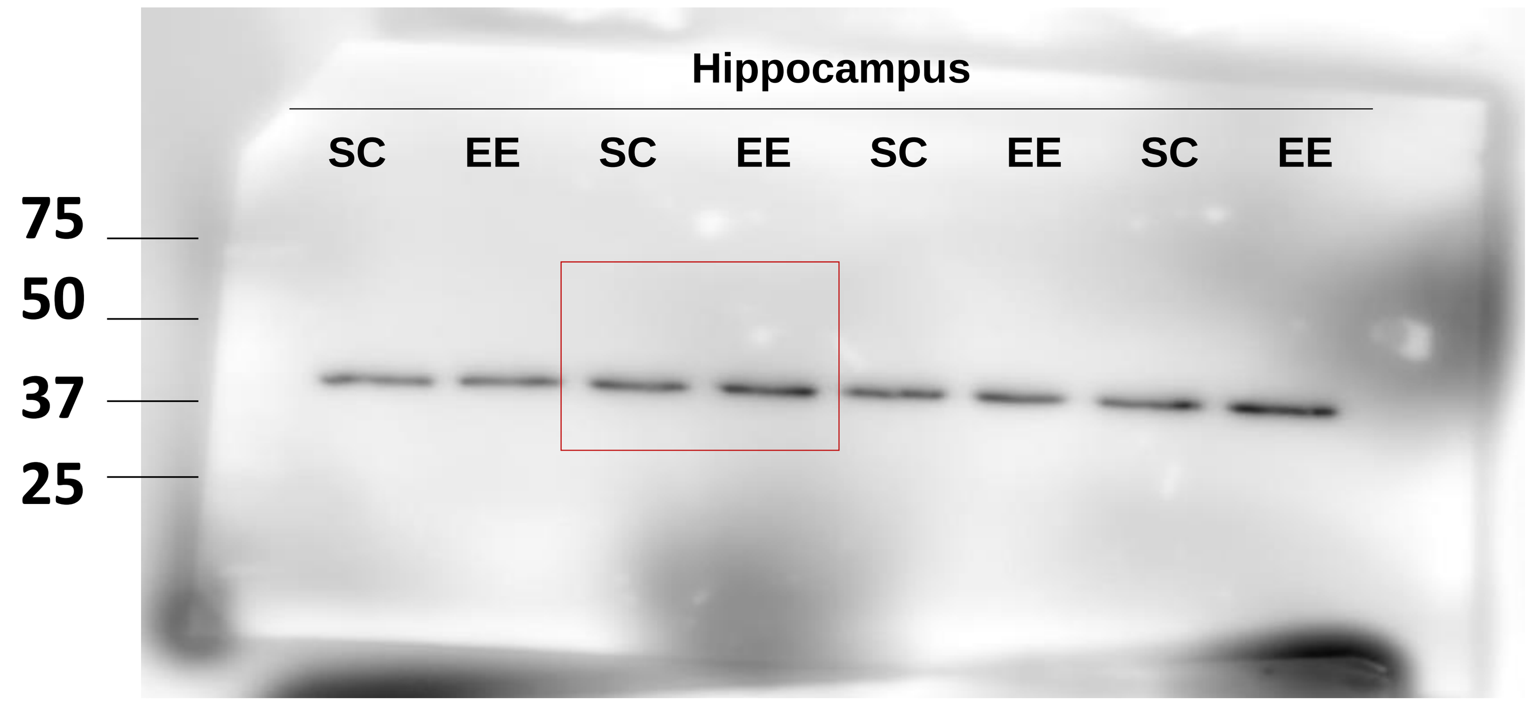

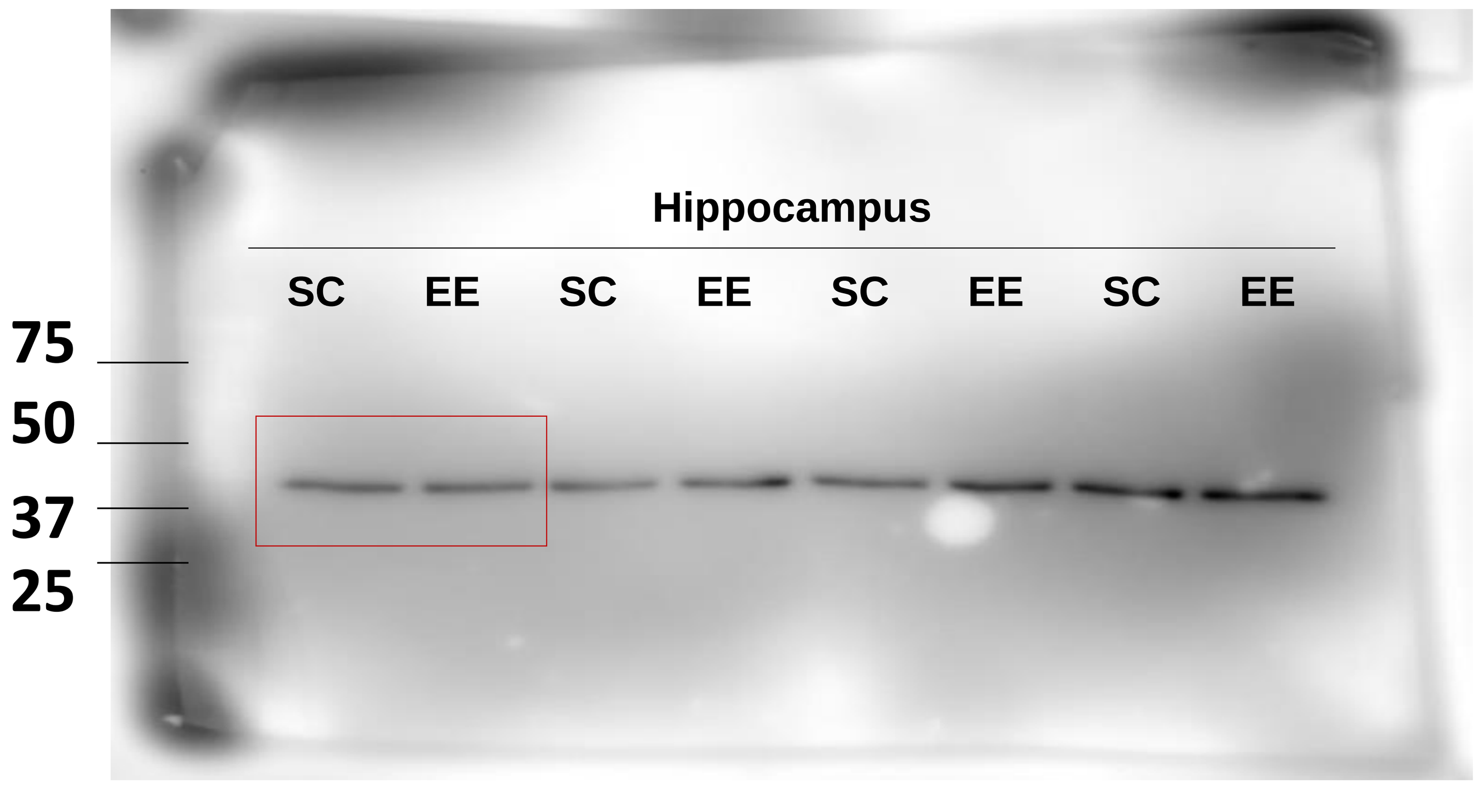

FAS (Figure 6)

Very early exposure

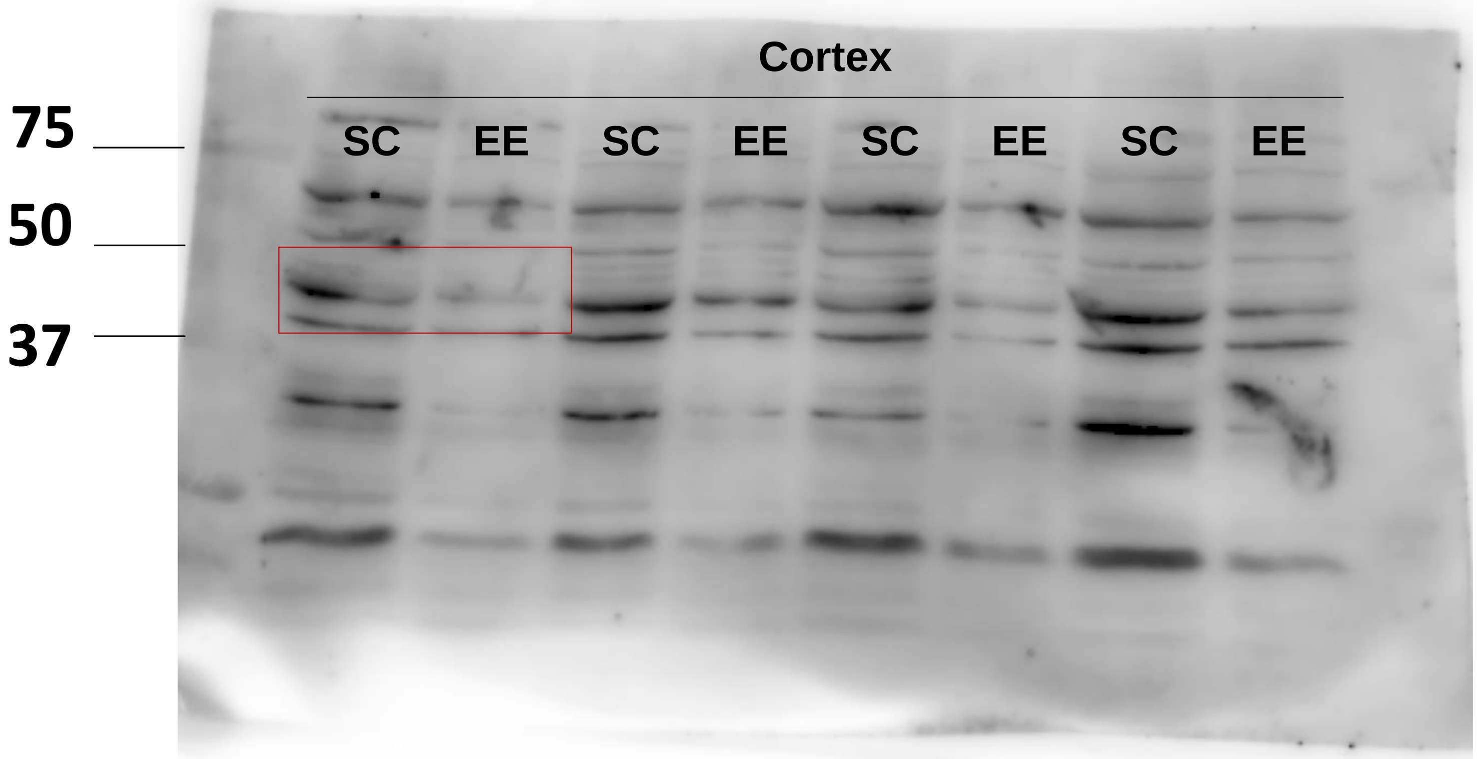

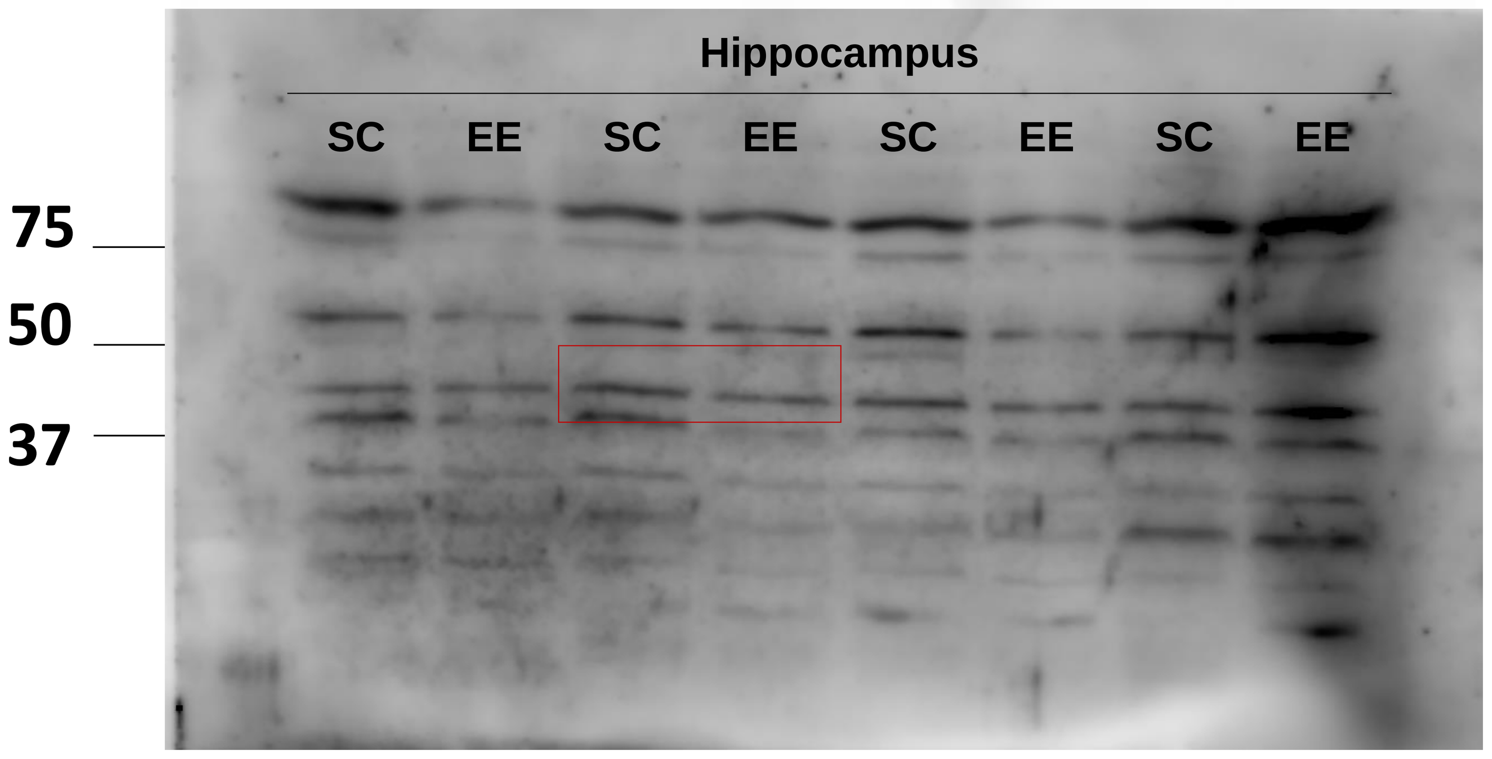

Delayed exposure

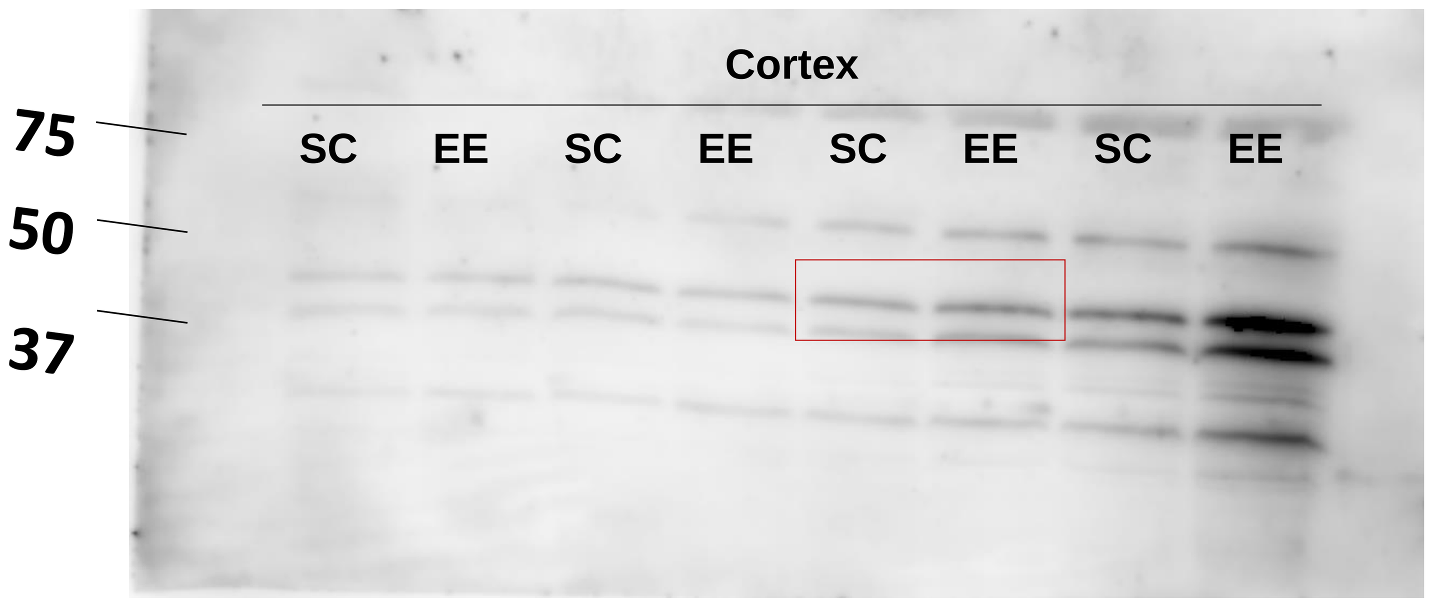

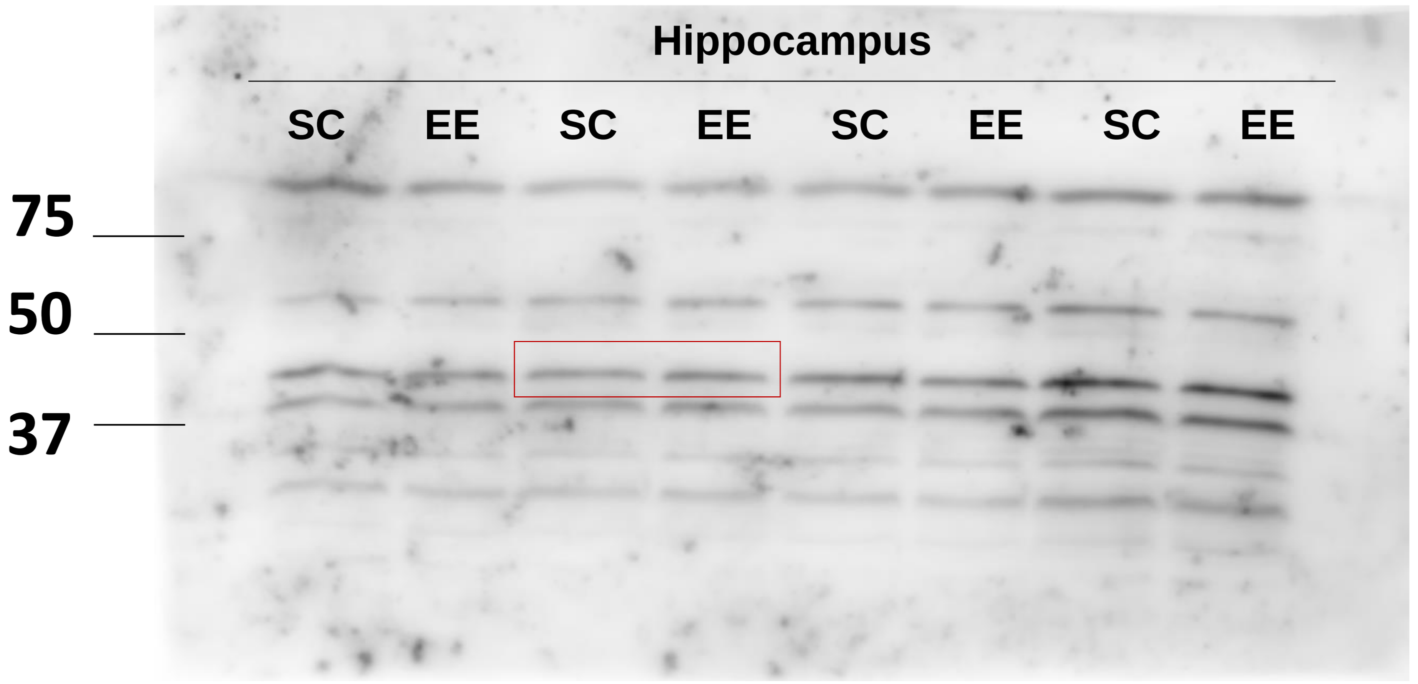

FADD (Figure 6)

Very early exposure

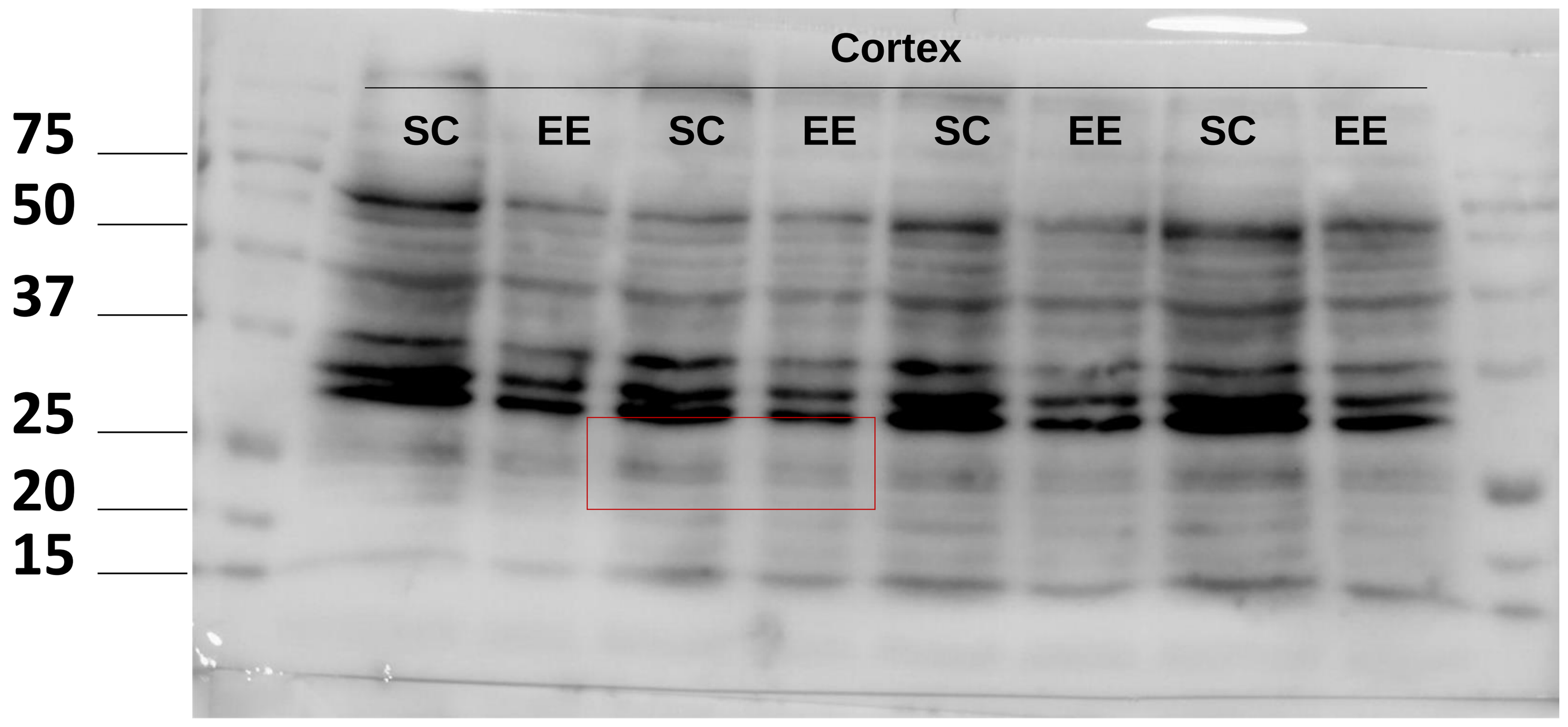

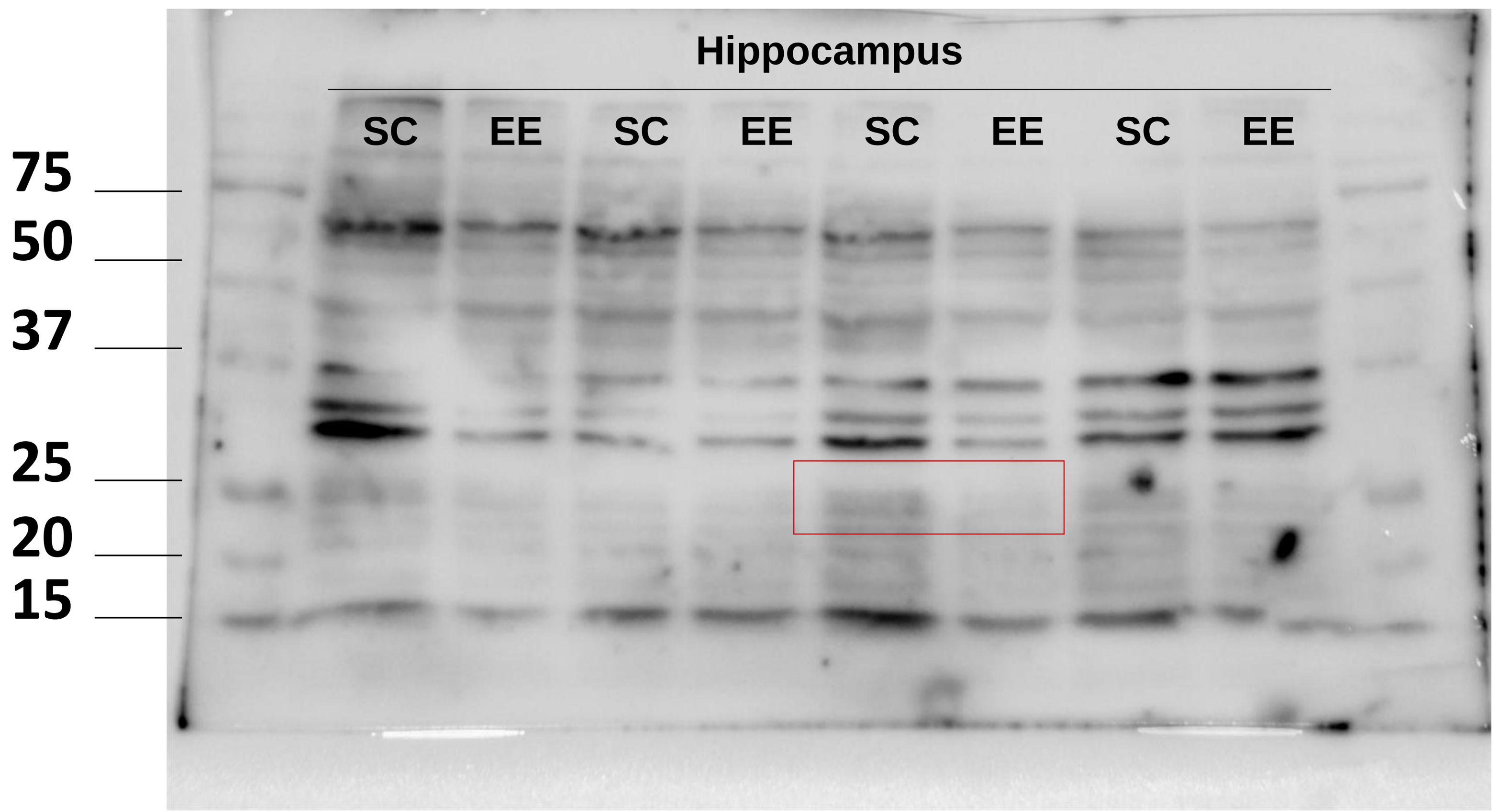

Delayed exposure

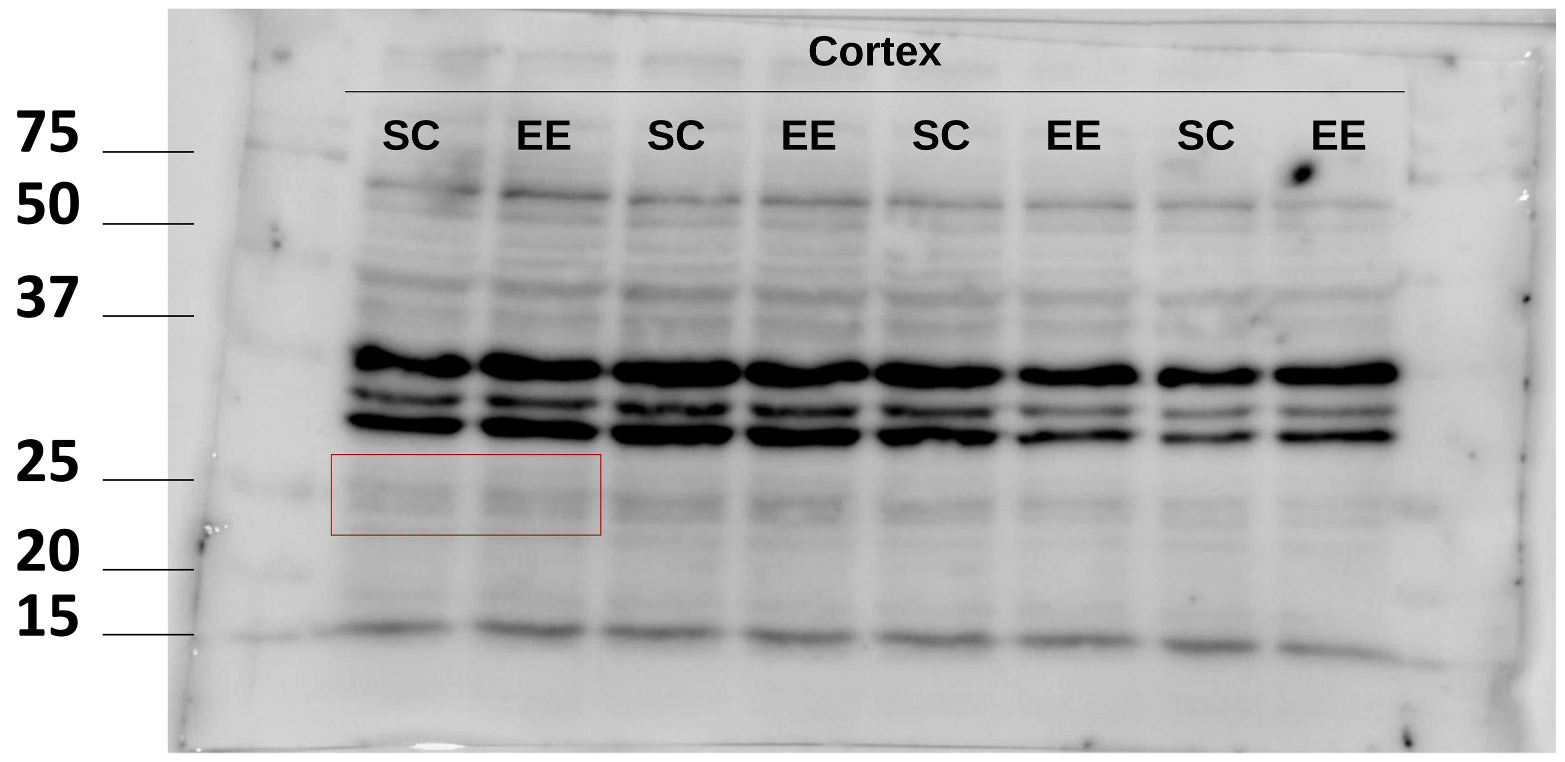

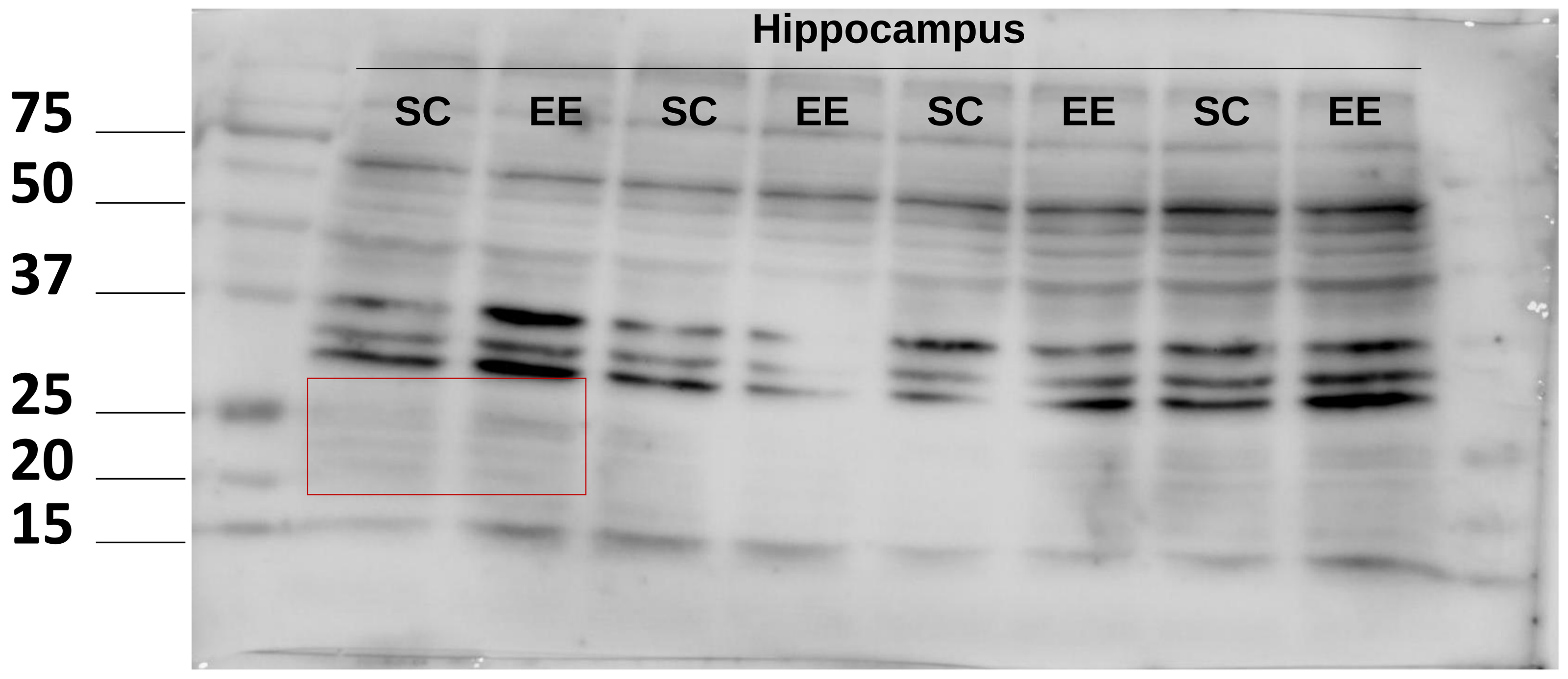

Cleaved Caspase-8 (Figure 6)

Very early exposure

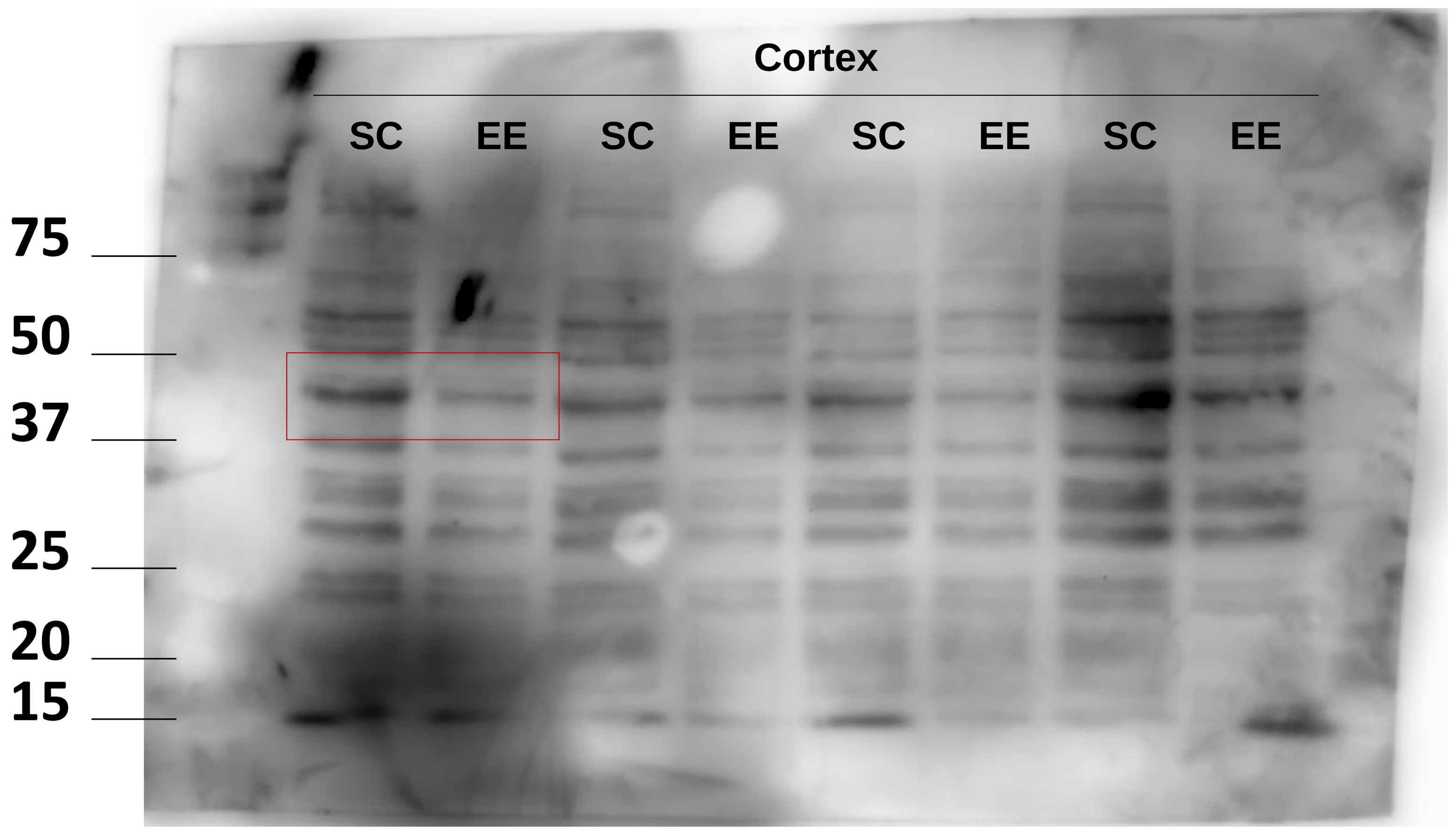

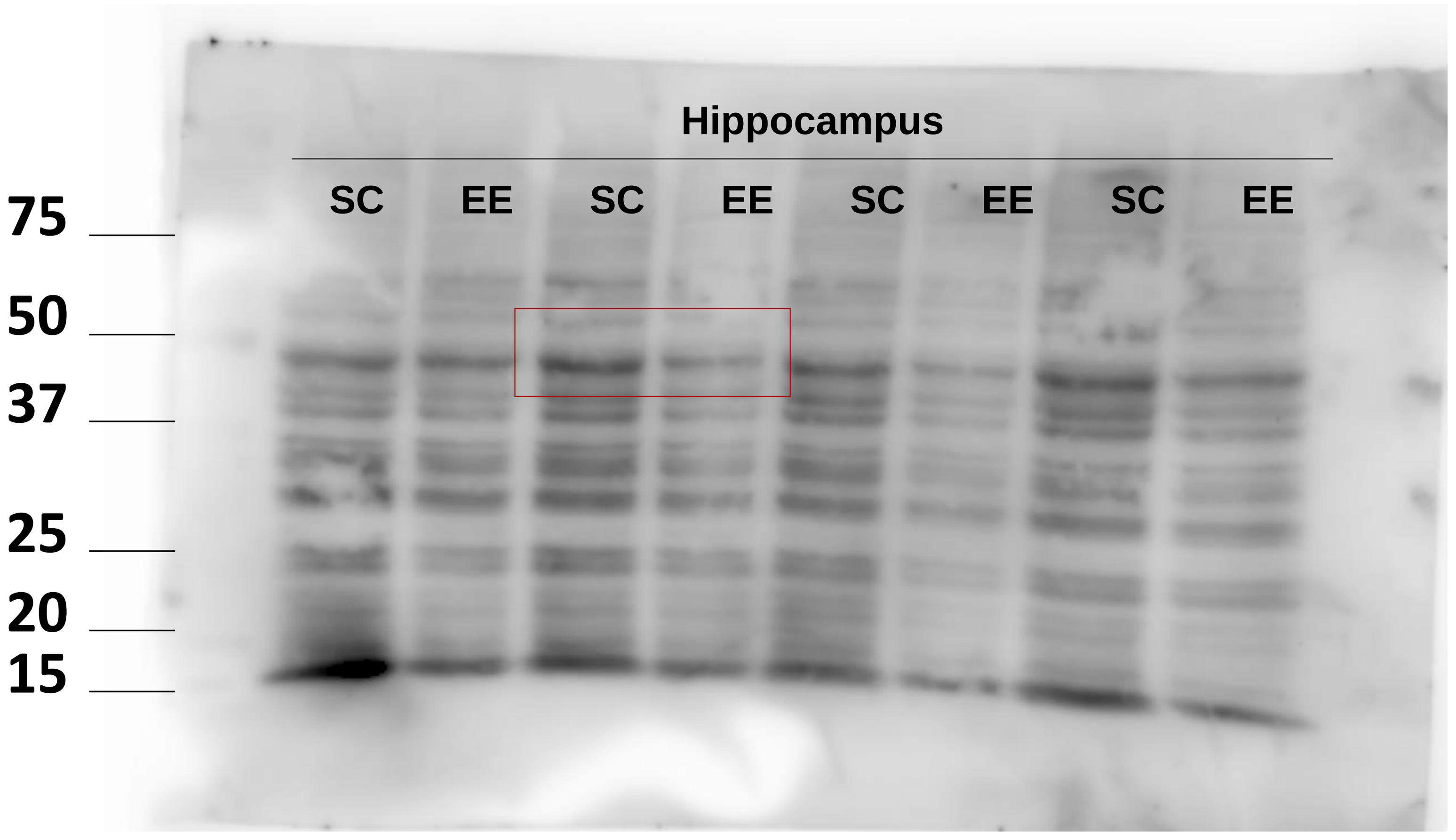

Delayed exposure

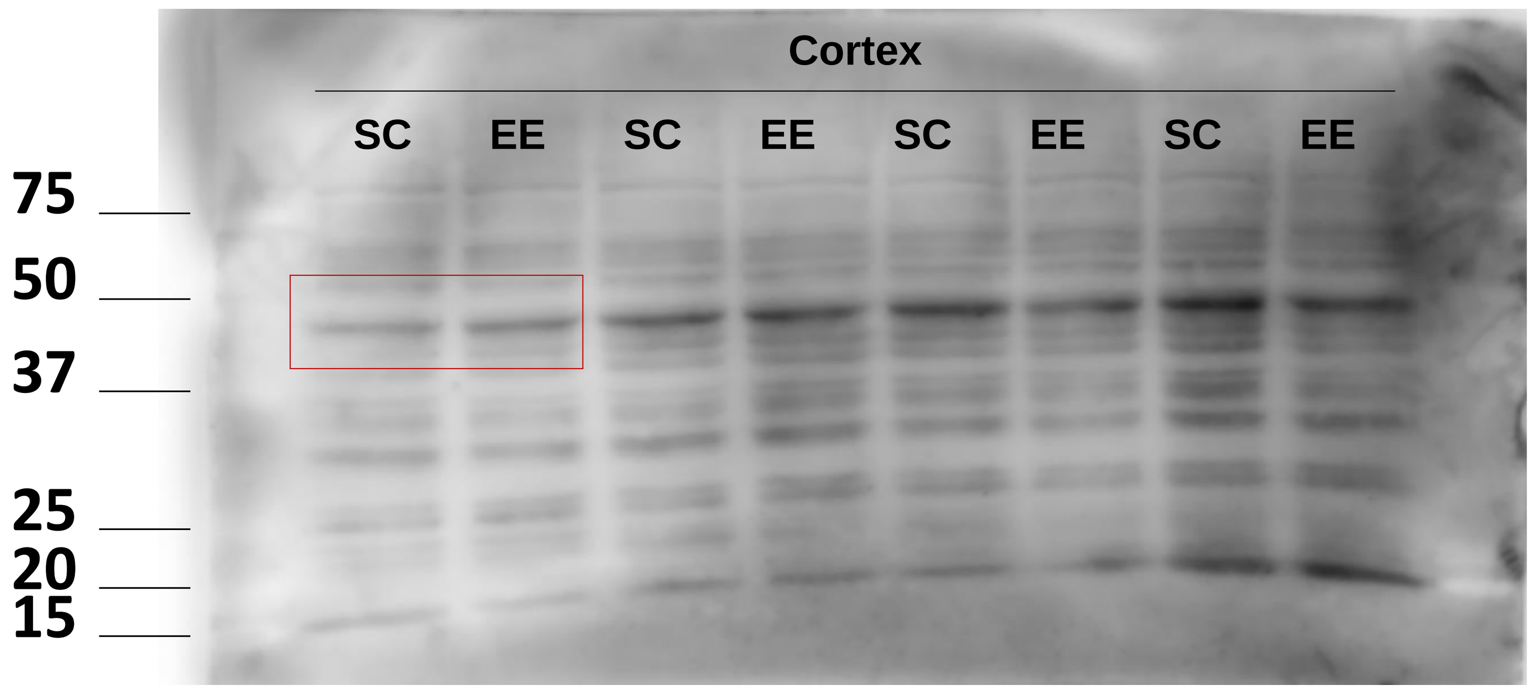

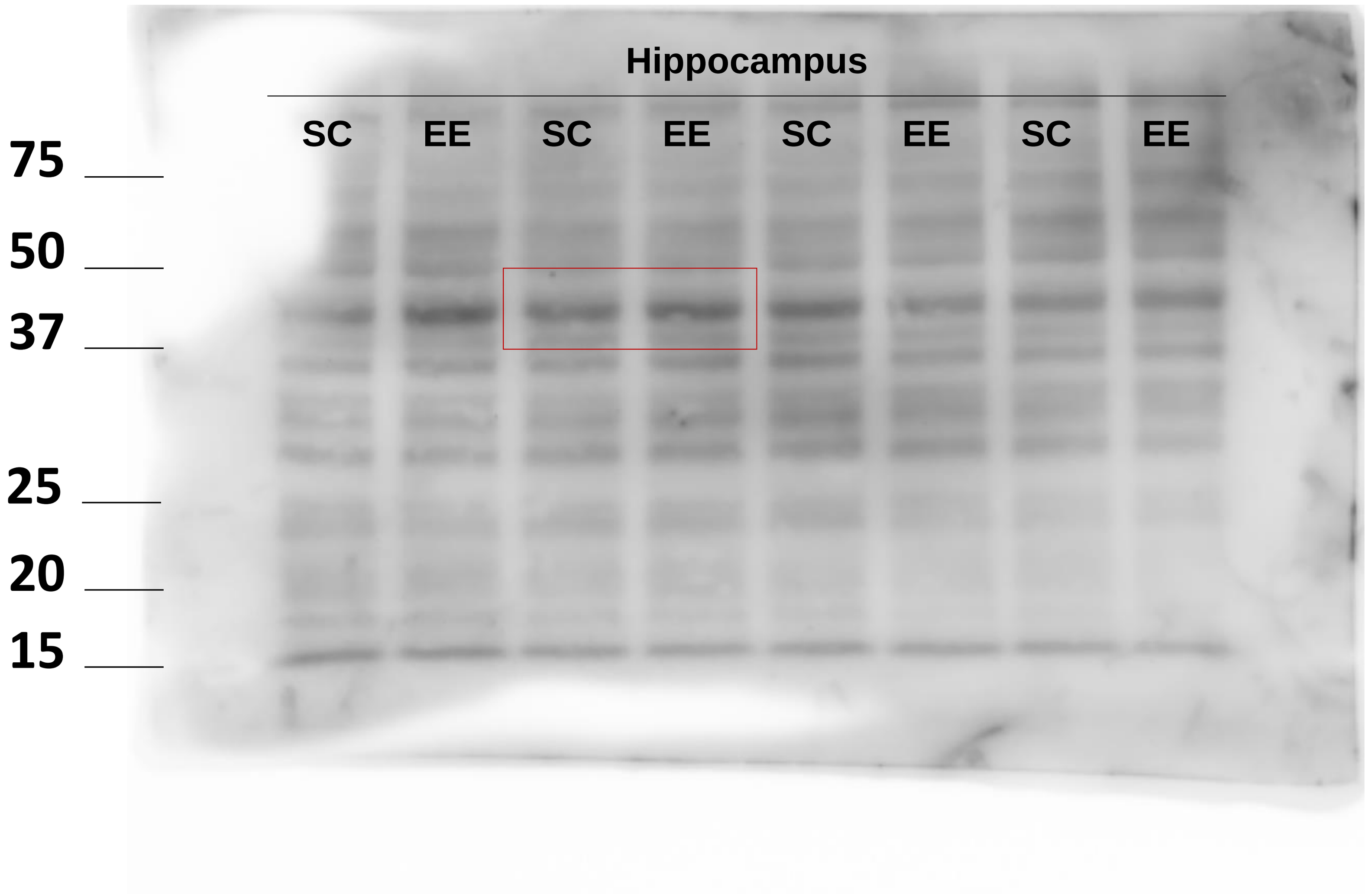

Caspase-8 (Figure 6)

Very early exposure

Delayed exposure

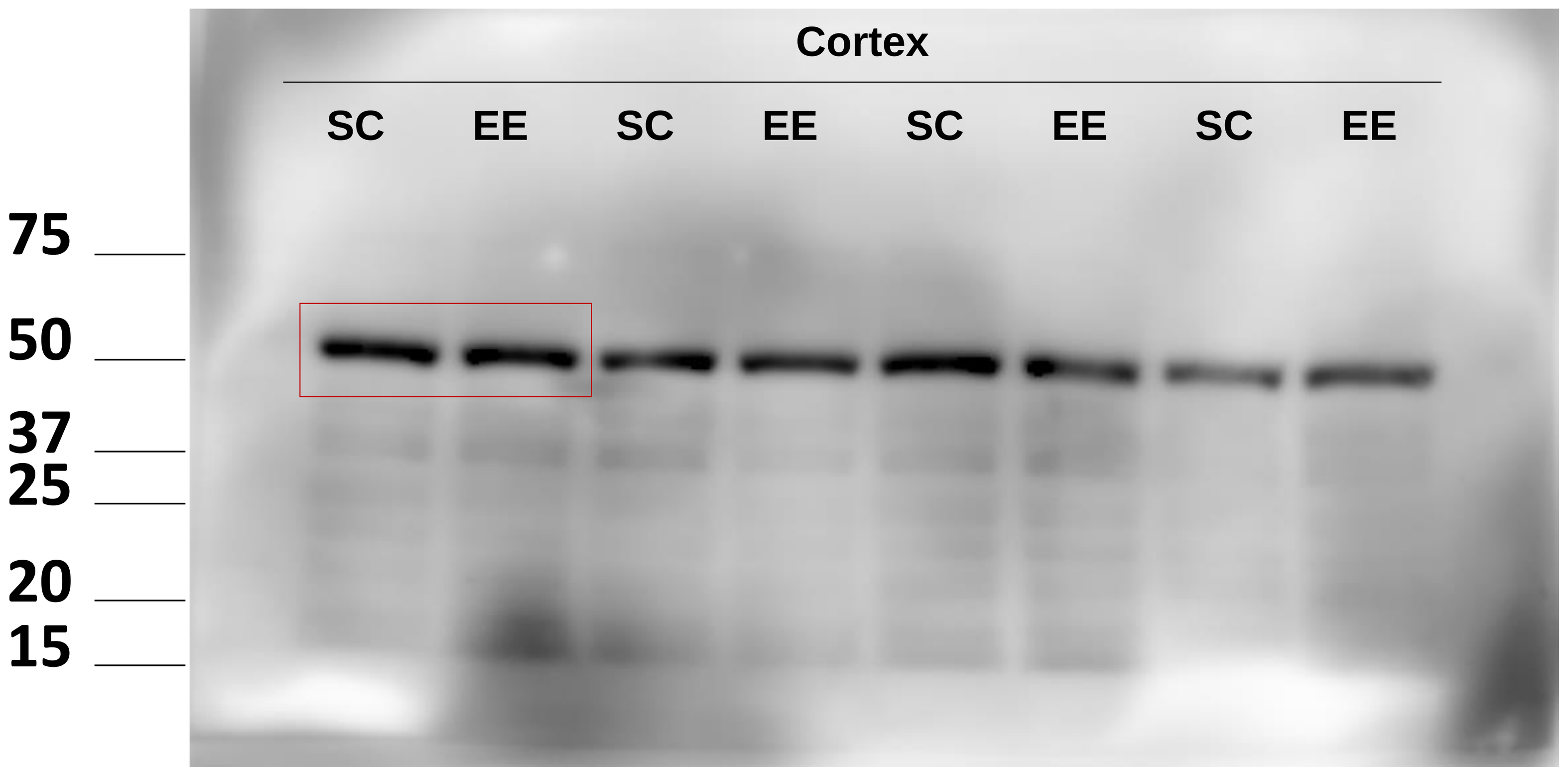

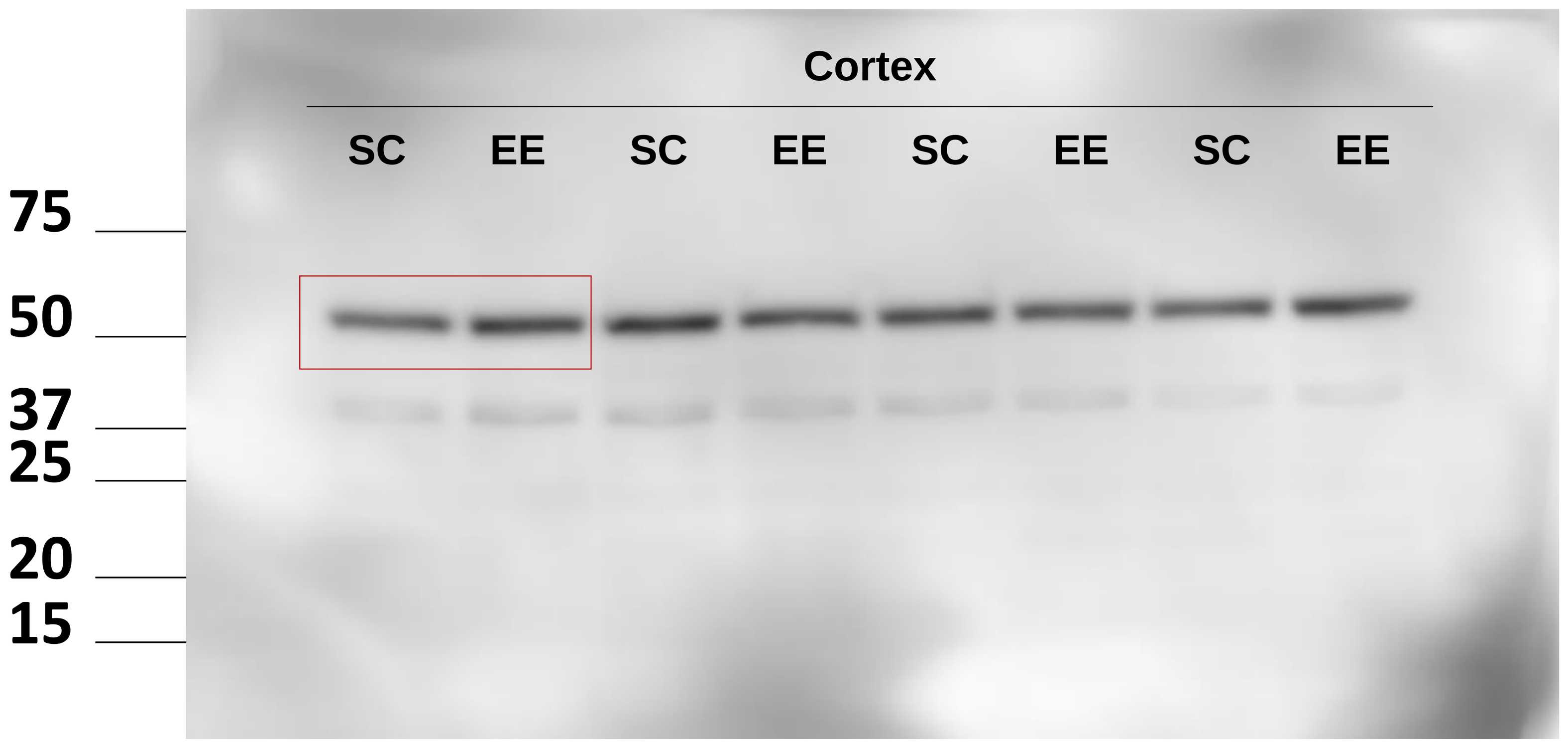

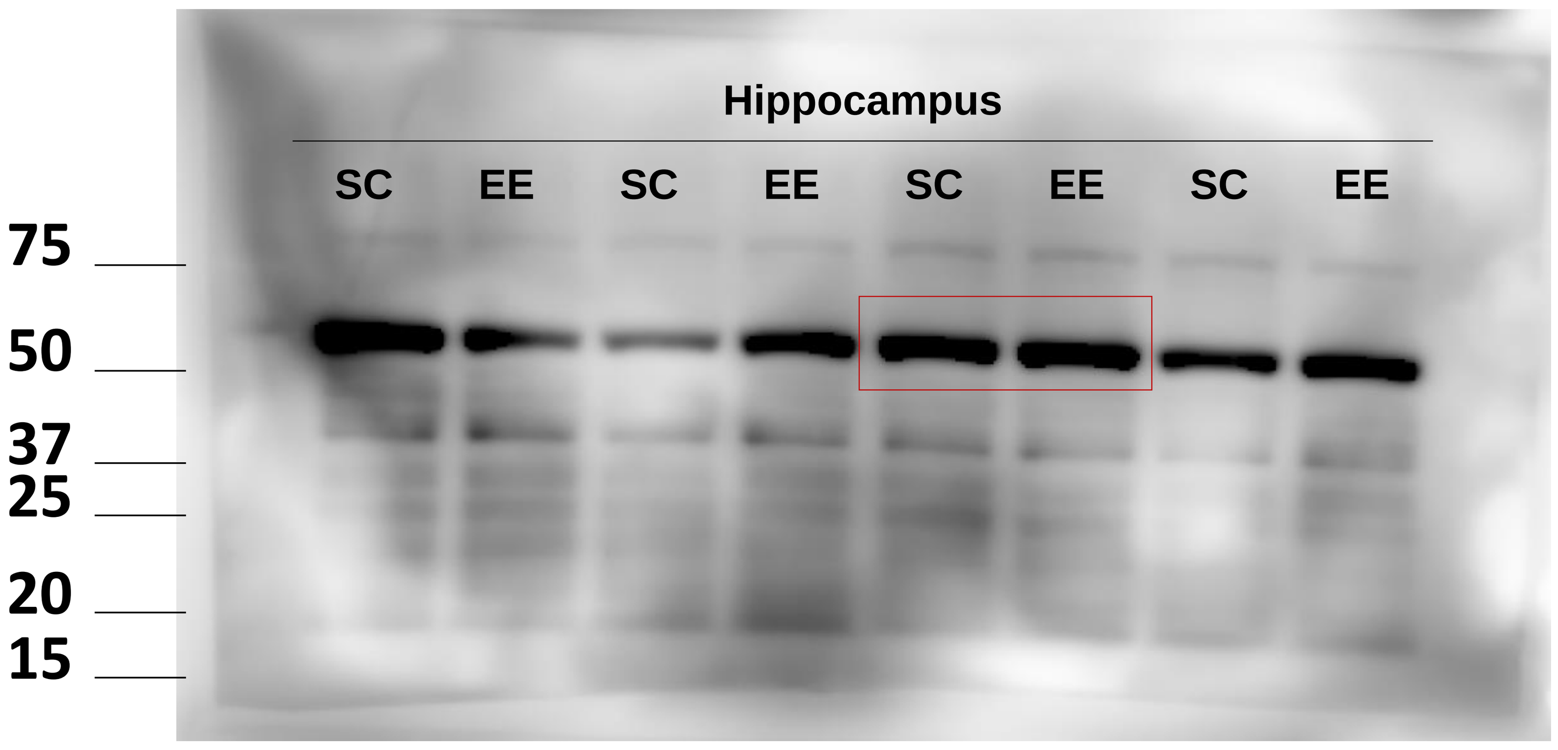

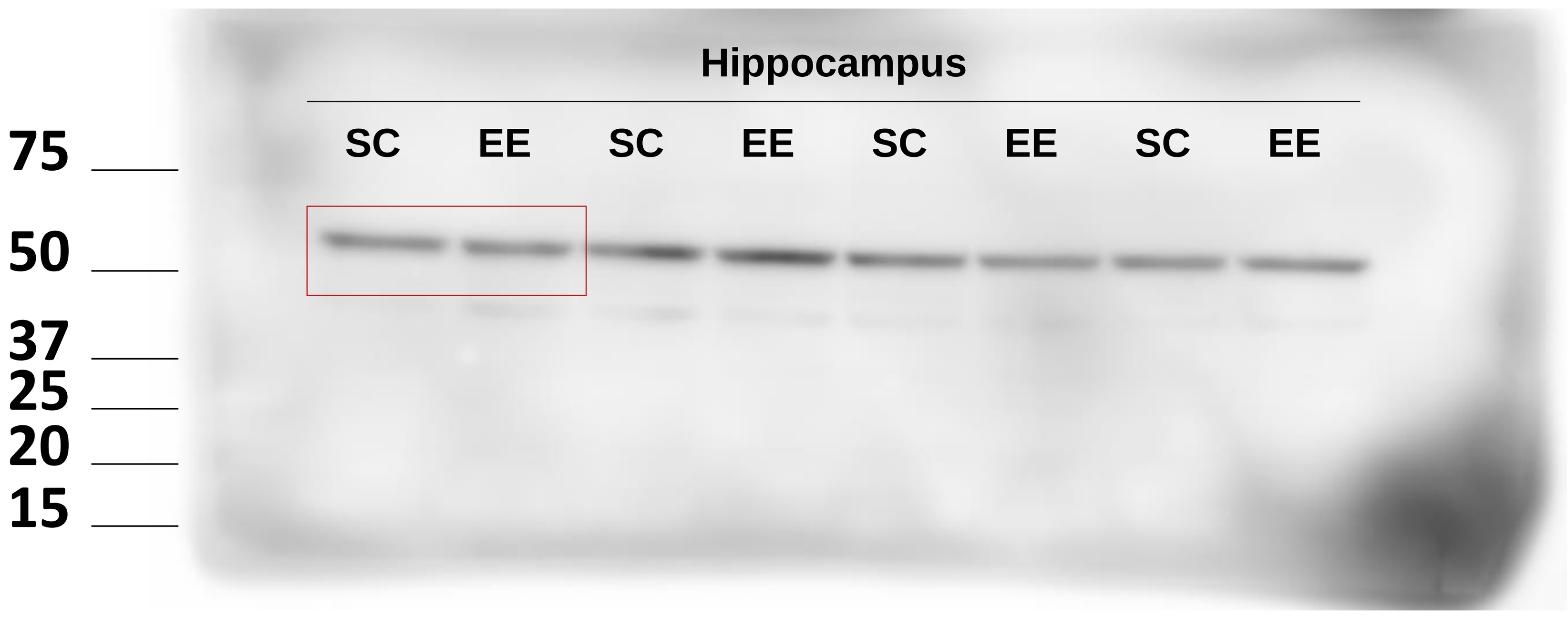

Actin (Figure 6)

Very early exposure

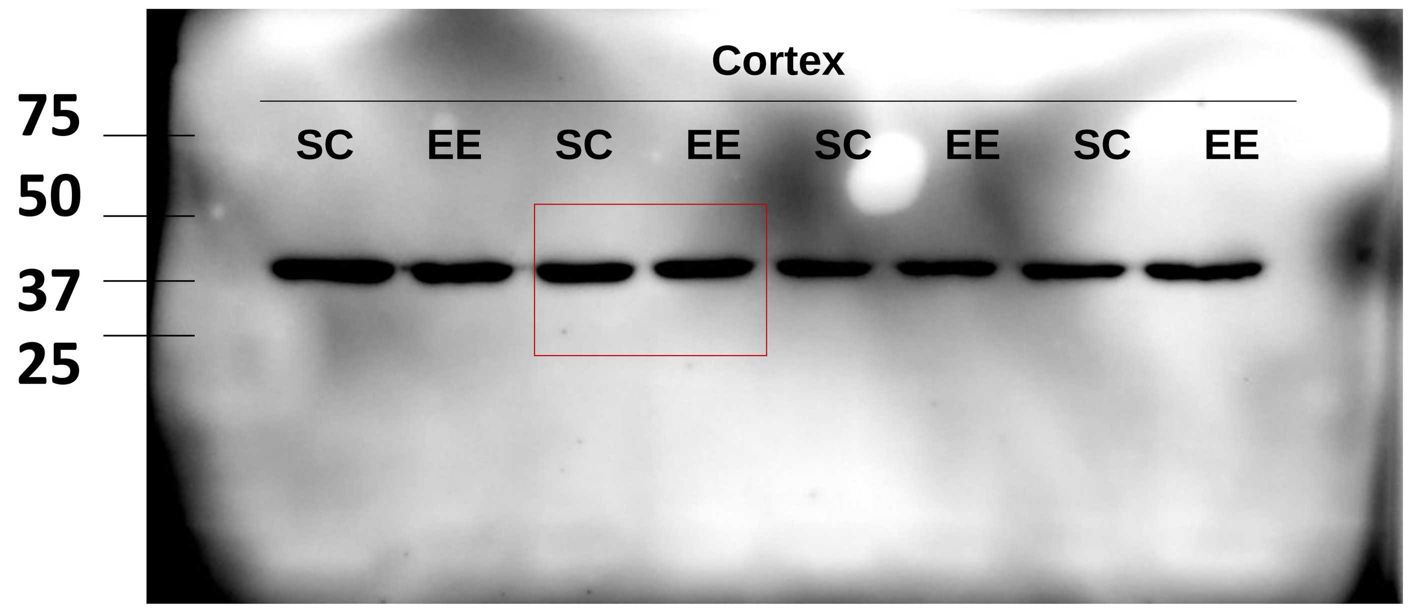

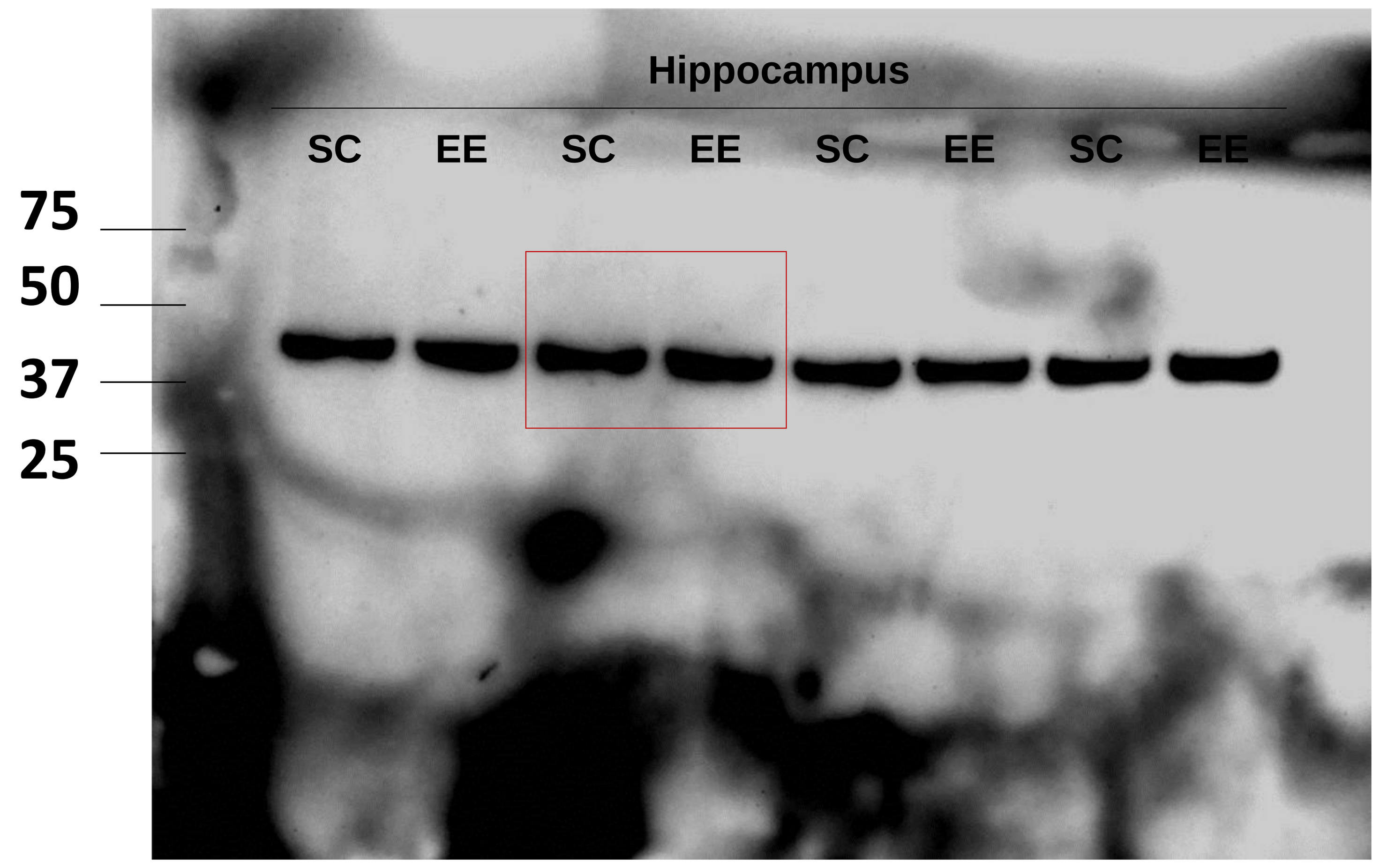

Delayed exposure

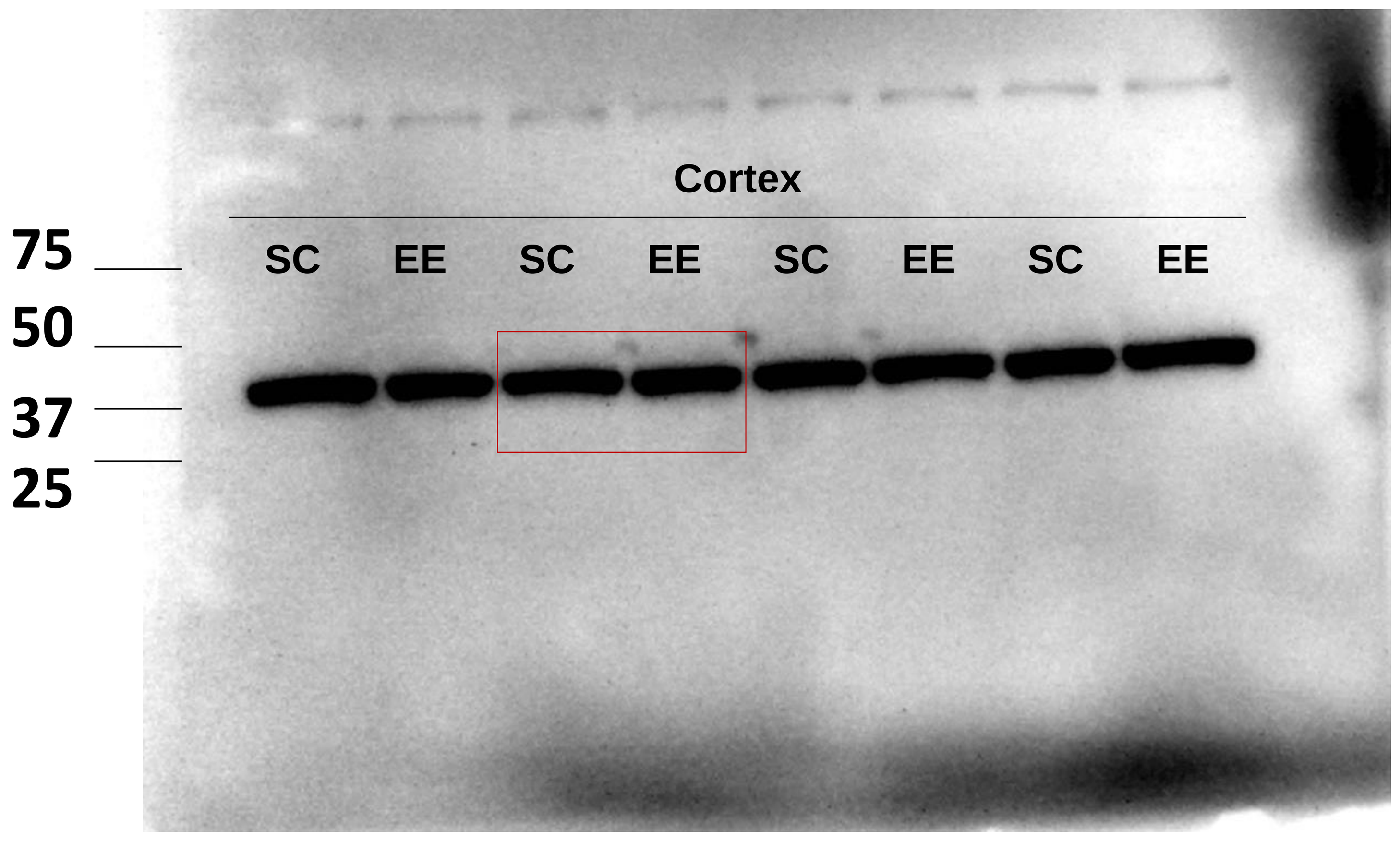

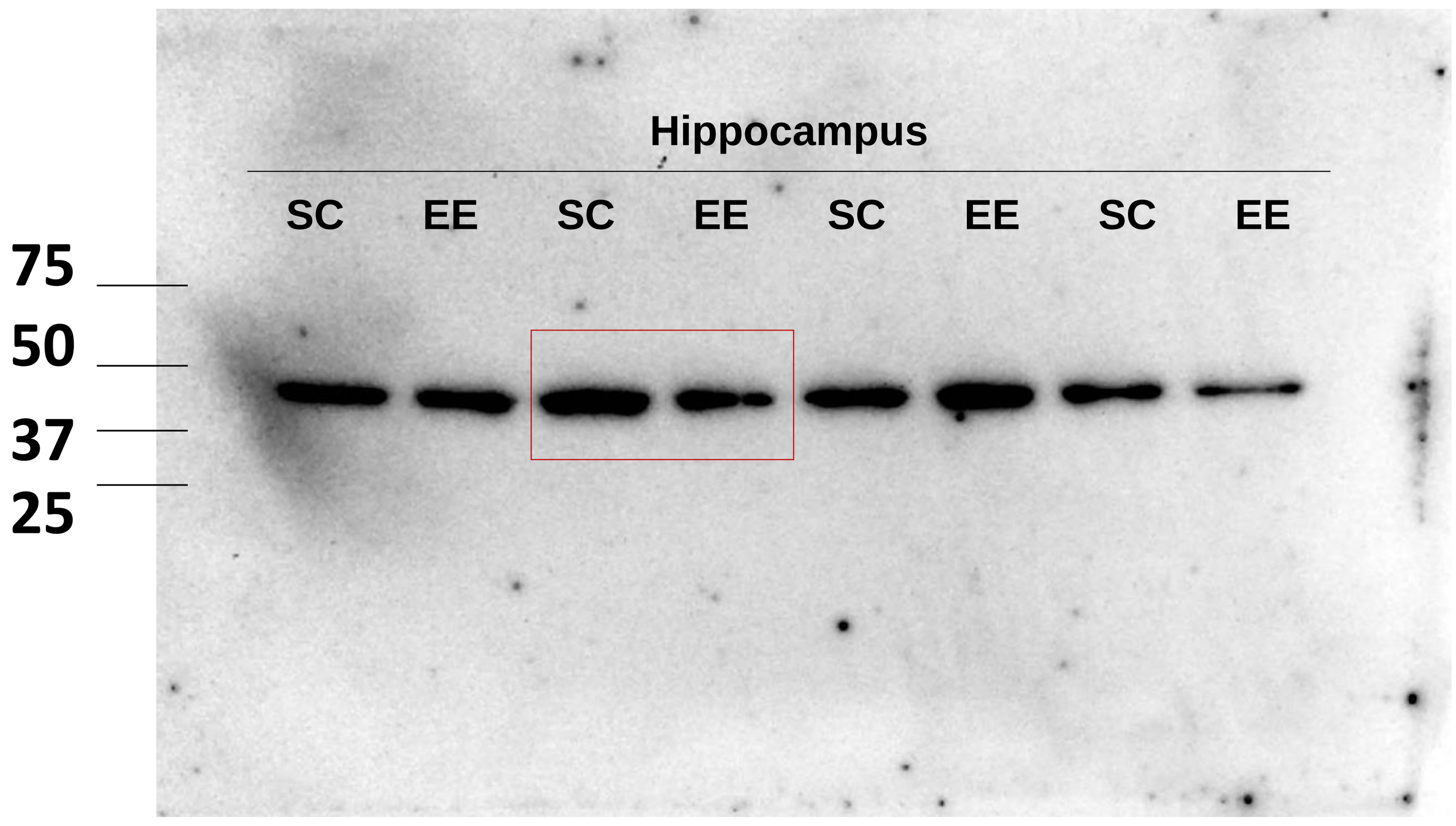

Supplement: Supplementary file 8 [file Data_Sheet_1.PDF]
