# Supplementary figures and images for: Very early environmental enrichment protects against apoptosis and improves functional recovery from hypoxic–ischemic brain injury
Source: Front Mol Neurosci. 2023 Feb 7;15:1019173. doi: 10.3389/fnmol.2022.1019173 (PMC9942523; doi:10.3389/fnmol.2022.1019173)

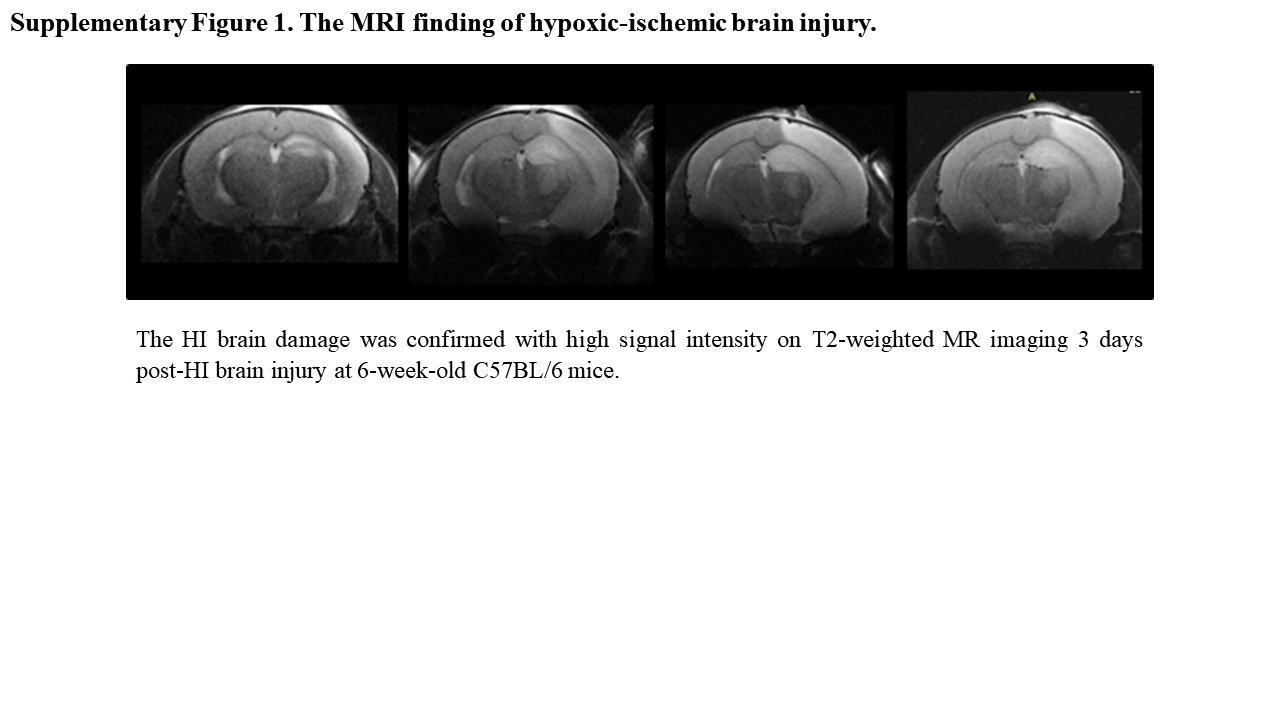

Supplement: Supplementary file 9 [file Image_1.TIF]
